# Supplementary material for: Tachykinin signaling inhibits task-specific behavioral responsiveness in honeybee workers
Source: eLife. 2021 Mar 24;10:e64830. doi: 10.7554/eLife.64830 (PMC8016481; doi:10.7554/eLife.64830)
Supplement: Figure 1—source data 1. [file elife-64830-fig1-data1.docx]

The proboscis extension response of *Apis mellifera ligustica* (AML) and *Apis cerana cerana* (ACC) worker bees to different sucrose solutions.

| **AML pollen foragers** | | | |  | **ACC pollen foragers** | | | |
| --- | --- | --- | --- | --- | --- | --- | --- | --- |
| **Concentration** | **Show PER** | **No PER** | **PER ratio** |  | **Concentration** | **Show PER** | **No PER** | **PER ratio** |
| **0.1%** | **48** | **79** | **37.80%** |  | **0.1%** | **33** | **92** | **26.40%** |
| **0.3%** | **51** | **76** | **40.16%** |  | **0.3%** | **35** | **90** | **28.00%** |
| **1.0%** | **70** | **57** | **55.12%** |  | **1.0%** | **51** | **74** | **40.80%** |
| **3.0%** | **83** | **44** | **65.35%** |  | **3.0%** | **59** | **66** | **47.20%** |
| **10.0%** | **87** | **40** | **68.50%** |  | **10.0%** | **68** | **57** | **54.40%** |
| **30.0%** | **111** | **16** | **87.40%** |  | **30.0%** | **98** | **27** | **78.40%** |
| **Pollen** | **32** | **50** | **39.02%** |  | **Pollen** | **20** | **66** | **23.26%** |
| **Larva** | **17** | **65** | **20.73%** |  | **Larva** | **11** | **75** | **12.79%** |
|  |  |  |  |  |  |  |  |  |
| **AML nectar foragers** | | | |  | **ACC nectar foragers** | | | |
| **Concentration** | **Show PER** | **No PER** | **PER ratio** |  | **Concentration** | **Show PER** | **No PER** | **PER ratio** |
| **0.1%** | **23** | **107** | **17.69%** |  | **0.1%** | **17** | **111** | **13.28%** |
| **0.3%** | **33** | **97** | **25.38%** |  | **0.3%** | **19** | **109** | **14.84%** |
| **1.0%** | **38** | **92** | **29.23%** |  | **1.0%** | **23** | **105** | **17.97%** |
| **3.0%** | **44** | **86** | **33.85%** |  | **3.0%** | **29** | **99** | **22.66%** |
| **10.0%** | **59** | **71** | **45.38%** |  | **10.0%** | **44** | **84** | **34.38%** |
| **30.0%** | **68** | **62** | **52.31%** |  | **30.0%** | **55** | **73** | **42.97%** |
| **Pollen** | **11** | **74** | **12.94%** |  | **Pollen** | **8** | **77** | **9.41%** |
| **Larva** | **15** | **70** | **17.65%** |  | **Larva** | **9** | **76** | **10.59%** |
|  |  |  |  |  |  |  |  |  |
| **AML nurse bees** | | | |  | **ACC nurse bees** | | | |
| **Concentration** | **Show PER** | **No PER** | **PER ratio** |  | **Concentration** | **Show PER** | **No PER** | **PER ratio** |
| **0.1%** | **30** | **106** | **22.06%** |  | **0.1%** | **18** | **113** | **13.74%** |
| **0.3%** | **32** | **104** | **23.53%** |  | **0.3%** | **19** | **112** | **14.50%** |
| **1.0%** | **45** | **91** | **33.09%** |  | **1.0%** | **30** | **101** | **22.90%** |
| **3.0%** | **50** | **86** | **36.76%** |  | **3.0%** | **38** | **93** | **29.01%** |
| **10.0%** | **57** | **79** | **41.91%** |  | **10.0%** | **48** | **83** | **36.64%** |
| **30.0%** | **75** | **61** | **55.15%** |  | **30.0%** | **58** | **73** | **44.27%** |
| **Pollen** | **9** | **82** | **9.89%** |  | **Pollen** | **7** | **81** | **7.95%** |
| **Larva** | **36** | **55** | **39.56%** |  | **Larva** | **22** | **66** | **25.00%** |
