# Supplementary material for: Tachykinin signaling inhibits task-specific behavioral responsiveness in honeybee workers
Source: eLife. 2021 Mar 24;10:e64830. doi: 10.7554/eLife.64830 (PMC8016481; doi:10.7554/eLife.64830)
Supplement: Figure 2—source data 1. [file elife-64830-fig2-data1.docx]

Quantitative neuropeptide comparison of different behavioral phenotypes of *Apis mellifera ligustica* workers. (manuscript section 2.2)

"**Protein Accession**": the unique number given to mark the entry of a protein in the database NCBInr. "**Peptide**": the amino acid sequence of the peptide. "**Significance (-10lgP)**": the peptide confidence score. "**NBs**": nurse bees. "**PFs**": pollen foragers. "**NFs**": nectar foragers. "**Group Profile (Ratio)**": the relative abundance ratio to the base group. "**PTM**": the post translational modification types present in the peptide.

| **Protein** | **Protein Accession** | **Peptide** | **Significance** | **NBs 1** | **NBs 2** | **NBs 3** | **PFs 1** | **PFs 2** | **PFs 3** | **NBs** | **PFs** | **Group Profile (Ratio)** | **PTM** |
| --- | --- | --- | --- | --- | --- | --- | --- | --- | --- | --- | --- | --- | --- |
| **PBAN-type neuropeptides (PBAN)** | **A8CL69.1** | QITQFTPRLa | 60 | 6.51E+06 | 6.29E+06 | 6.44E+06 | 4.23E+07 | 4.34E+07 | 4.42E+07 | 6.41E+06 | 4.33E+07 | 1.00 : 6.76 | Amidation |
|  |  | TSQDITSGMWFGPRLa | 60 | 6.88E+08 | 6.55E+08 | 6.65E+08 | 1.88E+09 | 1.85E+09 | 1.95E+09 | 6.69E+08 | 1.89E+09 | 1.00 : 2.83 | Amidation |
|  |  | MWFGPRLa | 27.89 | 2.04E+06 | 2.04E+06 | 2.15E+06 | 4.40E+05 | 4.64E+05 | 4.47E+05 | 2.08E+06 | 4.50E+05 | 1.00 : 0.22 | Amidation |
| **FMRFamide** | **ACI90290.1** | TWKSPDIVIRFa | 60 | 1.81E+07 | 1.85E+07 | 1.69E+07 | 4.27E+07 | 4.30E+07 | 4.18E+07 | 1.78E+07 | 4.25E+07 | 1.00 : 2.38 | Amidation |
|  |  | GRNDLNFIRYa | 42.6 | 2.94E+06 | 3.09E+06 | 3.11E+06 | 4.77E+06 | 4.84E+06 | 4.44E+06 | 3.05E+06 | 4.68E+06 | 1.00 : 1.54 | Amidation |
| **Myosuppressin** | **P85527.1** | pQDVDHVFLRFa | 30.88 | 1.33E+08 | 1.21E+08 | 1.28E+08 | 3.48E+08 | 3.65E+08 | 3.39E+08 | 1.27E+08 | 3.51E+08 | 1.00 : 2.75 | Pyro-glu from Q; Amidation |
|  |  | pQDVDHVFLR | 60 | 1.23E+07 | 1.33E+07 | 1.39E+07 | 6.40E+06 | 6.49E+06 | 6.72E+06 | 1.32E+07 | 6.54E+06 | 1.00 : 0.5 | Pyro-glu from Q |
| **Prohormone-3** | **P85828.1** | SLKAPFA | 60 | 9.06E+06 | 9.13E+06 | 8.90E+06 | 1.88E+07 | 2.00E+07 | 2.06E+07 | 9.03E+06 | 1.98E+07 | 1.00 : 2.19 |  |
| **Brian peptide** | **P85829.1** | MVPVPVHHMADEL | 60 | 6.94E+05 | 7.00E+05 | 6.87E+05 | 2.39E+06 | 2.46E+06 | 2.57E+06 | 6.94E+05 | 2.47E+06 | 1.00 : 3.57 |  |
| **Diuretic hormone (DH)** | **P85830.1** | GLDLGLSRGFSGSQAAKHLMa | 24.42 | 4.01E+08 | 4.26E+08 | 4.21E+08 | 8.49E+07 | 8.43E+07 | 8.29E+07 | 4.16E+08 | 8.40E+07 | 1.00 : 0.2 | Amidation |
| **Allatostatin (AST)** | **Q06601.1** | GRDYSFGLa | 53.26 | 8.32E+07 | 8.35E+07 | 8.56E+07 | 2.19E+08 | 2.46E+08 | 2.26E+08 | 8.41E+07 | 2.30E+08 | 1.00 : 2.74 | Amidation |
| **Apidaecins** | **Q06602.1** | GNNRPVYIPQPRPPHPRL | 35.66 | 6.18E+09 | 6.04E+09 | 5.92E+09 | 3.37E+09 | 3.60E+09 | 3.47E+09 | 6.05E+09 | 3.48E+09 | 1.00 : 0.58 |  |
| **Corazonin (CRZ)** | **Q5DW47.1** | pQTFTYSHGWTNa | 33.25 | 2.52E+06 | 2.65E+06 | 2.75E+06 | 7.79E+06 | 7.96E+06 | 8.01E+06 | 2.64E+06 | 7.92E+06 | 1.00 : 3 | Pyro-glu from Q; Amidation |
| **Tachykinins (TK)** | **Q868G6.1** | ALMGFQGVRa | 60 | 3.37E+08 | 3.66E+08 | 3.58E+08 | 1.47E+09 | 1.34E+09 | 1.32E+09 | 3.54E+08 | 1.38E+09 | 1.00 : 3.89 | Amidation |
|  |  | APMGFQGMRa | 60 | 3.60E+08 | 3.69E+08 | 3.80E+08 | 1.32E+09 | 1.24E+09 | 1.43E+09 | 3.70E+08 | 1.33E+09 | 1.00 : 3.6 | Amidation |
|  |  | SPFRYLGARa | 60 | 3.50E+07 | 3.75E+07 | 3.67E+07 | 1.21E+08 | 1.24E+08 | 1.15E+08 | 3.64E+07 | 1.20E+08 | 1.00 : 3.3 | Amidation |
|  |  | ARMGFHGMRG | 60 | 5.23E+06 | 5.02E+06 | 5.11E+06 | 1.19E+07 | 1.23E+07 | 1.11E+07 | 5.12E+06 | 1.18E+07 | 1.00 : 2.3 |  |
|  |  | GVMDFQIGLQ | 60 | 5.17E+07 | 5.23E+07 | 5.19E+07 | 1.11E+08 | 1.28E+08 | 1.12E+08 | 5.20E+07 | 1.17E+08 | 1.00 : 2.25 |  |
|  |  | ALMGFQGVRG | 26.66 | 8.62E+05 | 8.72E+05 | 8.56E+05 | 1.87E+06 | 1.75E+06 | 1.69E+06 | 8.63E+05 | 1.77E+06 | 1.00 : 2.05 |  |
|  |  | IILDALEELD | 60 | 7.62E+06 | 7.96E+06 | 7.36E+06 | 4.66E+06 | 4.48E+06 | 4.39E+06 | 7.65E+06 | 4.51E+06 | 1.00 : 0.59 |  |
|  |  | SPFRYLGA | 60 | 3.01E+07 | 3.26E+07 | 3.10E+07 | 8.63E+06 | 8.72E+06 | 8.88E+06 | 3.12E+07 | 8.74E+06 | 1.00 : 0.28 |  |
| **Neuropeptide like-1 (NPL1)** | **XP_006559359.1** | YVASLARTGDLPIRa | 30.62 | 2.05E+07 | 2.18E+07 | 2.15E+07 | 3.39E+07 | 3.47E+07 | 3.55E+07 | 2.13E+07 | 3.47E+07 | 1.00 : 1.63 | Amidation |
|  |  | NVASLARTYTLPQNAa | 60 | 4.25E+07 | 4.23E+07 | 4.26E+07 | 2.11E+08 | 2.09E+08 | 2.12E+08 | 4.25E+07 | 2.11E+08 | 1.00 : 4.96 | Amidation |
| **Pigment-dispersing hormone (PDH)** | **XP_006570344.1** | LINSLLGLPKNMNNAa | 60 | 1.35E+07 | 1.49E+07 | 1.58E+07 | 2.85E+07 | 2.66E+07 | 2.98E+07 | 1.47E+07 | 2.83E+07 | 1.00 : 1.92 | Amidation |
|  |  |  |  |  |  |  |  |  |  |  |  |  |  |
|  |  |  |  |  |  |  |  |  |  |  |  |  |  |
| **Protein** | **Protein Accession** | **Peptide** | **Significance** | **NBs 1** | **NBs 2** | **NBs 3** | **NFs 1** | **NFs 2** | **NFs 3** | **NBs** | **NFs** | **Group Profile (Ratio)** | **PTM** |
| **Apidaecins** | **Q06602.1** | GNNRPVYIPQPRPPHPRL | 32.1 | 6.18E+09 | 6.04E+09 | 5.92E+09 | 3.15E+09 | 3.36E+09 | 3.31E+09 | 6.05E+09 | 3.27E+09 | 1.00 : 0.54 |  |
|  |  | VYIPQPRPPHPRL | 60 | 1.33E+09 | 1.30E+09 | 1.23E+09 | 2.84E+08 | 2.70E+08 | 2.74E+08 | 1.29E+09 | 2.76E+08 | 1.00 : 0.21 |  |
| **Corazonin (CRZ)** | **Q5DW47.1** | pQTFTYSHGWTNa | 53.03 | 2.52E+06 | 2.65E+06 | 2.75E+06 | 6.36E+06 | 6.55E+06 | 6.30E+06 | 2.64E+06 | 6.40E+06 | 1.00 : 2.43 | Pyro-glu from Q; Amidation |
| **Diuretic hormone (DH)** | **P85830.1** | GLDLGLSRGFSGSQAAKHLMa | 60 | 4.01E+08 | 4.26E+08 | 4.21E+08 | 1.41E+08 | 1.25E+08 | 1.39E+08 | 4.16E+08 | 1.35E+08 | 1.00 : 0.33 | Amidation |
| **FMRFamide** | **ACI90290.1** | GRNDLNFIRYa | 43.64 | 2.94E+06 | 3.09E+06 | 3.11E+06 | 4.86E+06 | 4.79E+06 | 4.65E+06 | 3.05E+06 | 4.68E+06 | 1.00 : 1.56 | Amidation |
|  |  | TWKSPDIVIRFa | 60 | 1.81E+07 | 1.85E+07 | 1.69E+07 | 7.71E+06 | 7.80E+06 | 7.68E+06 | 1.78E+07 | 7.73E+06 | 1.00 : 0.43 | Amidation |
| **Myosuppressin** | **P85527.1** | pQDVDHVFLRFa | 36.72 | 1.33E+08 | 1.21E+08 | 1.28E+08 | 4.23E+08 | 4.37E+08 | 4.36E+08 | 1.27E+08 | 4.32E+08 | 1.00 : 3.39 | Pyro-glu from Q; Amidation |
|  |  | pQDVDHVFLR | 60 | 1.23E+07 | 1.33E+07 | 1.39E+07 | 4.15E+07 | 4.38E+07 | 4.21E+07 | 1.32E+07 | 4.25E+07 | 1.00 : 3.23 | Pyro-glu from Q |
| **Neuropeptide like-1 (NPL1)** | **XP_006559359.1** | YVASLARTGDLPIRa | 60 | 2.05E+07 | 2.18E+07 | 2.15E+07 | 4.49E+07 | 4.57E+07 | 4.39E+07 | 2.13E+07 | 4.48E+07 | 1.00 : 2.11 | Amidation |
|  |  | NVASLARTYTLPQNAa | 60 | 4.25E+07 | 4.23E+07 | 4.26E+07 | 8.16E+07 | 8.31E+07 | 8.37E+07 | 4.25E+07 | 8.28E+07 | 1.00 : 1.95 | Amidation |
| **PBAN-type neuropeptides (PBAN)** | **A8CL69.1** | TSQDITSGMWFGPRLa | 30.19 | 6.88E+08 | 6.55E+08 | 6.65E+08 | 1.04E+09 | 1.02E+09 | 9.98E+08 | 6.69E+08 | 1.02E+09 | 1.00 : 1.52 | Amidation |
|  |  | MWFGPRLa | 60 | 2.04E+06 | 2.04E+06 | 2.15E+06 | 7.56E+05 | 7.59E+05 | 7.49E+05 | 2.08E+06 | 7.55E+05 | 1.00 : 0.36 | Amidation |
| **Pigment-dispersing hormone (PDH)** | **XP_006570344.1** | LINSLLGLPKNMNNAa | 60 | 1.35E+07 | 1.49E+07 | 1.58E+07 | 2.78E+07 | 2.85E+07 | 2.66E+07 | 1.47E+07 | 2.76E+07 | 1.00 : 1.88 | Amidation |
| **Prohormone-3** | **P85828.1** | ITGQGNRIF | 60 | 8.78E+06 | 8.66E+06 | 8.67E+06 | 4.36E+07 | 4.27E+07 | 4.25E+07 | 8.70E+06 | 4.29E+07 | 1.00 : 4.93 |  |
|  |  | SLKAPFA | 38.97 | 9.06E+06 | 9.13E+06 | 8.90E+06 | 1.71E+07 | 1.68E+07 | 1.75E+07 | 9.03E+06 | 1.71E+07 | 1.00 : 1.9 |  |
| **Tachykinins (TK)** | **Q868G6.1** | ALMGFQGVRG | 60 | 8.62E+05 | 8.72E+05 | 8.56E+05 | 4.27E+06 | 4.33E+06 | 4.47E+06 | 8.63E+05 | 4.36E+06 | 1.00 : 5.05 |  |
|  |  | ALMGFQGVRa | 30.19 | 3.37E+08 | 3.66E+08 | 3.58E+08 | 3.57E+09 | 3.63E+09 | 3.39E+09 | 3.54E+08 | 3.53E+09 | 1.00 : 9.98 | Amidation |
|  |  | APMGFQGMRa | 60 | 3.60E+08 | 3.69E+08 | 3.80E+08 | 3.38E+09 | 3.27E+09 | 3.59E+09 | 3.70E+08 | 3.41E+09 | 1.00 : 9.23 | Amidation |
|  |  | ARMGFHGMRG | 60 | 5.23E+06 | 5.02E+06 | 5.11E+06 | 1.26E+07 | 1.33E+07 | 1.21E+07 | 5.12E+06 | 1.28E+07 | 1.00 : 2.49 |  |
|  |  | IILDALEELD | 41.85 | 7.62E+06 | 7.96E+06 | 7.36E+06 | 2.13E+06 | 2.22E+06 | 2.30E+06 | 7.65E+06 | 2.22E+06 | 1.00 : 0.29 |  |
|  |  | SPFRYLGA | 31.06 | 3.01E+07 | 3.26E+07 | 3.10E+07 | 7.52E+06 | 7.72E+06 | 7.69E+06 | 3.12E+07 | 7.64E+06 | 1.00 : 0.24 |  |
|  |  |  |  |  |  |  |  |  |  |  |  |  |  |
|  |  |  |  |  |  |  |  |  |  |  |  |  |  |
| **Protein** | **Protein Accession** | **Peptide** | **Significance** | **PFs 1** | **PFs 2** | **PFs 3** | **NFs 1** | **NFs 2** | **NFs 3** | **PFs** | **NFs** | **Group Profile (Ratio)** | **PTM** |
| **Allatostatin (AST)** | **Q06601.1** | AVHYSGGQPLGSKRPNDMLSQRYHFGLa | 30.34 | 4.90E+08 | 4.69E+08 | 4.98E+08 | 3.18E+08 | 3.25E+08 | 3.17E+08 | 4.86E+08 | 3.20E+08 | 1.00 : 0.66 | Amidation |
|  |  | WIDTNDNKRGRDYSFGLa | 60 | 4.38E+07 | 4.15E+07 | 4.29E+07 | 2.24E+07 | 2.52E+07 | 2.38E+07 | 4.27E+07 | 2.38E+07 | 1.00 : 0.56 | Amidation |
| **Brian peptide** | **P85829.1** | MVPVPVHHMADELLRNGPDTVI | 60 | 9.95E+08 | 9.90E+08 | 1.04E+09 | 1.89E+09 | 1.98E+09 | 1.77E+09 | 1.01E+09 | 1.88E+09 | 1.00 : 1.86 |  |
| **CAPA peptides-like** | **XP_006559865.1** | AFGLLTYPRIa | 60 | 2.88E+07 | 2.78E+07 | 2.63E+07 | 4.99E+07 | 4.66E+07 | 4.94E+07 | 2.76E+07 | 4.86E+07 | 1.00 : 1.76 | Amidation |
| **Diuretic hormone (DH)** | **P85830.1** | GLDLGLSRGFSGSQAAKHLMa | 60 | 8.49E+07 | 8.43E+07 | 8.29E+07 | 1.41E+08 | 1.25E+08 | 1.39E+08 | 8.41E+07 | 1.35E+08 | 1.00 : 1.61 | Amidation |
| **FMRFamide** | **ACI90290.1** | TWKSPDIVIRFa | 60 | 4.27E+07 | 4.30E+07 | 4.18E+07 | 7.71E+06 | 7.80E+06 | 7.68E+06 | 4.25E+07 | 7.73E+06 | 1.00 : 0.18 | Amidation |
| **Neuropeptide like-1 (NPL1)** | **XP_006559359.1** | SVSSLARTGDLPVREQ | 35.02 | 4.01E+07 | 4.21E+07 | 4.11E+07 | 2.52E+07 | 2.33E+07 | 2.38E+07 | 4.11E+07 | 2.41E+07 | 1.00 : 0.59 |  |
|  |  | NIASLMRDYDQSRENRVPFPa | 60 | 3.00E+08 | 2.86E+08 | 2.98E+08 | 1.42E+08 | 1.47E+08 | 1.64E+08 | 2.95E+08 | 1.51E+08 | 1.00 : 0.51 | Amidation |
|  |  | YVASLARTGDLPIRGQ | 27 | 2.75E+08 | 2.92E+08 | 2.87E+08 | 6.32E+07 | 6.44E+07 | 6.27E+07 | 2.85E+08 | 6.34E+07 | 1.00 : 0.22 |  |
| **PBAN-type neuropeptides (PBAN)** | **A8CL69.1** | QITQFTPRLa | 60 | 4.23E+07 | 4.34E+07 | 4.42E+07 | 2.05E+07 | 2.24E+07 | 2.19E+07 | 4.33E+07 | 2.16E+07 | 1.00 : 0.5 | Amidation |
|  |  | pQITQFTPRLa | 33.65 | 3.50E+08 | 3.36E+08 | 3.38E+08 | 8.50E+07 | 8.36E+07 | 8.38E+07 | 3.41E+08 | 8.41E+07 | 1.00 : 0.25 | Pyro-glu from Q; Amidation |
| **Prohormone-1** | **P85798.1** | LRNQLDIGDLQ | 42.97 | 9.56E+08 | 9.48E+08 | 9.34E+08 | 4.52E+09 | 4.41E+09 | 4.45E+09 | 9.46E+08 | 4.46E+09 | 1.00 : 4.71 |  |
| **Prohormone-4** | **P85831.1** | IDLSRFYGHFNT | 60 | 6.46E+08 | 6.50E+08 | 6.36E+08 | 3.46E+09 | 3.50E+09 | 3.36E+09 | 6.44E+08 | 3.44E+09 | 1.00 : 5.34 |  |
|  |  | IDLSRFYGHFN | 34.77 | 1.76E+08 | 1.64E+08 | 1.54E+08 | 3.77E+08 | 3.96E+08 | 3.66E+08 | 1.65E+08 | 3.80E+08 | 1.00 : 2.31 |  |
| **Short neuropeptide F (sNPF)** | **XP_006565207.1** | SDPHLSILS | 33.58 | 1.93E+06 | 1.84E+06 | 1.93E+06 | 9.67E+05 | 9.50E+05 | 9.57E+05 | 1.90E+06 | 9.58E+05 | 1.00 : 0.5 |  |
|  |  | SPSLRLRFa | 42.51 | 6.44E+06 | 6.16E+06 | 6.37E+06 | 1.11E+06 | 1.12E+06 | 1.33E+06 | 6.32E+06 | 1.19E+06 | 1.00 : 0.19 | Amidation |
| **Tachykinins (TK)** | **Q868G6.1** | APMGFQGMRG | 60 | 5.44E+07 | 5.60E+07 | 5.56E+07 | 2.38E+08 | 2.47E+08 | 2.43E+08 | 5.53E+07 | 2.43E+08 | 1.00 : 4.39 |  |
|  |  | APMGFQGMRa | 59.71 | 1.32E+09 | 1.24E+09 | 1.43E+09 | 3.38E+09 | 3.27E+09 | 3.59E+09 | 1.33E+09 | 3.41E+09 | 1.00 : 2.57 | Amidation |
|  |  | ALMGFQGVRa | 60 | 1.47E+09 | 1.34E+09 | 1.32E+09 | 3.57E+09 | 3.63E+09 | 3.39E+09 | 1.38E+09 | 3.53E+09 | 1.00 : 2.56 | Amidation |
