# Supplementary material for: Tachykinin signaling inhibits task-specific behavioral responsiveness in honeybee workers
Source: eLife. 2021 Mar 24;10:e64830. doi: 10.7554/eLife.64830 (PMC8016481; doi:10.7554/eLife.64830)
Supplement: Figure 2—source data 2. [file elife-64830-fig2-data2.docx]

Quantitative neuropeptide comparison of different behavioral phenotypes of *Apis cerana cerana* workers. (manuscript section 2.2)

"**Protein Accession**": the unique number given to mark the entry of a protein in the database NCBInr. "**Peptide**": the amino acid sequence of the peptide. "**Significance (-10lgP)**": the peptide confidence score. "**NBs**": nurse bees. "**PFs**": pollen foragers. "**NFs**": nectar foragers. "**Group Profile (Ratio)**": the relative abundance ratio to the base group. "**PTM**": the post translational modification types present in the peptide.

| **Protein** | **Protein Accession** | **Peptide** | **Significance** | **NBs 1** | **NBs 2** | **NBs 3** | **PFs 1** | **PFs 2** | **PFs 3** | **NBs** | **PFs** | **Group Profile (Ratio)** | **PTM** |
| --- | --- | --- | --- | --- | --- | --- | --- | --- | --- | --- | --- | --- | --- |
| **Prohormone-3** | PBC27982.1 | SLKAPFA | 60 | 5.93E+07 | 5.68E+07 | 5.76E+07 | 1.40E+08 | 1.46E+08 | 1.44E+08 | 5.79E+07 | 1.43E+08 | 1.00 : 2.48 |  |
|  |  | ITGQGNRIF | 60 | 2.20E+07 | 2.39E+07 | 2.38E+07 | 6.70E+07 | 6.54E+07 | 6.88E+07 | 2.32E+07 | 6.71E+07 | 1.00 : 2.89 |  |
| **Apidaecins** | PBC28057.1 | GNNRPVYIPQPRPPHPRL | 60 | 4.98E+09 | 4.86E+09 | 5.07E+09 | 2.50E+09 | 2.59E+09 | 2.58E+09 | 4.97E+09 | 2.56E+09 | 1.00 : 0.51 |  |
| **Diuretic hormone (DH)** | PBC28214.1 | GLDLGLSRGFSGSQAAKHLMa | 39.87 | 4.60E+08 | 4.57E+08 | 4.22E+08 | 1.79E+09 | 1.91E+09 | 1.90E+09 | 4.46E+08 | 1.87E+09 | 1.00 : 4.18 | Amidation |
| **Short neuropeptide F (sNPF)** | PBC30406.1 | SDPHLSIGILSKPISAIPSSKFDD | 60 | 3.73E+08 | 3.94E+08 | 3.79E+08 | 1.46E+08 | 1.60E+08 | 1.57E+08 | 3.82E+08 | 1.54E+08 | 1.00 : 0.4 |  |
| **Corazonin (CRZ)** | PBC31004.1 | pQMFTYSHGWTNa | 28.91 | 9.94E+07 | 9.45E+07 | 9.63E+07 | 3.79E+08 | 3.73E+08 | 3.71E+08 | 9.67E+07 | 3.74E+08 | 1.00 : 3.87 | Pyro-glu from Q; Amidation |
| **SIFamide** | PBC31251.1 | KPPFNGSIFa | 60 | 1.97E+08 | 1.84E+08 | 1.81E+08 | 8.11E+07 | 9.40E+07 | 9.48E+07 | 1.87E+08 | 9.00E+07 | 1.00 : 0.48 | Amidation |
|  |  | AYRKPPFNGSIFa | 60 | 1.68E+09 | 1.55E+09 | 1.64E+09 | 4.56E+08 | 4.76E+08 | 4.75E+08 | 1.62E+09 | 4.69E+08 | 1.00 : 0.29 | Amidation |
| **Tachykinins (TK)** | PBC31431.1 | ASFDDEYY | 56.6 | 6.50E+06 | 6.08E+06 | 6.11E+06 | 3.49E+07 | 3.10E+07 | 3.38E+07 | 6.23E+06 | 3.32E+07 | 1.00 : 5.33 |  |
|  |  | APMGFQGMRa | 60 | 9.27E+08 | 9.24E+08 | 9.17E+08 | 4.58E+09 | 4.73E+09 | 4.55E+09 | 9.23E+08 | 4.62E+09 | 1.00 : 5.01 | Amidation |
|  |  | APMGFYGTRG | 60 | 7.36E+06 | 7.35E+06 | 7.19E+06 | 3.19E+07 | 3.83E+07 | 3.85E+07 | 7.30E+06 | 3.62E+07 | 1.00 : 4.96 |  |
|  |  | APMGFQGMRG | 40.07 | 9.10E+06 | 9.29E+06 | 9.11E+06 | 4.22E+07 | 4.19E+07 | 4.36E+07 | 9.17E+06 | 4.26E+07 | 1.00 : 4.64 |  |
|  |  | ALMGFQGVRa | 60 | 8.14E+08 | 8.19E+08 | 8.28E+08 | 3.87E+09 | 3.66E+09 | 3.89E+09 | 8.20E+08 | 3.81E+09 | 1.00 : 4.64 | Amidation |
|  |  | APVGYQEMQGKKNSASLNSENFGIF | 55.82 | 4.61E+07 | 4.43E+07 | 4.48E+07 | 1.85E+08 | 1.83E+08 | 1.82E+08 | 4.51E+07 | 1.83E+08 | 1.00 : 4.07 |  |
|  |  | ARMGFHGMRG | 41.94 | 1.29E+07 | 1.40E+07 | 1.42E+07 | 4.16E+07 | 4.24E+07 | 4.17E+07 | 1.37E+07 | 4.19E+07 | 1.00 : 3.06 |  |
|  |  | SPFRYLGV | 60 | 5.53E+07 | 5.86E+07 | 5.75E+07 | 1.45E+08 | 1.64E+08 | 1.61E+08 | 5.71E+07 | 1.57E+08 | 1.00 : 2.74 |  |
|  |  | ALMGFQGVRG | 37.82 | 1.98E+06 | 2.09E+06 | 1.92E+06 | 3.64E+06 | 3.64E+06 | 3.91E+06 | 2.00E+06 | 3.73E+06 | 1.00 : 1.87 |  |
| **Prohormone-2** | PBC32727.1 | NVPIYQEPRF | 46.37 | 9.22E+08 | 9.28E+08 | 9.43E+08 | 3.25E+08 | 3.72E+08 | 3.88E+08 | 9.31E+08 | 3.62E+08 | 1.00 : 0.39 |  |
|  |  | LPTNLGEDTKKTEQTMRPKS | 60 | 5.12E+08 | 5.16E+08 | 5.02E+08 | 1.44E+08 | 1.56E+08 | 1.47E+08 | 5.10E+08 | 1.49E+08 | 1.00 : 0.29 |  |
|  |  | VPIYQEPRF | 33.21 | 9.74E+07 | 9.61E+07 | 9.48E+07 | 2.38E+07 | 2.16E+07 | 2.06E+07 | 9.61E+07 | 2.20E+07 | 1.00 : 0.23 |  |
| **Neuropeptide like-1 (NPL1)** | PBC32914.1 | SISSLARTGDLPVREQ | 30.75 | 3.69E+08 | 3.39E+08 | 3.46E+08 | 1.38E+09 | 1.33E+09 | 1.46E+09 | 3.51E+08 | 1.39E+09 | 1.00 : 3.96 |  |
|  |  | NVGSVAREHGLPYa | 60 | 6.26E+08 | 6.78E+08 | 6.89E+08 | 2.21E+09 | 2.73E+09 | 2.22E+09 | 6.64E+08 | 2.39E+09 | 1.00 : 3.59 | Amidation |
|  |  | NVGTLARDFALPPa | 36.07 | 5.22E+07 | 5.05E+07 | 5.14E+07 | 1.31E+08 | 1.52E+08 | 1.21E+08 | 5.14E+07 | 1.35E+08 | 1.00 : 2.62 | Amidation |
| **Pigment-dispersing hormone (PDH)** | PBC32545.1 | NSELINSLLGLPKNMNNAa | 23.88 | 7.62E+07 | 7.93E+07 | 7.72E+07 | 2.76E+08 | 2.75E+08 | 2.46E+08 | 7.76E+07 | 2.66E+08 | 1.00 : 3.43 | Amidation |
| **PBAN-type neuropeptides (PBAN)** | PBC32274.1 | pQITQFTPRLa | 33.45 | 2.79E+07 | 2.85E+07 | 2.58E+07 | 1.59E+08 | 1.71E+08 | 1.55E+08 | 2.74E+07 | 1.62E+08 | 1.00 : 5.9 | Pyro-glu from Q; Amidation |
| **Orcokinin (ORC)** | XP_016908608.1 | NLDEIDRVGWSGFV | 42.33 | 2.22E+08 | 2.48E+08 | 2.52E+08 | 6.87E+08 | 6.87E+08 | 6.53E+08 | 2.41E+08 | 6.76E+08 | 1.00 : 2.81 |  |
| **Prohormone-4** | PBC32608.1 | IDLSRFYGHFNT | 30.72 | 9.52E+08 | 9.58E+08 | 9.13E+08 | 3.06E+09 | 3.12E+09 | 3.03E+09 | 9.41E+08 | 3.07E+09 | 1.00 : 3.26 |  |
|  |  |  |  |  |  |  |  |  |  |  |  |  |  |
|  |  |  |  |  |  |  |  |  |  |  |  |  |  |
| **Protein** | **Protein Accession** | **Peptide** | **Significance** | **NBs 1** | **NBs 2** | **NBs 3** | **NFs 1** | **NFs 2** | **NFs 3** | **NBs** | **NFs** | **Group Profile (Ratio)** | **PTM** |
| **Prohormone-3** | PBC27982.1 | SLKAPFA | 60 | 5.93E+07 | 5.68E+07 | 5.76E+07 | 1.75E+08 | 1.62E+08 | 1.77E+08 | 5.79E+07 | 1.71E+08 | 1.00 ：2.96 |  |
| **Apidaecins** | PBC28057.1 | GNNRPVYIPQPRPPHPRL | 35.67 | 4.98E+09 | 4.86E+09 | 5.07E+09 | 1.89E+09 | 1.92E+09 | 1.98E+09 | 4.97E+09 | 1.93E+09 | 1.00 ：0.39 |  |
| **Diuretic hormone (DH)** | PBC28214.1 | GLDLGLSRGFSGSQAAKHLMa | 60 | 4.60E+08 | 4.57E+08 | 4.22E+08 | 2.39E+09 | 2.40E+09 | 2.51E+09 | 4.46E+08 | 2.43E+09 | 1.00 ：5.45 | Amidation |
| **Short neuropeptide F (sNPF)** | PBC30406.1 | SPSLRLRFa | 60 | 3.86E+07 | 3.68E+07 | 3.63E+07 | 5.84E+06 | 5.43E+06 | 5.37E+06 | 3.72E+07 | 5.55E+06 | 1.00 ：0.15 | Amidation |
| **Corazonin (CRZ)** | PBC31004.1 | pQMFTYSHGWTNa | 48.07 | 9.94E+07 | 9.45E+07 | 9.63E+07 | 4.49E+08 | 4.58E+08 | 4.78E+08 | 9.67E+07 | 4.62E+08 | 1.00 ：4.77 | Amidation |
| **SIFamide** | PBC31251.1 | KPPFNGSIFa | 60 | 1.97E+08 | 1.84E+08 | 1.81E+08 | 6.87E+07 | 6.79E+07 | 6.70E+07 | 1.87E+08 | 6.79E+07 | 1.00 ：0.36 | Amidation |
|  |  | AYRKPPFNGSIFa | 60 | 1.68E+09 | 1.55E+09 | 1.64E+09 | 3.26E+08 | 3.14E+08 | 3.06E+08 | 1.62E+09 | 3.15E+08 | 1.00 ：0.19 | Amidation |
| **Tachykinins (TK)** | PBC31431.1 | ALMGFQGVRa | 60 | 8.14E+08 | 8.19E+08 | 8.28E+08 | 9.80E+09 | 9.58E+09 | 9.57E+09 | 8.20E+08 | 9.65E+09 | 1.00 : 11.76 | Amidation |
|  |  | APMGFQGMRa | 60 | 9.27E+08 | 9.24E+08 | 9.17E+08 | 9.93E+09 | 1.02E+10 | 9.93E+09 | 9.23E+08 | 1.00E+10 | 1.00 ：10.86 | Amidation |
|  |  | APMGFQGMRG | 56.21 | 9.10E+06 | 9.29E+06 | 9.11E+06 | 8.10E+07 | 7.91E+07 | 7.86E+07 | 9.17E+06 | 7.96E+07 | 1.00 ：8.68 |  |
|  |  | ASFDDEYY | 39.72 | 6.50E+06 | 6.08E+06 | 6.11E+06 | 4.46E+07 | 4.32E+07 | 4.21E+07 | 6.23E+06 | 4.33E+07 | 1.00 ：6.95 |  |
|  |  | APMGFYGTRG | 60 | 7.36E+06 | 7.35E+06 | 7.19E+06 | 4.62E+07 | 4.58E+07 | 4.99E+07 | 7.30E+06 | 4.73E+07 | 1.00 ：6.48 |  |
|  |  | ALMGFQGVRG | 60 | 1.98E+06 | 2.09E+06 | 1.92E+06 | 1.07E+07 | 1.21E+07 | 1.23E+07 | 2.00E+06 | 1.17E+07 | 1.00 ：5.86 |  |
|  |  | APVGYQEMQGKKNSASLNSENFGIF | 35.68 | 4.61E+07 | 4.43E+07 | 4.48E+07 | 1.86E+08 | 1.82E+08 | 1.92E+08 | 4.51E+07 | 1.87E+08 | 1.00 ：4.14 |  |
|  |  | ARMGFHGMRG | 28.15 | 1.29E+07 | 1.40E+07 | 1.42E+07 | 5.47E+07 | 5.44E+07 | 5.44E+07 | 1.37E+07 | 5.45E+07 | 1.00 ：3.98 |  |
| **Prohormone-2** | PBC32727.1 | VPIYQEPRF | 25.35 | 9.74E+07 | 9.51E+07 | 9.48E+07 | 3.12E+07 | 3.29E+07 | 3.30E+07 | 9.58E+07 | 3.24E+07 | 1.00 ：0.34 |  |
|  |  | NVPIYQEPRF | 60 | 9.22E+08 | 9.28E+08 | 9.43E+08 | 2.88E+08 | 3.15E+08 | 3.00E+08 | 9.31E+08 | 3.01E+08 | 1.00 ：0.32 |  |
| **Neuropeptide like-1 (NPL1)** | PBC32914.1 | YVASLARTGDLPIRa | 60 | 6.26E+08 | 6.78E+08 | 6.89E+08 | 3.17E+09 | 3.41E+09 | 3.20E+09 | 6.64E+08 | 3.26E+09 | 1.00 ：4.91 | Amidation |
|  |  | NVGSVAREHGLPYa | 36.43 | 3.69E+08 | 3.39E+08 | 3.46E+08 | 1.45E+09 | 1.40E+09 | 1.50E+09 | 3.51E+08 | 1.45E+09 | 1.00 ：4.13 | Amidation |
|  |  | SISSLARTGDLPVREQ | 60 | 5.22E+07 | 5.05E+07 | 5.14E+07 | 1.53E+08 | 1.55E+08 | 1.42E+08 | 5.14E+07 | 1.50E+08 | 1.00 ：2.92 |  |
| **Pigment-dispersing hormone (PDH)** | PBC32545.1 | NSELINSLLGLPKNMNNAa | 60 | 7.62E+07 | 7.93E+07 | 7.72E+07 | 3.02E+08 | 3.19E+08 | 3.18E+08 | 7.76E+07 | 3.13E+08 | 1.00 ：4.04 | Amidation |
| **PBAN-type neuropeptides (PBAN)** | PBC32274.1 | TSQDITSGMWFGPRLa | 36.94 | 8.63E+07 | 8.83E+07 | 8.45E+07 | 2.38E+08 | 2.50E+08 | 2.47E+08 | 8.64E+07 | 2.45E+08 | 1.00 ：2.84 | Amidation |
| **Orcokinin (ORC)** | XP_016908608.1 | LTNYLATGHRTNGGPVI | 43.31 | 4.29E+08 | 4.11E+08 | 4.24E+08 | 2.17E+09 | 2.13E+09 | 2.19E+09 | 4.21E+08 | 2.16E+09 | 1.00 ：5.13 |  |
|  |  | NLDEIDRVGWSGFV | 60 | 2.22E+08 | 2.48E+08 | 2.52E+08 | 9.30E+08 | 9.49E+08 | 9.42E+08 | 2.41E+08 | 9.40E+08 | 1.00 ：3.91 |  |
| **Prohormone-4** | PBC32608.1 | IDLSRFYGHFNT | 25.79 | 9.52E+08 | 9.58E+08 | 9.13E+08 | 6.23E+09 | 6.19E+09 | 6.34E+09 | 9.41E+08 | 6.25E+09 | 1.00 ：6.65 |  |
|  |  |  |  |  |  |  |  |  |  |  |  |  |  |
|  |  |  |  |  |  |  |  |  |  |  |  |  |  |
|  |  |  |  |  |  |  |  |  |  |  |  |  |  |
| **Protein** | **Protein Accession** | **Peptide** | **Significance** | **PFs 1** | **PFs 2** | **PFs 3** | **NFs 1** | **NFs 2** | **NFs 3** | **PFs** | **NFs** | **Group Profile (Ratio)** | **PTM** |
| **Short neuropeptide F (sNPF)** | PBC30406.1 | SPSLRLRFa | 60 | 1.37E+07 | 1.59E+07 | 1.46E+07 | 5.84E+06 | 5.43E+06 | 5.37E+06 | 1.47E+07 | 5.55E+06 | 1.00 : 0.38 | Amidation |
|  |  | SQRSPSLRLRFa | 43.9 | 4.30E+07 | 4.03E+07 | 4.33E+07 | 1.12E+07 | 1.11E+07 | 1.20E+07 | 4.22E+07 | 1.14E+07 | 1.00 : 0.27 | Amidation |
| **Tachykinins (TK)** | PBC31431.1 | ALMGFQGVRG | 51.11 | 3.64E+06 | 3.64E+06 | 3.91E+06 | 1.07E+07 | 1.21E+07 | 1.23E+07 | 3.73E+06 | 1.17E+07 | 1.00 : 3.14 |  |
|  |  | ALMGFQGVRa | 60 | 3.87E+09 | 3.66E+09 | 3.89E+09 | 9.80E+09 | 9.58E+09 | 9.57E+09 | 3.81E+09 | 9.65E+09 | 1.00 : 2.54 | Amidation |
|  |  | APMGFQGMRa | 60 | 4.58E+09 | 4.63E+09 | 4.55E+09 | 9.93E+09 | 1.02E+10 | 9.93E+09 | 4.59E+09 | 1.00E+10 | 1.00 : 2.18 | Amidation |
|  |  | APMGFQGMRG | 47.56 | 4.22E+07 | 4.19E+07 | 4.36E+07 | 8.10E+07 | 7.91E+07 | 7.86E+07 | 4.26E+07 | 7.96E+07 | 1.00 : 1.87 |  |
| **PBAN-type neuropeptides (PBAN)** | PBC32274.1 | pQLHNIIDKPRQNFNDPRF | 60 | 6.45E+07 | 6.61E+07 | 6.72E+07 | 1.56E+07 | 1.61E+07 | 1.74E+07 | 6.59E+07 | 1.64E+07 | 1.00 : 0.25 | Pyro-glu from Q |
|  |  | pQITQFTPRLa | 26.86 | 1.59E+08 | 1.71E+08 | 1.55E+08 | 4.20E+07 | 4.55E+07 | 4.41E+07 | 1.62E+08 | 4.39E+07 | 1.00 : 0.27 | Pyro-glu from Q; Amidation |
|  |  | pQLHNIIDKPRQNFNDP | 34.07 | 6.24E+06 | 6.13E+06 | 6.39E+06 | 2.29E+06 | 2.13E+06 | 2.01E+06 | 6.25E+06 | 2.14E+06 | 1.00 : 0.34 | Pyro-glu from Q |
| **Prohormone-4** | PBC32608.1 | IDLSRFYGHFN | 49.52 | 2.50E+09 | 2.51E+09 | 2.49E+09 | 5.28E+09 | 5.29E+09 | 5.21E+09 | 2.50E+09 | 5.26E+09 | 1.00 : 2.1 |  |
|  |  | IDLSRFYGHFNT | 60 | 3.06E+09 | 3.12E+09 | 3.03E+09 | 6.23E+09 | 6.19E+09 | 6.34E+09 | 3.07E+09 | 6.25E+09 | 1.00 : 2.04 |  |
| **Neuropeptide like-1 (NPL1)** | PBC32914.1 | SISSLARTGDLPVREQ | 56.01 | 1.38E+09 | 1.33E+09 | 1.46E+09 | 1.53E+08 | 1.55E+08 | 1.42E+08 | 1.39E+09 | 1.50E+08 | 1.00 : 0.11 |  |
|  |  | NIASLIRDYDQSRENRVSFPa | 39.6 | 1.40E+08 | 1.59E+08 | 1.34E+08 | 2.99E+08 | 2.86E+08 | 3.03E+08 | 1.44E+08 | 2.96E+08 | 1.00 : 2.05 | Amidation |
|  |  | YVASLARTGDLPIRGQ | 30.32 | 3.13E+08 | 3.00E+08 | 3.05E+08 | 1.50E+08 | 1.56E+08 | 1.58E+08 | 3.06E+08 | 1.55E+08 | 1.00 : 0.5 |  |
| **Allatostatin (AST)** | PBC34787.1 | AVHYSGGQPLGSKRPNDMLSQRYHFGLa | 60 | 8.06E+08 | 7.84E+08 | 7.90E+08 | 5.10E+08 | 5.17E+08 | 5.00E+08 | 7.93E+08 | 5.09E+08 | 1.00 : 0.64 | Amidation |
|  |  | WIDTNDNKRGRDYSFGLa | 28.34 | 7.12E+07 | 7.11E+07 | 7.06E+07 | 4.35E+07 | 4.19E+07 | 4.22E+07 | 7.10E+07 | 4.25E+07 | 1.00 : 0.6 | Amidation |
| **Brain peptide** | XP_016908970 | MVPVPVHHMADELLRSGPDTVI | 60 | 5.20E+08 | 4.91E+08 | 4.94E+08 | 9.09E+08 | 9.84E+08 | 9.14E+08 | 5.02E+08 | 9.36E+08 | 1.00 : 1.87 |  |
| **FMRFamide** | XP_016920932.1 | TWKSPDIVIRFa | 60 | 1.94E+07 | 2.20E+07 | 2.10E+07 | 3.86E+07 | 3.92E+07 | 4.09E+07 | 2.08E+07 | 3.96E+07 | 1.00 : 1.9 | Amidation |
