# Supplementary material for: Tachykinin signaling inhibits task-specific behavioral responsiveness in honeybee workers
Source: eLife. 2021 Mar 24;10:e64830. doi: 10.7554/eLife.64830 (PMC8016481; doi:10.7554/eLife.64830)
Supplement: Figure 2—source data 3. [file elife-64830-fig2-data3.docx]

Quantitative neuropeptide comparison between *Apis cerana cerana* and *Apis mellifera ligustica*. (manuscript section 2.2)

"**Protein Accession**": the unique number given to mark the entry of a protein in the database NCBInr. "**Peptide**": the amino acid sequence of the peptide. "**Significance (-10lgP)**": the peptide confidence score. "**NBs**": nurse bees. "**PFs**": pollen foragers. "**NFs**": nectar foragers. "**Group Profile (Ratio)**": the relative abundance ratio to the base group. "**PTM**": the post translational modification types present in the peptide.

| **Protein** | **Peptide** | **Significance** | **ACC-NBs 1** | **ACC-NBs 2** | **ACC-NBs 3** | **AML-NBs 1** | **AML-NBs 2** | **AML-NBs 3** | **ACC-NBs** | **AML-NBs** | **Group Profile (Ratio)** | **PTM** |
| --- | --- | --- | --- | --- | --- | --- | --- | --- | --- | --- | --- | --- |
| **Allatostatin (AST)** | AYTYVSEYKRLPVYNFGIa | 60 | 3.41E+08 | 3.10E+08 | 3.21E+08 | 1.09E+08 | 1.07E+08 | 1.11E+08 | 3.24E+08 | 1.09E+08 | 1.00 : 0.34 | Amidation |
| **Diuretic hormone (DH)** | GLDLGLSRGFSGSQAA | 36.51 | 1.22E+06 | 1.11E+06 | 1.08E+06 | 3.24E+06 | 3.37E+06 | 3.08E+06 | 1.14E+06 | 3.23E+06 | 1.00 : 2.84 |  |
|  | GLDLGLSRGFSGSQAAKHLMa | 60 | 2.39E+08 | 2.20E+08 | 2.27E+08 | 4.01E+08 | 4.26E+08 | 4.21E+08 | 2.29E+08 | 4.16E+08 | 1.00 : 1.82 | Amidation |
| **SIFamide** | YRKPPFNGSIFa | 60 | 4.54E+07 | 4.35E+07 | 4.46E+07 | 1.22E+08 | 1.17E+08 | 1.15E+08 | 4.45E+07 | 1.18E+08 | 1.00 : 2.65 | Amidation |
|  | KPPFNGSIFa | 35.18 | 1.97E+08 | 1.84E+08 | 1.81E+08 | 3.97E+08 | 4.10E+08 | 3.83E+08 | 1.87E+08 | 3.97E+08 | 1.00 : 2.12 | Amidation |
| **Myosuppressin** | pQDVDHVFLRFa | 49.38 | 6.80E+07 | 6.50E+07 | 6.67E+07 | 1.33E+08 | 1.21E+08 | 1.28E+08 | 6.66E+07 | 1.27E+08 | 1.00 : 1.91 | Pyro-glu from Q; Amidation |
| **PBAN-type neuropeptide (PBAN)** | GMWFGPRLa | 60 | 7.02E+06 | 6.96E+06 | 6.87E+06 | 1.53E+07 | 1.37E+07 | 1.43E+07 | 6.95E+06 | 1.44E+07 | 1.00 : 2.08 | Amidation |
| **Prohormone-2** | SQAYDPYSNAAQFQLSSQSRGYPYQHRL | 60 | 4.18E+07 | 4.36E+07 | 4.23E+07 | 1.24E+08 | 1.19E+08 | 1.08E+08 | 4.26E+07 | 1.17E+08 | 1.00 : 2.75 |  |
| **Prohormone-4** | IDLSRFYGHF | 41.13 | 2.19E+08 | 2.21E+08 | 2.01E+08 | 7.73E+08 | 7.77E+08 | 7.53E+08 | 2.14E+08 | 7.68E+08 | 1.00 : 3.59 |  |
|  | DLSRFYGHF | 52.14 | 1.52E+07 | 1.57E+07 | 1.43E+07 | 3.30E+06 | 3.76E+06 | 3.32E+06 | 1.51E+07 | 3.46E+06 | 1.00 : 0.23 |  |
| **Tachykinins (TK)** | ALMGFQGVRG | 33.23 | 1.98E+06 | 2.09E+06 | 1.92E+06 | 8.62E+05 | 8.72E+05 | 8.56E+05 | 2.00E+06 | 8.63E+05 | 1.00 : 0.43 |  |
|  | ALMGFQGVRa | 60 | 8.14E+08 | 8.19E+08 | 8.28E+08 | 3.37E+08 | 3.66E+08 | 3.58E+08 | 8.20E+08 | 3.54E+08 | 1.00 : 0.43 | Amidation |
|  | APMGFQGMRa | 60 | 9.27E+08 | 9.24E+08 | 9.17E+08 | 3.60E+08 | 3.69E+08 | 3.80E+08 | 9.23E+08 | 3.70E+08 | 1.00 : 0.4 | Amidation |
|  | APMGFQGMRG | 60 | 9.10E+06 | 9.29E+06 | 9.11E+06 | 3.36E+06 | 3.19E+06 | 3.23E+06 | 9.17E+06 | 3.26E+06 | 1.00 : 0.36 |  |
|  |  |  |  |  |  |  |  |  |  |  |  |  |
|  |  |  |  |  |  |  |  |  |  |  |  |  |
|  |  |  |  |  |  |  |  |  |  |  |  |  |
| **Protein** | **Peptide** | **Significance** | **ACC-PFs 1** | **ACC-PFs 2** | **ACC-PFs 3** | **AML-PFs 1** | **AML-PFs 2** | **AML-PFs 3** | **ACC-PFs** | **AML-PFs** | **Group Profile (Ratio)** | **PTM** |
| **Apidaecins** | GNNRPVYIPQPRPPHPRL | 60 | 1.38E+09 | 1.50E+09 | 1.46E+09 | 3.37E+09 | 3.60E+09 | 3.47E+09 | 1.447E+09 | 3.48E+09 | 1.00 : 2.41 |  |
| **Callisulfakinin** | pQQFDDYGHLRFa | 60 | 6.46E+06 | 6.42E+06 | 6.39E+06 | 4.16E+06 | 4.32E+06 | 4.14E+06 | 6423333.3 | 4206667 | 1.00 : 0.65 | Pyro-glu from Q; Amidation |
| **FMRFamide-related peptides-like** | GRNDLNFIRYa | 60 | 1.66E+07 | 1.45E+07 | 1.48E+07 | 4.77E+06 | 4.84E+06 | 4.44E+06 | 15300000 | 4683333 | 1.00 : 0.31 | Amidation |
| **Neuropeptide like precursor 1 (NPLP1)** | NVGSVAREHGLPYa | 60 | 2.21E+09 | 2.73E+09 | 2.22E+09 | 6.84E+09 | 6.84E+09 | 6.87E+09 | 2.39E+09 | 6.85E+09 | 1.00 : 2.87 | Amidation |
|  | YVASLARTGDLPIRa | 60 | 1.73E+07 | 1.77E+07 | 1.81E+07 | 3.39E+07 | 3.47E+07 | 3.55E+07 | 1.77E+07 | 47500000 | 1.00 : 1.96 | Amidation |
| **PBAN-type neuropeptide (PBAN)** | RVPWTPSPRLa | 60 | 7.45E+06 | 7.17E+06 | 7.21E+06 | 2.62E+07 | 2.50E+07 | 2.37E+07 | 7276666.7 | 24967000 | 1.00 : 3.43 | Amidation |
|  | GMWFGPRLa | 31.51 | 3.25E+06 | 3.45E+06 | 3.52E+06 | 7.93E+06 | 7.83E+06 | 8.00E+06 | 3406666.7 | 7920333 | 1.00 : 2.32 |  |
| **Prohormone-1** | LRNQLDIGDLQ | 60 | 4.46E+08 | 4.18E+08 | 4.51E+08 | 9.56E+08 | 9.48E+08 | 9.34E+08 | 438333333 | 9.46E+08 | 1.00 : 2.16 |  |
| **Prohormone-4** | IDLSRFYGHF | 43.86 | 1.95E+08 | 1.88E+08 | 1.72E+08 | 2.94E+08 | 3.07E+08 | 2.88E+08 | 185000000 | 2.96E+08 | 1.00 : 1.6 |  |
| **Tachykinins (TK)** | ALMGFQGVRa | 60 | 3.87E+09 | 3.66E+09 | 3.89E+09 | 1.47E+09 | 1.34E+09 | 1.32E+09 | 3.807E+09 | 1.38E+09 | 1.00 : 0.36 | Amidation |
|  | APMGFQGMRa | 60 | 4.58E+09 | 4.63E+09 | 4.55E+09 | 1.32E+09 | 1.24E+09 | 1.43E+09 | 4.587E+09 | 1.33E+09 | 1.00 : 0.29 | Amidation |
|  | ASFDDEYY | 60 | 3.49E+07 | 3.10E+07 | 3.38E+07 | 4.73E+06 | 4.93E+06 | 4.80E+06 | 3.32E+07 | 4.82E+06 | 1.00 : 0.14 |  |
|  |  |  |  |  |  |  |  |  |  |  |  |  |
|  |  |  |  |  |  |  |  |  |  |  |  |  |
|  |  |  |  |  |  |  |  |  |  |  |  |  |
| **Protein** | **Peptide** | **Significance** | **ACC-NFs 1** | **ACC-NFs 2** | **ACC-NFs 3** | **AML-NFs 1** | **AML-NFs 2** | **AML-NFs 3** | **ACC-NFs** | **AML-NFs** | **Group Profile (Ratio)** | **PTM** |
| **Diuretic hormone (DH)** | LVDHRIPDLENEMFDSGNDPGSTVVRT | 31.21 | 2.54E+06 | 2.31E+06 | 2.51E+06 | 7.87E+06 | 7.99E+06 | 8.03E+06 | 2.45E+06 | 7.96E+06 | 1.00 : 3.25 |  |
| **Neuropeptide like precursor 1 (NPLP1)** | SVSSLAKNSAWPVSL | 60 | 1.53E+08 | 1.55E+08 | 1.42E+08 | 2.62E+08 | 2.76E+08 | 2.89E+08 | 1.50E+08 | 2.76E+08 | 1.00 : 1.84 |  |
|  | NVASLARTYTLPQNAa | 27.49 | 4.77E+07 | 4.79E+07 | 4.99E+07 | 8.16E+07 | 8.31E+07 | 8.37E+07 | 4.85E+07 | 8.28E+07 | 1.00 : 1.71 | Amidation |
| **PBAN-type neuropeptide (PBAN)** | TSQDITSGMWFGPRLa | 60 | 2.38E+08 | 2.50E+08 | 2.47E+08 | 1.04E+09 | 1.02E+09 | 9.98E+08 | 2.45E+08 | 1.02E+09 | 1.00 : 4.16 | Amidation |
|  | pQITQFTPRLa | 46.66 | 4.20E+07 | 4.55E+07 | 4.41E+07 | 8.50E+07 | 8.36E+07 | 8.38E+07 | 4.39E+07 | 8.41E+07 | 1.00 : 1.92 | Pyro-glu from Q; Amidation |
| **Pigment-dispersing hormone (PDH)** | NSELINSLLGLPKNMNNAa | 60 | 3.02E+08 | 3.19E+08 | 3.18E+08 | 5.49E+07 | 5.67E+07 | 5.49E+07 | 3.13E+08 | 5.55E+07 | 1.00 : 0.18 | Amidation |
| **Prohormone-4** | IDLSRFYGHFNT | 39.91 | 6.23E+09 | 6.19E+09 | 6.34E+09 | 3.46E+09 | 3.50E+09 | 3.36E+09 | 6.25E+09 | 3.44E+09 | 1.00 : 0.55 |  |
| **Short neuropeptide F (sNPF)** | SPSLRLRFa | 28.41 | 5.84E+06 | 5.43E+06 | 5.37E+06 | 1.11E+07 | 1.12E+07 | 1.23E+07 | 5.55E+06 | 1.15E+07 | 1.00 : 2.08 | Amidation |
| **Tachykinins (TK)** | ALMGFQGVRa | 60 | 9.80E+09 | 9.58E+09 | 9.57E+09 | 3.57E+09 | 3.63E+09 | 3.39E+09 | 9.65E+09 | 3.53E+09 | 1.00 : 0.37 | Amidation |
|  | APMGFQGMRa | 55.7 | 9.93E+09 | 1.02E+10 | 9.93E+09 | 3.38E+09 | 3.27E+09 | 3.59E+09 | 1.00E+10 | 3.41E+09 | 1.00 : 0.34 | Amidation |
