# Supplementary material for: Tachykinin signaling inhibits task-specific behavioral responsiveness in honeybee workers
Source: eLife. 2021 Mar 24;10:e64830. doi: 10.7554/eLife.64830 (PMC8016481; doi:10.7554/eLife.64830)
Supplement: Figure 3—source data 1. [file elife-64830-fig3-data1.docx]

The proboscis extension response of workers after injection of ddH_2_O and TRP2. (manuscript section 2.3.1)

|  |  | **ddH_2_O** | | | **TRP2** | | |
| --- | --- | --- | --- | --- | --- | --- | --- |
| **Pollen foragers** | **Concentration** | **Show PER** | **No PER** | **PER ratio** | **Show PER** | **No PER** | **PER ratio** |
|  | **0.1%** | **19** | **36** | **34.55%** | **9** | **44** | **16.98%** |
|  | **0.3%** | **21** | **34** | **38.18%** | **11** | **42** | **20.75%** |
|  | **1.0%** | **30** | **25** | **54.55%** | **12** | **41** | **22.64%** |
|  | **3.0%** | **36** | **19** | **65.45%** | **15** | **38** | **28.30%** |
|  | **10.0%** | **38** | **17** | **69.09%** | **17** | **36** | **32.08%** |
|  | **30.0%** | **48** | **7** | **87.27%** | **25** | **28** | **47.17%** |
|  | **Pollen** | **22** | **34** | **39.29%** | **9** | **44** | **16.98%** |
|  | **Larva** | **11** | **45** | **19.64%** | **12** | **41** | **22.64%** |
|  |  |  |  |  |  |  |  |
|  |  | **ddH_2_O** | | | **TRP2** | | |
| **Nectar foragers** | **Concentration** | **Show PER** | **No PER** | **PER ratio** | **Show PER** | **No PER** | **PER ratio** |
|  | **0.1%** | **10** | **45** | **18.18%** | **6** | **52** | **10.34%** |
|  | **0.3%** | **14** | **41** | **25.45%** | **6** | **52** | **10.34%** |
|  | **1.0%** | **16** | **39** | **29.09%** | **7** | **51** | **12.07%** |
|  | **3.0%** | **19** | **36** | **34.55%** | **8** | **50** | **13.79%** |
|  | **10.0%** | **25** | **30** | **45.45%** | **12** | **46** | **20.69%** |
|  | **30.0%** | **29** | **26** | **52.73%** | **15** | **43** | **25.86%** |
|  | **Pollen** | **7** | **46** | **13.21%** | **8** | **44** | **15.38%** |
|  | **Larva** | **9** | **44** | **16.98%** | **6** | **46** | **11.54%** |
|  |  |  |  |  |  |  |  |
|  |  | **ddH_2_O** | | | **TRP2** | | |
| **Nurse bees** | **Concentration** | **Show PER** | **No PER** | **PER ratio** | **Show PER** | **No PER** | **PER ratio** |
|  | **0.1%** | **12** | **41** | **22.64%** | **8** | **44** | **15.38%** |
|  | **0.3%** | **13** | **40** | **24.53%** | **10** | **42** | **19.23%** |
|  | **1.0%** | **18** | **35** | **33.96%** | **13** | **39** | **25.00%** |
|  | **3.0%** | **19** | **34** | **35.85%** | **16** | **36** | **30.77%** |
|  | **10.0%** | **22** | **31** | **41.51%** | **21** | **31** | **40.38%** |
|  | **30.0%** | **29** | **24** | **54.72%** | **25** | **27** | **48.08%** |
|  | **Pollen** | **5** | **50** | **9.09%** | **7** | **48** | **12.73%** |
|  | **Larva** | **21** | **32** | **39.62%** | **10** | **45** | **18.18%** |
