# Supplementary material for: Tachykinin signaling inhibits task-specific behavioral responsiveness in honeybee workers
Source: eLife. 2021 Mar 24;10:e64830. doi: 10.7554/eLife.64830 (PMC8016481; doi:10.7554/eLife.64830)
Supplement: Figure 4—source data 1. [file elife-64830-fig4-data1.docx]

The proboscis extension response of workers after injection of ds*GFP*, ds*TRP*, and ds*TRPR*. (manuscript section 2.3.2)

|  |  | **ds*GFP*** | | | **ds*TRP*** | | | **ds*TRPR*** | | |
| --- | --- | --- | --- | --- | --- | --- | --- | --- | --- | --- |
| **Pollen foragers** | **Concentration** | **Show PER** | **No PER** | **PER ratio** | **Show PER** | **No PER** | **PER ratio** | **Show PER** | **No PER** | **PER ratio** |
|  | **0.1%** | **20** | **36** | **35.71%** | **30** | **24** | **55.56%** | **33** | **23** | **58.93%** |
|  | **0.3%** | **22** | **34** | **39.29%** | **33** | **21** | **61.11%** | **35** | **21** | **62.50%** |
|  | **1.0%** | **30** | **26** | **53.57%** | **40** | **14** | **74.07%** | **41** | **15** | **73.21%** |
|  | **3.0%** | **37** | **19** | **66.07%** | **41** | **13** | **75.93%** | **43** | **13** | **76.79%** |
|  | **10.0%** | **38** | **18** | **67.86%** | **45** | **9** | **83.33%** | **46** | **10** | **82.14%** |
|  | **30.0%** | **49** | **7** | **87.50%** | **51** | **3** | **94.44%** | **49** | **7** | **87.50%** |
|  | **Pollen** | **19** | **33** | **36.54%** | **32** | **20** | **61.54%** | **33** | **21** | **61.11%** |
|  | **Larva** | **10** | **42** | **19.23%** | **13** | **39** | **25.00%** | **12** | **42** | **22.22%** |
|  |  |  |  |  |  |  |  |  |  |  |
|  |  | **ds*GFP*** | | | **ds*TRP*** | | | **ds*TRPR*** | | |
| **Nectar foragers** | **Concentration** | **Show PER** | **No PER** | **PER ratio** | **Show PER** | **No PER** | **PER ratio** | **Show PER** | **No PER** | **PER ratio** |
|  | **0.1%** | **9** | **44** | **16.98%** | **17** | **33** | **34.00%** | **19** | **34** | **35.85%** |
|  | **0.3%** | **12** | **41** | **22.64%** | **22** | **28** | **44.00%** | **22** | **31** | **41.51%** |
|  | **1.0%** | **15** | **38** | **28.30%** | **27** | **23** | **54.00%** | **28** | **25** | **52.83%** |
|  | **3.0%** | **18** | **35** | **33.96%** | **29** | **21** | **58.00%** | **32** | **21** | **60.38%** |
|  | **10.0%** | **24** | **29** | **45.28%** | **31** | **19** | **62.00%** | **33** | **20** | **62.26%** |
|  | **30.0%** | **28** | **25** | **52.83%** | **34** | **16** | **68.00%** | **38** | **16** | **70.37%** |
|  | **Pollen** | **7** | **49** | **12.50%** | **13** | **42** | **23.64%** | **11** | **44** | **20.00%** |
|  | **Larva** | **10** | **46** | **17.86%** | **11** | **44** | **20.00%** | **12** | **43** | **21.82%** |
|  |  |  |  |  |  |  |  |  |  |  |
|  |  | **ds*GFP*** | | | **ds*TRP*** | | | **ds*TRPR*** | | |
| **Nurse bees** | **Concentration** | **Show PER** | **No PER** | **PER ratio** | **Show PER** | **No PER** | **PER ratio** | **Show PER** | **No PER** | **PER ratio** |
|  | **0.1%** | **12** | **43** | **21.82%** | **8** | **45** | **15.09%** | **9** | **46** | **16.36%** |
|  | **0.3%** | **13** | **42** | **23.64%** | **8** | **45** | **15.09%** | **13** | **42** | **23.64%** |
|  | **1.0%** | **18** | **37** | **32.73%** | **14** | **39** | **26.42%** | **16** | **39** | **29.09%** |
|  | **3.0%** | **20** | **35** | **36.36%** | **23** | **30** | **43.40%** | **25** | **30** | **45.45%** |
|  | **10.0%** | **23** | **32** | **41.82%** | **29** | **24** | **54.72%** | **27** | **28** | **49.09%** |
|  | **30.0%** | **30** | **25** | **54.55%** | **31** | **22** | **58.49%** | **33** | **22** | **60.00%** |
|  | **Pollen** | **6** | **50** | **10.71%** | **13** | **41** | **24.07%** | **12** | **43** | **21.82%** |
|  | **Larva** | **21** | **35** | **37.50%** | **31** | **23** | **57.41%** | **32** | **23** | **58.18%** |
