# Supplementary material for: Tachykinin signaling inhibits task-specific behavioral responsiveness in honeybee workers
Source: eLife. 2021 Mar 24;10:e64830. doi: 10.7554/eLife.64830 (PMC8016481; doi:10.7554/eLife.64830)
Supplement: Supplementary file 1. [file elife-64830-supp1.docx]

Statistical differences in sucrose responsiveness of different behavioral phenotypes. (manuscript section 2.1 Fig. 1A)

| **Concentration** | **0.10%** | **0.30%** | **1.00%** | **3.00%** | **10.00%** | **30.00%** |
| --- | --- | --- | --- | --- | --- | --- |
| **AML** |  |  |  |  |  |  |
| PFs vs NFs | *** | * | *** | *** | *** | *** |
| PFs vs NBs | ** | ** | *** | *** | *** | *** |
| NFs vs NBs | ns | ns | ns | ns | ns | ns |
| **ACC** |  |  |  |  |  |  |
| PFs vs NFs | ** | ** | *** | *** | ** | *** |
| PFs vs NBs | * | ** | ** | ** | ** | *** |
| NFs vs NBs | ns | ns | ns | ns | ns | ns |
| **AML vs ACC** |  |  |  |  |  |  |
| PFs | ns | * | * | ** | * | ns |
| NFs | ns | * | * | * | ns | ns |
| NBs | ns | ns | ns | ns | ns | ns |
| **AML**: *Apis mellifera ligustica*, **ACC**: *Apis cerana cerana*, **PFs**: pollen foragers, **NFs**: nectar foragers, **NBs**: nurse bees. ns = *p* > 0.05, *: *p* < 0.05, **: *p* < 0.01, ***: *p* < 0.001 | | | | | | |
