# Supplementary material for: Tachykinin signaling inhibits task-specific behavioral responsiveness in honeybee workers
Source: eLife. 2021 Mar 24;10:e64830. doi: 10.7554/eLife.64830 (PMC8016481; doi:10.7554/eLife.64830)
Supplement: Supplementary file 2. [file elife-64830-supp2.docx]

Neuropeptides identified in the brain of *Apis mellifera ligustica* workers. (manuscript section 2.2)

"**NBs**": nurse bees. "**PFs**": pollen foragers. "**NFs**": nectar foragers. "**Protein Accession**": the unique number given to mark the entry of a protein in the database NCBInr. "**Peptide**": the amino acid sequence of the peptide as determined in PEAKS Search. "**-10lgP**": the score indicates the scoring significance of a peptide-spectrum match. "**Mass**": the monoisotopic mass of the peptide. "**ppm**": the precursor mass error, calculated as 10^6^ × (precursor mass - peptide mass) / peptide mass. "**m/z**": the precursor mass-to-charge ratio. "**z**": the peptide charge. "**RT**": the retention time (elution time) of the spectrum as recorded in the data. "**#Spec**": the number of scanned spectrums of the peptide. "**PTM**": the post translational modification types present in the peptide.

| **Sample** | **Protein Accession** | **Peptide** | **10lgP** | **Mass** | **ppm** | **m/z** | **z** | **RT** | **#Spec** | **PTM** |
| --- | --- | --- | --- | --- | --- | --- | --- | --- | --- | --- |
| NBs | Q868G6.1 | NSIINDVKNELFPEDIN | 67.29 | 1972.974 | -0.3 | 987.494 | 2 | 98.51 | 10 |  |
| NBs | Q868G6.1 | VLSMDGYQNILDKKDELLGEWE | 61.58 | 2594.257 | -7 | 1298.127 | 2 | 96.42 | 10 |  |
| NBs | A8CL69.1 | pQLHNIVDKPRQN | 51.73 | 1443.758 | 0.7 | 482.2603 | 3 | 13.7 | 6 | Pyro-glu from Q |
| NBs | A8CL69.1 | pQLHNIVDKPRQNFNDPRF | 51.12 | 2220.119 | 0.3 | 556.0372 | 4 | 41.34 | 6 | Pyro-glu from Q |
| NBs | A8CL69.1 | TSQDITSGMWFGPRLa | 47.39 | 1693.825 | 0.1 | 847.9196 | 2 | 80.85 | 11 | Amidation |
| NBs | A8CL69.1 | pQLHNIVDKP | 45.99 | 1045.556 | 0.8 | 523.7855 | 2 | 23.83 | 4 | Pyro-glu from Q |
| NBs | A8CL69.1 | GMWFGPRLa | 33.41 | 961.4956 | 0 | 481.7551 | 2 | 68.13 | 9 | Amidation |
| NBs | A8CL69.1 | RVPWTPSPRLa | 30.85 | 1206.699 | 0.3 | 604.3567 | 2 | 25.21 | 6 | Amidation |
| NBs | A8CL69.1 | pQITQFTPRL | 27.13 | 1085.587 | -0.1 | 543.8007 | 2 | 78.09 | 3 | Pyro-glu from Q |
| NBs | A8CL69.1 | MWFGPRLa | 26.77 | 904.4741 | -0.5 | 453.2441 | 2 | 71.2 | 5 | Amidation |
| NBs | A8CL69.1 | QITQFTPRLa | 25.1 | 1101.63 | 0.5 | 551.8223 | 2 | 34.24 | 12 | Amidation |
| NBs | A8CL69.1 | pQITQFTPRLa | 37.99 | 1084.603 | 0 | 543.3087 | 2 | 69.95 | 21 | Pyro-glu from Q; Amidation |
| NBs | ACI90290.1 | TWKSPDIVIRFa | 50.93 | 1359.766 | -0.3 | 454.2625 | 3 | 59.62 | 13 | Amidation |
| NBs | ACI90290.1 | GRNDLNFIRYa | 48.35 | 1265.663 | -0.1 | 633.8386 | 2 | 33.11 | 11 | Amidation |
| NBs | NP_001161192.1 | PEIFTSPEELRRYIDHVSDYYLLSGKARYa | 43.49 | 3515.784 | 0.4 | 586.9714 | 6 | 95.9 | 5 | Amidation |
| NBs | P85527.1 | QDVDHVFLRFa | 55.21 | 1273.657 | 0 | 637.8356 | 2 | 50.66 | 9 | Amidation |
| NBs | P85527.1 | pQDVDHVFLRFa | 53.95 | 1256.63 | -0.7 | 629.3219 | 2 | 74.39 | 15 | Pyro-glu from Q; Amidation |
| NBs | P85527.1 | pQDVDHVFLRF | 47.89 | 1257.614 | 0.8 | 629.8148 | 2 | 79.69 | 5 | Pyro-glu from Q |
| NBs | P85527.1 | pQDVDHVFLR | 47.86 | 1110.546 | -0.5 | 556.2799 | 2 | 43.23 | 7 | Pyro-glu from Q |
| NBs | P85527.1 | pQDVDHVFL | 28.42 | 954.4447 | 1.8 | 478.2305 | 2 | 69.45 | 5 | Pyro-glu from Q |
| NBs | P85798.1 | LRNQLDIGDLQ | 50.23 | 1283.683 | -0.5 | 642.8486 | 2 | 42.48 | 10 |  |
| NBs | P85798.1 | IPAADKERLLN | 47.66 | 1238.698 | 0.9 | 620.3569 | 2 | 15.09 | 6 |  |
| NBs | P85798.1 | LRNQLDIGDL | 38.2 | 1155.625 | 0 | 578.8196 | 2 | 51.53 | 5 |  |
| NBs | P85799.1 | SQAYDPYSNAAQFQLSSQSRGYPYQHRLVY | 71.75 | 3523.655 | -0.6 | 881.9204 | 4 | 60.92 | 51 |  |
| NBs | P85799.1 | SQAYDPYSNAAQFQLSSQSRGYPYQHRLV | 70.34 | 3360.591 | -0.4 | 841.1547 | 4 | 57.58 | 11 |  |
| NBs | P85799.1 | SQAYDPYSNAAQFQLSSQSRGYPYQHRL | 64.21 | 3261.523 | -1.2 | 816.387 | 4 | 53.22 | 8 |  |
| NBs | P85799.1 | LPTNLAEDTKKTEQTMRPKS | 55.28 | 2287.184 | 0.1 | 572.8033 | 4 | 21.04 | 22 |  |
| NBs | P85799.1 | GYPYQHRLVY | 49.18 | 1294.646 | 0.4 | 648.3304 | 2 | 25.22 | 15 |  |
| NBs | P85799.1 | NVPIYQEPRF | 46 | 1261.646 | -0.3 | 631.8298 | 2 | 46.32 | 11 |  |
| NBs | P85799.1 | VPIYQEPRF | 43.34 | 1147.603 | -0.1 | 574.8085 | 2 | 42.88 | 6 |  |
| NBs | P85799.1 | PIYQEPRF | 28.55 | 1048.534 | 0.4 | 525.2746 | 2 | 46.03 | 7 |  |
| NBs | P85828.1 | ITGQGNRIF | 46.68 | 1004.54 | -0.7 | 503.2771 | 2 | 19.63 | 8 |  |
| NBs | P85828.1 | SLKAPFA | 41.78 | 732.417 | -0.6 | 367.2155 | 2 | 22.72 | 5 |  |
| NBs | P85828.1 | SLKAPF | 36.02 | 661.3799 | 0.3 | 331.6973 | 2 | 23.77 | 4 |  |
| NBs | P85829.1 | MVPVPVHHMADELLRNGPDTVI | 62.4 | 2439.24 | 0 | 1220.627 | 2 | 76.66 | 17 |  |
| NBs | P85829.1 | VHHMADELLRNGPDTVI | 49.65 | 1915.957 | 0.1 | 639.6598 | 3 | 46.23 | 8 |  |
| NBs | P85829.1 | VPVPVHHMADELL | 43.49 | 1455.754 | 1.3 | 728.8854 | 2 | 47.73 | 7 |  |
| NBs | P85829.1 | LLRNGPDTVI | 35 | 1096.624 | 0.2 | 549.3194 | 2 | 28.64 | 5 |  |
| NBs | P85829.1 | LRNGPDTVI | 22.27 | 983.54 | 0.5 | 492.7775 | 2 | 16.98 | 6 |  |
| NBs | P85830.1 | GLDLGLSRGFSGSQAAKHLMGLAAANYAGGPa | 70.62 | 2985.524 | -0.9 | 996.1811 | 3 | 84.68 | 9 | Amidation |
| NBs | P85830.1 | GLDLGLSRGFSGSQAA | 62.96 | 1534.774 | 1.2 | 768.3951 | 2 | 55.2 | 6 |  |
| NBs | P85830.1 | GLDLGLSRGFSGSQAAKH | 53.33 | 1799.928 | -0.5 | 600.9829 | 3 | 31.47 | 10 |  |
| NBs | P85830.1 | GLDLGLSRGFSGSQAAKHLMa | 46.31 | 2043.068 | 1 | 682.0308 | 3 | 57.54 | 8 | Amidation |
| NBs | P85831.1 | IDLSRFYGHFNT | 60.52 | 1468.71 | -0.3 | 735.362 | 2 | 64.92 | 29 |  |
| NBs | P85831.1 | IDLSRFYGHFN | 56.71 | 1367.662 | -0.1 | 684.8383 | 2 | 62.93 | 18 |  |
| NBs | P85831.1 | IDLSRFYGHF | 52.75 | 1253.619 | -0.6 | 627.8165 | 2 | 70.59 | 19 |  |
| NBs | P85831.1 | IDLSRFYGHFNTKR | 48.89 | 1752.906 | 0 | 439.2337 | 4 | 43.28 | 23 |  |
| NBs | P85831.1 | FYGHFNT | 44.93 | 884.3817 | -0.2 | 443.1981 | 2 | 21.44 | 7 |  |
| NBs | P85831.1 | DLSRFYGHF | 25.85 | 1140.535 | 0.2 | 571.275 | 2 | 70.16 | 3 |  |
| NBs | P85831.1 | DLSRFYGHFN | 20.23 | 1254.578 | 0.4 | 628.2966 | 2 | 62.68 | 22 |  |
| NBs | P85832.1 | LTNYLATTGHGTNTGGPVLT | 82.04 | 1987.001 | -1.4 | 994.5065 | 2 | 47.57 | 22 |  |
| NBs | P85832.1 | LTNYLATTGHGTNTGGPVL | 69.52 | 1885.953 | -0.6 | 943.9834 | 2 | 52.3 | 4 |  |
| NBs | P85832.1 | NLDEIDRVGWSGFV | 62.73 | 1605.779 | 0.3 | 803.8969 | 2 | 88.41 | 3 |  |
| NBs | P85832.1 | LTNYLATTGHGTNTGGPVLTRRFa | 49.49 | 2445.288 | -0.4 | 816.1028 | 3 | 39.57 | 13 | Amidation |
| NBs | P85832.1 | NIDEIDRTAFDNFF | 46.68 | 1715.779 | -1.2 | 858.8958 | 2 | 96.46 | 9 |  |
| NBs | P85832.1 | LVDELSPVSERETLERFa | 33.35 | 2017.048 | 0.3 | 673.3568 | 3 | 63.4 | 7 | Amidation |
| NBs | P85832.1 | ELVDELSPVSERETLERFa | 30.33 | 2146.091 | 0.6 | 716.3712 | 3 | 74.41 | 9 | Amidation |
| NBs | Q06601.1 | GNNRPVYIPQPRPPHPRL | 33.24 | 2107.155 | 0.3 | 422.4384 | 5 | 25.01 | 15 |  |
| NBs | Q06601.1 | VYIPQPRPPHPRL | 23.32 | 1568.894 | -0.2 | 393.2307 | 4 | 29.92 | 20 |  |
| NBs | Q06601.1 | AVHYSGGQPLGSKRPNDMLSQRYHFGLa | 65.31 | 3013.509 | -1.6 | 754.3834 | 4 | 38.67 | 9 | Amidation |
| NBs | Q06601.1 | PNDMLSQRYHFGLa | 66.71 | 1575.762 | 0.3 | 526.2613 | 3 | 56.93 | 13 | Amidation |
| NBs | Q06601.1 | AYTYVSEYKRLPVYNFGIa | 29.98 | 2181.126 | -0.2 | 728.0491 | 3 | 72.4 | 8 | Amidation |
| NBs | Q06601.1 | ADYPLRLNLD | 48.67 | 1188.614 | 0 | 595.3142 | 2 | 56.85 | 11 |  |
| NBs | Q06601.1 | YPLRLNLD | 43.48 | 1002.55 | 0.6 | 502.2825 | 2 | 49.18 | 8 |  |
| NBs | Q06601.1 | RQYSFGLa | 31.09 | 868.4555 | 0 | 435.235 | 2 | 26.91 | 10 | Amidation |
| NBs | Q06601.1 | GRQPYSFGLa | 35.27 | 1022.53 | -0.1 | 512.2721 | 2 | 32.39 | 6 | Amidation |
| NBs | Q06601.1 | GRDYSFGLa | 31.03 | 912.4453 | 0 | 457.2299 | 2 | 30.51 | 3 | Amidation |
| NBs | Q06601.1 | WIDTNDNKRGRDYSFGLa | 29.02 | 2054.992 | 0 | 686.0047 | 3 | 38.47 | 7 | Amidation |
| NBs | Q06601.1 | LDYLPVDNPAFH | 51.58 | 1399.677 | 0.6 | 700.8463 | 8 | 65.51 | 4 |  |
| NBs | Q06602.1 | EAEPEAEPGNNRPVYIPQPRPPHPRL | 50.05 | 2959.505 | -0.5 | 592.908 | 5 | 33.75 | 26 |  |
| NBs | Q06602.1 | GNNRPVYIPQPRPPHPRL | 33.24 | 2107.155 | 0.3 | 422.4384 | 5 | 25.01 | 15 |  |
| NBs | Q06602.1 | VYIPQPRPPHPRL | 23.32 | 1568.894 | -0.2 | 393.2307 | 4 | 29.92 | 20 |  |
| NBs | Q5DW47.1 | STSLEELANR | 39.7 | 1118.557 | 0.9 | 560.2861 | 2 | 24.18 | 4 |  |
| NBs | Q5DW47.1 | STSLEELANRN | 38.16 | 1232.6 | 0.7 | 617.3075 | 2 | 23.07 | 5 |  |
| NBs | Q5DW47.1 | pQTFTYSHGWTNa | 18.99 | 1322.568 | -0.1 | 662.2912 | 2 | 51.14 | 10 | Pyro-glu from Q; Amidation |
| NBs | Q868G6.1 | ASFDDEYYKRAPMGFQGMRa | 55.4 | 2267.025 | 0.5 | 567.7639 | 4 | 45.63 | 9 | Amidation |
| NBs | Q868G6.1 | APMGFQGMRG | 50.96 | 1050.474 | 0 | 526.2442 | 2 | 22.9 | 6 |  |
| NBs | Q868G6.1 | GVMDFQIGLQ | 50.62 | 1106.543 | 1 | 554.2793 | 2 | 85.81 | 6 |  |
| NBs | Q868G6.1 | APMGFQGMRa | 49.03 | 992.4684 | -1.1 | 497.241 | 2 | 18.87 | 16 | Amidation |
| NBs | Q868G6.1 | VLSMDGYQNILD | 47.52 | 1366.644 | 0.6 | 684.3296 | 2 | 80.98 | 15 |  |
| NBs | Q868G6.1 | NPRWEFRGKFVGVRa | 47.04 | 1745.959 | -0.2 | 437.4969 | 4 | 24.64 | 9 | Amidation |
| NBs | Q868G6.1 | ARMGFHGMRa | 46.29 | 1060.517 | -0.4 | 354.5128 | 3 | 8.86 | 3 | Amidation |
| NBs | Q868G6.1 | ALMGFQGVRG | 46.07 | 1034.533 | 0.1 | 518.2739 | 2 | 35.2 | 6 |  |
| NBs | Q868G6.1 | SPFRYLGA | 45.4 | 909.4708 | 0 | 455.7427 | 2 | 36.86 | 10 |  |
| NBs | Q868G6.1 | APMGFYGTRa | 45.2 | 997.4803 | -0.1 | 499.7474 | 2 | 16.94 | 3 | Amidation |
| NBs | Q868G6.1 | APMGFYGTRG | 45.18 | 1055.486 | 0.4 | 528.7504 | 2 | 20.63 | 7 |  |
| NBs | Q868G6.1 | ALMGFQGVRa | 44.3 | 976.5276 | -0.5 | 489.2709 | 2 | 29.63 | 13 | Amidation |
| NBs | Q868G6.1 | SPFRYLGARG | 44.18 | 1122.593 | -0.4 | 375.2049 | 3 | 20.59 | 11 |  |
| NBs | Q868G6.1 | GVMDFQIGLQRKKD | 44.03 | 1633.861 | -0.2 | 817.9376 | 2 | 35.7 | 14 |  |
| NBs | Q868G6.1 | SPFRYLGARa | 43.32 | 1064.588 | 0.2 | 355.87 | 3 | 16.59 | 8 | Amidation |
| NBs | Q868G6.1 | NPRWEFRGKFVGV | 42.84 | 1590.842 | 0.1 | 531.288 | 3 | 43.57 | 15 |  |
| NBs | Q868G6.1 | SPFRYLG | 37.59 | 838.4337 | 0 | 420.2241 | 2 | 31.97 | 7 |  |
| NBs | Q868G6.1 | SLEEILDEIK | 33.02 | 1187.629 | 0 | 594.8215 | 2 | 88.28 | 6 |  |
| NBs | Q868G6.1 | SLEEILDEI | 29.37 | 1059.534 | 0.1 | 530.7741 | 2 | 108.02 | 4 |  |
| NBs | Q868G6.1 | ASFDDEYY | 28.99 | 1008.371 | 0 | 505.1929 | 2 | 43.35 | 4 |  |
| NBs | XP_006557714.1 | pQQFDDYGHLRFa | 47.97 | 1406.637 | -2.3 | 704.324 | 2 | 68.3 | 4 | Pyro-glu from Q; Amidation |
| NBs | XP_006559359.1 | NVASLARTYTLPQNAa | 64.35 | 1616.863 | -1.3 | 809.4379 | 2 | 43.18 | 6 | Amidation |
| NBs | XP_006559359.1 | SVSSLAKNSAWPVSL | 62.69 | 1544.82 | -1.3 | 773.4162 | 2 | 68.52 | 8 |  |
| NBs | XP_006559359.1 | FLLLPATDNNYFHQKLPSSLRSKSL | 56.55 | 2888.555 | 1 | 578.7188 | 5 | 71.13 | 15 |  |
| NBs | XP_006559359.1 | NVGSVAREHGLPYa | 55.04 | 1396.721 | -0.8 | 699.3672 | 2 | 21.03 | 15 | Amidation |
| NBs | XP_006559359.1 | SVSSLARTGDLPVREQ | 53.68 | 1713.901 | 0.5 | 572.3079 | 3 | 25.97 | 12 |  |
| NBs | XP_006559359.1 | YVASLARTGDLPIRGQ | 51.94 | 1715.932 | 0.4 | 572.9847 | 3 | 35.62 | 12 |  |
| NBs | XP_006559359.1 | NIASLMRDYDQSRENRVPFPa | 47.38 | 2406.186 | 0.1 | 803.0695 | 3 | 63.84 | 12 | Amidation |
| NBs | XP_006559359.1 | HIGALARLGWLPSLRTA | 42.32 | 1831.058 | -0.2 | 611.3598 | 3 | 70.88 | 7 |  |
| NBs | XP_006559359.1 | HIGALARLGWLPSLRTARFS | 42 | 2221.26 | -0.4 | 556.322 | 4 | 71.36 | 9 |  |
| NBs | XP_006559359.1 | NVGTLARDFALPPa | 40.53 | 1368.751 | -0.1 | 685.3829 | 2 | 60.79 | 16 | Amidation |
| NBs | XP_006559359.1 | YVASLARTGDLPIRa | 40.29 | 1529.868 | 0.6 | 510.9635 | 3 | 34.02 | 8 | Amidation |
| NBs | XP_006559359.1 | GIFLPGSVILR | 37.83 | 1170.712 | -0.1 | 586.3634 | 2 | 77.93 | 5 |  |
| NBs | XP_006559359.1 | LPGSVILRALS | 36.35 | 1124.692 | 2.1 | 563.3543 | 2 | 72.8 | 8 |  |
| NBs | XP_006559359.1 | GIFLPGSVILRALSRQa | 36.3 | 1725.041 | -0.9 | 576.0205 | 3 | 95.14 | 10 | Amidation |
| NBs | XP_006559359.1 | NVGTLARDFALPPGRRNIASLMRDYDQSRENRVPFPa | 21.57 | 4127.135 | 0.2 | 688.8632 | 6 | 75.08 | 9 | Amidation |
| NBs | XP_006559865.1 | AFGLLTYPRIa | 40.74 | 1148.671 | 0.5 | 575.3428 | 2 | 70.98 | 6 | Amidation |
| NBs | XP_006559865.1 | SNAPISNLNFN | 35.48 | 1189.573 | 0.3 | 595.7938 | 2 | 48.7 | 4 |  |
| NBs | XP_006559865.1 | EKLKPNMRRAFGLLTYPRIa | 28.33 | 2301.325 | 0.6 | 576.339 | 4 | 50.2 | 8 | Amidation |
| NBs | XP_006560385.1 | AYRKPPFNGSIFa | 42.26 | 1394.746 | -0.5 | 698.3799 | 2 | 36.92 | 12 | Amidation |
| NBs | XP_006560385.1 | KPPFNGSIFa | 39.41 | 1004.544 | 0.2 | 503.2795 | 2 | 43.74 | 6 | Amidation |
| NBs | XP_006560385.1 | RKPPFNGSIFa | 32.35 | 1160.645 | 0 | 581.33 | 2 | 28.38 | 7 | Amidation |
| NBs | XP_006560385.1 | YRKPPFNGSIFa | 25.22 | 1323.709 | 0.5 | 662.862 | 2 | 36.42 | 6 | Amidation |
| NBs | XP_006562922.1 | GFKPEYISTAYGFa | 40.22 | 1477.724 | 0.2 | 739.8695 | 2 | 64.18 | 4 | Amidation |
| NBs | XP_006565207.1 | SDPHLSILSKPMSAIPSYKFDD | 81.44 | 2447.204 | 0.4 | 816.7423 | 3 | 71.96 | 17 |  |
| NBs | XP_006565207.1 | SPSLRLRFa | 40.12 | 973.5821 | 0.2 | 487.7984 | 2 | 24.53 | 13 | Amidation |
| NBs | XP_006565207.1 | SDPHLSILS | 39.05 | 967.4974 | 0.4 | 484.7562 | 2 | 34.74 | 7 |  |
| NBs | XP_006565207.1 | SQRSPSLRLRFa | 38.06 | 1344.774 | 0.4 | 449.2654 | 3 | 16.83 | 10 | Amidation |
| NBs | XP_006565207.1 | SDPHLSILSKPMSAIP | 32.57 | 1691.892 | -1.1 | 846.9521 | 2 | 64.26 | 4 |  |
| NBs | XP_006570344.1 | NSELINSLLGLPKNMNNAa | 65.94 | 1940.015 | 0.5 | 971.0152 | 2 | 87.45 | 11 | Amidation |
| NBs | XP_006570344.1 | LINSLLGLPKNMNNAa | 35.9 | 1609.897 | 1.1 | 805.9568 | 2 | 62.46 | 6 | Amidation |
| NBs | XP_016769998.1 | LVDHRIPDLENEMFDSGNDPGSTVVRT | 78.07 | 3012.425 | 0.1 | 754.1135 | 4 | 63.86 | 16 |  |
| NBs | XP_016769998.1 | HPISYNTYDERELSRDHPPLLL | 30.5 | 2664.33 | -1.6 | 667.0886 | 4 | 54.55 | 3 |  |
| NBs | XP_016769998.1 | IGSLSIVNSMDVLRQRVLLELARRKALQDQAQIDANRRLLETIa | 27.71 | 4913.782 | -0.3 | 819.9707 | 6 | 98.39 | 12 | Amidation |
| PFs | Q868G6.1 | NSIINDVKNELFPEDIN | 48.21 | 1972.974 | -0.3 | 987.494 | 2 | 100.35 | 14 |  |
| PFs | A8CL69.1 | TSQDITSGMWFGPRLa | 42.05 | 1693.825 | 0.5 | 847.92 | 2 | 79 | 22 | Amidation |
| PFs | A8CL69.1 | pQLHNIVDKPRQN | 37.86 | 1443.758 | 0.3 | 482.2602 | 3 | 14.35 | 3 | Pyro-glu from Q |
| PFs | A8CL69.1 | pQLHNIVDKP | 36.78 | 1045.556 | 0 | 523.7851 | 2 | 25.52 | 6 | Pyro-glu from Q |
| PFs | A8CL69.1 | pQITQFTPRLa | 29.72 | 1084.603 | 0.4 | 543.309 | 2 | 68.45 | 3 | Pyro-glu from Q; Amidation |
| PFs | A8CL69.1 | RVPWTPSPRLa | 25.44 | 1206.699 | 1.6 | 604.3575 | 2 | 28.86 | 5 | Amidation |
| PFs | A8CL69.1 | pQLHNIVDKPRQNFNDPRF | 23.63 | 2220.119 | -0.9 | 556.0365 | 4 | 47.19 | 4 | Pyro-glu from Q |
| PFs | A8CL69.1 | QITQFTPRLa | 22.79 | 1101.63 | -0.5 | 551.8218 | 2 | 37.4 | 7 | Amidation |
| PFs | A8CL69.1 | MWFGPRLa | 20.27 | 904.4741 | -0.2 | 453.2443 | 2 | 75.27 | 10 | Amidation |
| PFs | A8CL69.1 | GMWFGPRLa | 17.98 | 961.4956 | 0.7 | 481.7554 | 2 | 72.51 | 12 | Amidation |
| PFs | ACI90290.1 | TWKSPDIVIRFa | 42 | 1359.766 | 0.1 | 454.2627 | 3 | 63.04 | 13 | Amidation |
| PFs | ACI90290.1 | GRNDLNFIRYa | 36.79 | 1265.663 | 0.2 | 633.8388 | 2 | 37.18 | 24 | Amidation |
| PFs | ACI90290.1 | AGFKNLNREQ | 35.46 | 1175.605 | 0 | 392.8755 | 3 | 10.1 | 6 |  |
| PFs | ACI90290.1 | SPDIVIRFa | 28.69 | 944.5443 | -1.1 | 473.2789 | 2 | 51.32 | 7 | Amidation |
| PFs | NP_001161192.1 | PEIFTSPEELRRYIDHVSDYYLLSGKARYa | 45.15 | 3515.784 | 0.4 | 586.9714 | 6 | 95.9 | 8 | Amidation |
| PFs | P85527.1 | pQDVDHVFLRFa | 43.1 | 1256.63 | -0.6 | 629.322 | 2 | 76.1 | 19 | Pyro-glu from Q; Amidation |
| PFs | P85527.1 | pQDVDHVFLR | 40.39 | 1110.546 | 0.1 | 556.2802 | 2 | 44.76 | 7 | Pyro-glu from Q |
| PFs | P85527.1 | QDVDHVFLRFa | 40.1 | 1273.657 | 0.1 | 637.8357 | 2 | 54.09 | 9 | Amidation |
| PFs | P85527.1 | pQDVDHVFLRF | 38.93 | 1257.614 | 0 | 629.8143 | 2 | 82.73 | 8 | Pyro-glu from Q |
| PFs | P85527.1 | pQDVDHVFL | 27.57 | 954.4447 | 0.2 | 478.2297 | 2 | 70.88 | 3 | Pyro-glu from Q |
| PFs | P85798.1 | LRNQLDIGDLQ | 38.97 | 1283.683 | -1 | 642.8483 | 2 | 44.49 | 12 |  |
| PFs | P85798.1 | IPAADKERLLN | 33.42 | 1238.698 | 1.2 | 413.9072 | 3 | 15.96 | 6 |  |
| PFs | P85798.1 | LRNQLDIGDL | 31.19 | 1155.625 | 0.3 | 578.8198 | 2 | 54.4 | 11 |  |
| PFs | P85799.1 | SQAYDPYSNAAQFQLSSQSRGYPYQHRLVY | 51.05 | 3523.655 | -0.1 | 881.9208 | 4 | 64.95 | 41 |  |
| PFs | P85799.1 | SQAYDPYSNAAQFQLSSQSRGYPYQHRLV | 42.31 | 3360.591 | 0.4 | 841.1554 | 4 | 59.88 | 14 |  |
| PFs | P85799.1 | LPTNLAEDTKKTEQTMRPKS | 37.87 | 2287.184 | 0.3 | 572.8035 | 4 | 24.72 | 28 |  |
| PFs | P85799.1 | SQAYDPYSNAAQFQLSSQSRGYPYQHRL | 37.71 | 3261.523 | -3.4 | 816.3852 | 4 | 56.14 | 10 |  |
| PFs | P85799.1 | GYPYQHRLVY | 37.44 | 1294.646 | 0.3 | 648.3304 | 2 | 29.02 | 28 |  |
| PFs | P85799.1 | NVPIYQEPRF | 37.08 | 1261.646 | -1.1 | 631.8293 | 2 | 48.46 | 16 |  |
| PFs | P85799.1 | VPIYQEPRF | 35.84 | 1147.603 | -0.1 | 574.8085 | 2 | 45.61 | 13 |  |
| PFs | P85799.1 | PIYQEPRF | 25.33 | 1048.534 | 0.1 | 525.2744 | 2 | 49.81 | 6 |  |
| PFs | P85828.1 | ITGQGNRIF | 37.41 | 1004.54 | -0.8 | 503.277 | 2 | 21.59 | 21 |  |
| PFs | P85828.1 | SLKAPFA | 34.04 | 732.417 | 0.1 | 367.2158 | 2 | 24.36 | 11 |  |
| PFs | P85828.1 | SLKAPF | 29.98 | 661.3799 | 0.1 | 331.6973 | 2 | 26.29 | 15 |  |
| PFs | P85829.1 | MVPVPVHHMADELLRNGPDTVI | 36.48 | 2439.24 | 0.7 | 814.0879 | 3 | 81.53 | 22 |  |
| PFs | P85829.1 | VHHMADELLRNGPDTVI | 33.81 | 1915.957 | 1.1 | 639.6605 | 3 | 51.24 | 10 |  |
| PFs | P85829.1 | LLRNGPDTVI | 31.63 | 1096.624 | 0.4 | 549.3195 | 2 | 30.43 | 6 |  |
| PFs | P85829.1 | LRNGPDTVI | 28.06 | 983.54 | 0.1 | 492.7773 | 2 | 17.56 | 6 |  |
| PFs | P85829.1 | VPVPVHHMADELL | 20.81 | 1455.754 | 0.4 | 486.2589 | 3 | 51.21 | 6 |  |
| PFs | P85830.1 | GLDLGLSRGFSGSQAAKHLMGLAAANYAGGPa | 46.77 | 2985.524 | -0.3 | 747.3881 | 4 | 90.26 | 20 | Amidation |
| PFs | P85830.1 | GLDLGLSRGFSGSQAA | 43.99 | 1534.774 | 0.5 | 768.3947 | 2 | 57.69 | 5 |  |
| PFs | P85830.1 | HLMGLAAANYAGGPa | 32.71 | 1340.666 | -0.7 | 671.3398 | 2 | 41.33 | 7 | Amidation |
| PFs | P85830.1 | GLDLGLSRGFSGSQAAKHLMa | 31.39 | 2043.068 | 0.5 | 682.0304 | 3 | 61.51 | 6 | Amidation |
| PFs | P85830.1 | GLDLGLSRGFSGSQAAKH | 28.75 | 1799.928 | 0.1 | 600.9833 | 3 | 34.79 | 8 |  |
| PFs | P85831.1 | IDLSRFYGHFNT | 45.95 | 1468.71 | -0.5 | 735.3618 | 2 | 69.86 | 42 |  |
| PFs | P85831.1 | IDLSRFYGHFN | 42.44 | 1367.662 | -1.9 | 684.8371 | 2 | 65.48 | 27 |  |
| PFs | P85831.1 | IDLSRFYGHF | 41.39 | 1253.619 | -0.6 | 627.8165 | 2 | 74.6 | 23 |  |
| PFs | P85831.1 | FYGHFNT | 36.1 | 884.3817 | 0.6 | 443.1984 | 2 | 23.94 | 9 |  |
| PFs | P85831.1 | DLSRFYGHFN | 34.65 | 1254.578 | 0.1 | 628.2964 | 2 | 65.54 | 4 |  |
| PFs | P85831.1 | IDLSRFYGHFNTKR | 32.23 | 1752.906 | -0.1 | 439.2337 | 4 | 49.93 | 10 |  |
| PFs | P85832.1 | LTNYLATTGHGTNTGGPVLT | 52.74 | 1987.001 | 0.2 | 994.5081 | 2 | 50.02 | 12 |  |
| PFs | P85832.1 | NLDEIDRVGWSGFV | 47.92 | 1605.779 | 0 | 803.8966 | 2 | 93.23 | 14 |  |
| PFs | P85832.1 | LTNYLATTGHGTNTGGPVL | 45.56 | 1885.953 | 0.3 | 943.9843 | 2 | 54.77 | 5 |  |
| PFs | P85832.1 | NIDEIDRTAFDNFF | 43.44 | 1715.779 | 1.3 | 858.8979 | 2 | 98.86 | 7 |  |
| PFs | P85832.1 | LTNYLATTGHGTNTGGPVLTRRFa | 37.72 | 2445.288 | 0.5 | 612.3295 | 4 | 44.18 | 11 | Amidation |
| PFs | P85832.1 | ELVDELSPVSERETLERFa | 32.49 | 2146.091 | 0.9 | 716.3715 | 3 | 77.64 | 9 | Amidation |
| PFs | P85832.1 | LVDELSPVSERETLERFa | 31.14 | 2017.048 | -0.5 | 673.3563 | 3 | 66.54 | 10 | Amidation |
| PFs | Q06601.1 | AVHYSGGQPLGSKRPNDMLSQRYHFGLa | 60.6 | 3013.509 | 0.6 | 754.3851 | 4 | 40.53 | 8 | Amidation |
| PFs | Q06601.1 | PNDMLSQRYHFGLa | 65.73 | 1575.762 | 0.1 | 788.8882 | 2 | 47.77 | 9 | Amidation |
| PFs | Q06601.1 | AYTYVSEYKRLPVYNFGIa | 30.64 | 2181.126 | 0.3 | 728.0494 | 3 | 72.56 | 3 | Amidation |
| PFs | Q06601.1 | ADYPLRLNLD | 46.77 | 1188.614 | 0 | 595.3142 | 2 | 56.69 | 6 |  |
| PFs | Q06601.1 | YPLRLNLD | 42.12 | 1002.55 | 0.3 | 502.2823 | 2 | 49.35 | 12 |  |
| PFs | Q06601.1 | RQYSFGLa | 30.29 | 868.4555 | -0.2 | 435.235 | 2 | 26.45 | 19 | Amidation |
| PFs | Q06601.1 | GRQPYSFGLa | 34.37 | 1022.53 | 0.3 | 512.2723 | 2 | 31.51 | 5 | Amidation |
| PFs | Q06601.1 | GRDYSFGLa | 28.95 | 912.4453 | 0.2 | 457.23 | 2 | 30.84 | 8 | Amidation |
| PFs | Q06601.1 | WIDTNDNKRGRDYSFGLa | 24.14 | 2054.992 | 0.4 | 686.0049 | 3 | 38.84 | 3 | Amidation |
| PFs | Q06601.1 | LDYLPVDNPAFH | 40.17 | 1399.677 | -0.4 | 700.8456 | 2 | 67.71 | 7 |  |
| PFs | Q06601.1 | AVHYSGGQPLGS | 39.1 | 1171.562 | 0.2 | 586.7885 | 2 | 14.58 | 6 |  |
| PFs | Q06602.1 | EAEPEAEPGNNRPVYIPQPRPPHPRL | 23.07 | 2959.505 | 0.1 | 592.9084 | 5 | 39.74 | 5 |  |
| PFs | Q5DW47.1 | STSLEELANR | 28.15 | 1118.557 | 0.3 | 560.2858 | 2 | 25.81 | 5 |  |
| PFs | Q5DW47.1 | STSLEELANRN | 26.91 | 1232.6 | -0.5 | 617.3068 | 2 | 24.93 | 3 |  |
| PFs | Q5DW47.1 | pQTFTYSHGWTNa | 22.74 | 1322.568 | 0.6 | 662.2916 | 2 | 52.52 | 6 | Pyro-glu from Q; Amidation |
| PFs | Q868G6.1 | ASFDDEYYKRAPMGFQGMRa | 42.06 | 2267.025 | 0.6 | 567.7639 | 4 | 49.19 | 22 | Amidation |
| PFs | Q868G6.1 | VLSMDGYQNILDKKDELLGEWE | 41.48 | 2594.257 | -0.4 | 865.7594 | 3 | 98.46 | 12 |  |
| PFs | Q868G6.1 | VLSMDGYQNILD | 40.43 | 1366.644 | -0.3 | 684.329 | 2 | 82.61 | 11 |  |
| PFs | Q868G6.1 | GVMDFQIGLQ | 40.09 | 1106.543 | -0.7 | 554.2784 | 2 | 87.57 | 16 |  |
| PFs | Q868G6.1 | APMGFQGMRG | 38.9 | 1050.474 | -0.5 | 526.244 | 2 | 24.95 | 13 |  |
| PFs | Q868G6.1 | APMGFQGMRa | 38.73 | 992.4684 | -0.7 | 497.2411 | 2 | 20.02 | 38 | Amidation |
| PFs | Q868G6.1 | APMGFYGTRG | 38.3 | 1055.486 | -0.9 | 528.7497 | 2 | 21.9 | 13 |  |
| PFs | Q868G6.1 | ARMGFHGMRa | 37.1 | 1060.517 | 0 | 531.2658 | 2 | 9 | 18 | Amidation |
| PFs | Q868G6.1 | APMGFYGTRa | 37.08 | 997.4803 | -1.1 | 499.7469 | 2 | 18.43 | 23 | Amidation |
| PFs | Q868G6.1 | ALMGFQGVRa | 36.88 | 976.5276 | -0.7 | 489.2708 | 2 | 31.91 | 23 | Amidation |
| PFs | Q868G6.1 | ALMGFQGVRG | 36.33 | 1034.533 | 0.2 | 518.2739 | 2 | 38.06 | 10 |  |
| PFs | Q868G6.1 | SPFRYLGA | 35.81 | 909.4708 | -0.3 | 455.7426 | 2 | 40.27 | 14 |  |
| PFs | Q868G6.1 | GVMDFQIGLQRKKD | 34.42 | 1633.861 | 0.6 | 545.6279 | 3 | 39.49 | 8 |  |
| PFs | Q868G6.1 | SPFRYLGARG | 34.3 | 1122.593 | -0.5 | 375.2049 | 3 | 23.99 | 7 |  |
| PFs | Q868G6.1 | SPFRYLGARa | 33.85 | 1064.588 | 0 | 533.3012 | 2 | 19.83 | 7 | Amidation |
| PFs | Q868G6.1 | SLEEILDEIK | 33.49 | 1187.629 | 0.1 | 594.8216 | 2 | 93.96 | 13 |  |
| PFs | Q868G6.1 | SPFRYLG | 30.94 | 838.4337 | 0.3 | 420.2242 | 2 | 36.16 | 10 |  |
| PFs | Q868G6.1 | NPRWEFRGKFVGVRa | 30.44 | 1745.959 | 0.5 | 437.4972 | 4 | 32.34 | 10 | Amidation |
| PFs | Q868G6.1 | NPRWEFRGKFVGV | 30.3 | 1590.842 | 0.3 | 531.2881 | 3 | 49.83 | 3 |  |
| PFs | Q868G6.1 | ASFDDEYY | 28.5 | 1008.371 | 0.1 | 505.1929 | 2 | 44.7 | 5 |  |
| PFs | Q868G6.1 | SLEEILDEI | 25.89 | 1059.534 | 0.4 | 530.7743 | 2 | 108.72 | 6 |  |
| PFs | Q868G6.1 | IILDALEELD | 25.61 | 1142.607 | -0.2 | 572.3107 | 2 | 100.26 | 3 |  |
| PFs | XP_006557714.1 | pQQFDDYGHLRFa | 41.67 | 1406.637 | 0.5 | 704.326 | 2 | 69.79 | 13 | Pyro-glu from Q; Amidation |
| PFs | XP_006559359.1 | SVSSLAKNSAWPVSL | 46.58 | 1544.82 | -0.3 | 773.417 | 2 | 71.29 | 11 |  |
| PFs | XP_006559359.1 | NVASLARTYTLPQNAa | 44.1 | 1616.863 | -0.5 | 809.4386 | 2 | 46.96 | 8 | Amidation |
| PFs | XP_006559359.1 | FLLLPATDNNYFHQKLPSSLRSKSL | 42.63 | 2888.555 | 0.4 | 578.7184 | 5 | 77.31 | 22 |  |
| PFs | XP_006559359.1 | NVGSVAREHGLPYa | 41.93 | 1396.721 | -0.5 | 699.3674 | 2 | 24.98 | 21 | Amidation |
| PFs | XP_006559359.1 | YVASLARTGDLPIRGQ | 40.96 | 1715.932 | 0 | 572.9846 | 3 | 37.62 | 10 |  |
| PFs | XP_006559359.1 | HIGALARLGWLPSLRTA | 40.77 | 1831.058 | -0.1 | 611.3599 | 3 | 78.84 | 14 |  |
| PFs | XP_006559359.1 | NIASLMRDYDQSRENRVPFPa | 39.13 | 2406.186 | 0.9 | 803.0701 | 3 | 69.67 | 13 | Amidation |
| PFs | XP_006559359.1 | SVSSLARTGDLPVREQ | 38.63 | 1713.901 | -0.4 | 572.3073 | 3 | 27.58 | 8 |  |
| PFs | XP_006559359.1 | NVGTLARDFALPPa | 38.03 | 1368.751 | -0.6 | 685.3825 | 2 | 63.59 | 18 | Amidation |
| PFs | XP_006559359.1 | GIFLPGSVILRALSRQa | 37.1 | 1725.041 | 0 | 576.0211 | 3 | 98.8 | 12 | Amidation |
| PFs | XP_006559359.1 | YVASLARTGDLPIRa | 33.92 | 1529.868 | 0 | 510.9632 | 3 | 36.19 | 7 | Amidation |
| PFs | XP_006559359.1 | LPGSVILRALS | 28.72 | 1124.692 | 0.4 | 563.3533 | 2 | 76.05 | 10 |  |
| PFs | XP_006559359.1 | GIFLPGSVILR | 27.16 | 1170.712 | 1.5 | 586.3644 | 2 | 80.54 | 14 |  |
| PFs | XP_006559359.1 | HIGALARLGWLPSLRTARFS | 25.34 | 2221.26 | 0.4 | 556.3224 | 4 | 81.47 | 4 |  |
| PFs | XP_006559865.1 | AFGLLTYPRIa | 34.68 | 1148.671 | -0.1 | 575.3425 | 2 | 73.38 | 13 | Amidation |
| PFs | XP_006560385.1 | AYRKPPFNGSIFa | 37.99 | 1394.746 | -0.3 | 698.38 | 2 | 40.92 | 20 | Amidation |
| PFs | XP_006560385.1 | YRKPPFNGSIFa | 26.07 | 1323.709 | 0.1 | 662.8617 | 2 | 41.73 | 5 | Amidation |
| PFs | XP_006560385.1 | RKPPFNGSIFa | 24.15 | 1160.645 | 0.4 | 581.3302 | 2 | 33.49 | 11 | Amidation |
| PFs | XP_006562922.1 | GFKPEYISTAYGFa | 40.49 | 1477.724 | 0 | 739.8693 | 2 | 66.76 | 9 | Amidation |
| PFs | XP_006565207.1 | SDPHLSILSKPMSAIPSYKFDD | 45.28 | 2447.204 | -0.6 | 816.7415 | 3 | 75.81 | 11 |  |
| PFs | XP_006565207.1 | SPSLRLRFa | 30.48 | 973.5821 | -0.3 | 487.7982 | 2 | 28.72 | 19 | Amidation |
| PFs | XP_006565207.1 | SDPHLSILS | 27.48 | 967.4974 | 0 | 484.756 | 2 | 36.83 | 8 |  |
| PFs | XP_006565207.1 | SQRSPSLRLRFa | 24.3 | 1344.774 | 0.2 | 337.2008 | 4 | 20.45 | 5 | Amidation |
| PFs | XP_006570344.1 | NSELINSLLGLPKNMNNAa | 46.64 | 1940.015 | 0.2 | 971.015 | 2 | 91.06 | 12 | Amidation |
| PFs | XP_006570344.1 | LINSLLGLPKNMNNAa | 39.96 | 1609.897 | -0.2 | 805.9557 | 2 | 65.66 | 10 | Amidation |
| PFs | XP_016769998.1 | LVDHRIPDLENEMFDSGNDPGSTVVRT | 45.09 | 3012.425 | -0.7 | 754.1129 | 4 | 66.6 | 23 |  |
| NFs | Q868G6.1 | NSIINDVKNELFPEDIN | 71.58 | 1972.974 | -0.7 | 987.4937 | 2 | 96.19 | 11 |  |
| NFs | Q868G6.1 | VLSMDGYQNILDKKDELLGEWE | 58.61 | 2594.257 | 5.3 | 1298.143 | 2 | 95.2 | 4 |  |
| NFs | A8CL69.1 | TSQDITSGMWFGPRLa | 41.46 | 1693.825 | 1.1 | 847.9205 | 2 | 75.51 | 4 | Amidation |
| NFs | A8CL69.1 | pQLHNIVDKPRQNFNDPRF | 38.15 | 2220.119 | -1.1 | 556.0364 | 4 | 40.26 | 9 | Pyro-glu from Q |
| NFs | A8CL69.1 | pQITQFTPRLa | 18.4 | 1084.603 | 2 | 543.3098 | 2 | 65.6 | 8 | Pyro-glu from Q; Amidation |
| NFs | A8CL69.1 | GMWFGPRLa | 36.12 | 961.4956 | 1.3 | 481.7557 | 2 | 50.46 | 11 | Amidation |
| NFs | A8CL69.1 | MWFGPRLa | 26.19 | 904.4741 | 0.9 | 453.2448 | 2 | 53.31 | 9 | Amidation |
| NFs | A8CL69.1 | pQLHNIVDKPRQN | 50.72 | 1443.758 | 0.9 | 482.2604 | 3 | 14.04 | 8 | Pyro-glu from Q |
| NFs | A8CL69.1 | pQLHNIVDKP | 45.19 | 1045.556 | 0.8 | 523.7855 | 2 | 23.94 | 9 | Pyro-glu from Q |
| NFs | A8CL69.1 | RVPWTPSPRLa | 41.57 | 1206.699 | -0.9 | 604.356 | 2 | 23.89 | 5 | Amidation |
| NFs | ACI90290.1 | TWKSPDIVIRFa | 51.85 | 1359.766 | -0.7 | 454.2624 | 3 | 56.58 | 5 | Amidation |
| NFs | ACI90290.1 | GRNDLNFIRYa | 36.79 | 1265.663 | 0.2 | 633.8388 | 2 | 30.39 | 9 | Amidation |
| NFs | ACI90290.1 | QITQFTPRLa | 33.64 | 1101.63 | -0.4 | 551.8218 | 2 | 32.4 | 10 | Amidation |
| NFs | ACI90290.1 | AGFKNLNREQ | 36.45 | 1175.605 | -0.1 | 588.8096 | 2 | 10.17 | 12 |  |
| NFs | ACI90290.1 | SPDIVIRFa | 33.7 | 944.5443 | -0.9 | 473.279 | 2 | 45.58 | 8 | Amidation |
| NFs | NP_001161192.1 | PEIFTSPEELRRYIDHVSDYYLLSGKARYa | 46.23 | 3515.784 | 0.6 | 586.9714 | 6 | 94.48 | 7 | Amidation |
| NFs | P85527.1 | pQDVDHVFLRFa | 52.69 | 1256.63 | 0 | 629.3223 | 2 | 72.23 | 6 | Pyro-glu from Q; Amidation |
| NFs | P85527.1 | QDVDHVFLRFa | 48.09 | 1273.657 | 0.3 | 425.5596 | 3 | 48.21 | 5 | Amidation |
| NFs | P85527.1 | pQDVDHVFLRF | 49.46 | 1257.614 | 0.2 | 629.8145 | 2 | 74.75 | 6 | Pyro-glu from Q |
| NFs | P85527.1 | pQDVDHVFLR | 43.61 | 1110.546 | 2.4 | 556.2815 | 2 | 30.9 | 8 | Pyro-glu from Q |
| NFs | P85527.1 | pQDVDHVFL | 24.41 | 954.4447 | 0.5 | 478.2299 | 2 | 70.18 | 6 | Pyro-glu from Q |
| NFs | P85798.1 | LRNQLDIGDLQ | 49.85 | 1283.683 | 0 | 642.8489 | 2 | 41.44 | 6 |  |
| NFs | P85798.1 | LRNQLDIGDL | 46.57 | 1155.625 | 0.7 | 578.8201 | 2 | 35.03 | 3 |  |
| NFs | P85798.1 | IPAADKERLLN | 48.73 | 1238.698 | 0.9 | 620.3569 | 2 | 15.09 | 7 |  |
| NFs | P85799.1 | SQAYDPYSNAAQFQLSSQSRGYPYQHRLVY | 76.59 | 3523.655 | -0.9 | 881.9201 | 4 | 60.11 | 31 |  |
| NFs | P85799.1 | SQAYDPYSNAAQFQLSSQSRGYPYQHRL | 66.47 | 3261.523 | -1.1 | 816.3871 | 4 | 51.7 | 6 |  |
| NFs | P85799.1 | SQAYDPYSNAAQFQLSSQSRGYPYQHRLV | 62.02 | 3360.591 | -2 | 841.1534 | 4 | 55.98 | 5 |  |
| NFs | P85799.1 | LPTNLAEDTKKTEQTMRPKS | 60.38 | 2287.184 | -0.6 | 572.803 | 4 | 20.72 | 19 |  |
| NFs | P85799.1 | NVPIYQEPRF | 46.81 | 1261.646 | -0.8 | 631.8295 | 2 | 45.2 | 8 |  |
| NFs | P85799.1 | VPIYQEPRF | 45.86 | 1147.603 | -0.2 | 574.8084 | 2 | 42.06 | 7 |  |
| NFs | P85799.1 | GYPYQHRLVY | 39.06 | 1294.646 | -0.3 | 648.33 | 2 | 23.28 | 7 |  |
| NFs | P85799.1 | PIYQEPRF | 25.88 | 1048.534 | 0.2 | 525.2745 | 2 | 45.14 | 3 |  |
| NFs | P85828.1 | ITGQGNRIF | 48.34 | 1004.54 | 0.4 | 503.2776 | 2 | 19.51 | 3 |  |
| NFs | P85828.1 | SLKAPFA | 40.92 | 732.417 | 0.1 | 367.2158 | 2 | 29.4 | 5 |  |
| NFs | P85828.1 | SLKAPF | 35.22 | 661.3799 | -0.1 | 331.6972 | 2 | 23.98 | 5 |  |
| NFs | P85829.1 | MVPVPVHHMADELLRNGPDTVI | 49.4 | 2439.24 | 0.1 | 814.0874 | 3 | 74.93 | 6 |  |
| NFs | P85829.1 | VHHMADELLRNGPDTVI | 42.7 | 1915.957 | -0.5 | 639.6594 | 3 | 44.62 | 5 |  |
| NFs | P85829.1 | VPVPVHHMADELL | 49.99 | 1455.754 | 0.2 | 728.8846 | 2 | 33.13 | 7 |  |
| NFs | P85829.1 | LLRNGPDTVI | 35.24 | 1096.624 | -0.1 | 549.3192 | 2 | 28.76 | 11 |  |
| NFs | P85829.1 | LRNGPDTVI | 28.81 | 983.54 | 0.5 | 492.7775 | 2 | 16.64 | 12 |  |
| NFs | P85830.1 | GLDLGLSRGFSGSQAAKHLMGLAAANYAGGPa | 75.12 | 2985.524 | 0.3 | 996.1823 | 3 | 83.37 | 8 | Amidation |
| NFs | P85830.1 | GLDLGLSRGFSGSQAAKH | 47.59 | 1799.928 | -0.2 | 600.9831 | 3 | 30.26 | 7 |  |
| NFs | P85830.1 | GLDLGLSRGFSGSQAAKHLMa | 34.44 | 2043.068 | 1.2 | 682.0309 | 3 | 54.93 | 8 | Amidation |
| NFs | P85830.1 | GLDLGLSRGFSGSQAA | 70.04 | 1534.774 | 0.5 | 768.3947 | 2 | 43.37 | 5 |  |
| NFs | P85830.1 | HLMGLAAANYAGGPa | 47.07 | 1340.666 | -0.8 | 671.3397 | 2 | 35.45 | 8 | Amidation |
| NFs | P85831.1 | IDLSRFYGHFNT | 66.33 | 1468.71 | -0.8 | 735.3616 | 2 | 62.1 | 15 |  |
| NFs | P85831.1 | IDLSRFYGHFN | 59.58 | 1367.662 | 0 | 684.8384 | 2 | 59.48 | 12 |  |
| NFs | P85831.1 | IDLSRFYGHF | 54.45 | 1253.619 | 0.1 | 627.817 | 2 | 66.68 | 7 |  |
| NFs | P85831.1 | IDLSRFYGHFNTKR | 45.5 | 1752.906 | 0.5 | 585.3095 | 3 | 39.93 | 6 |  |
| NFs | P85831.1 | DLSRFYGHFN | 33.5 | 1254.578 | -0.6 | 628.8198 | 2 | 65.09 | 14 |  |
| NFs | P85831.1 | FYGHFNT | 44.01 | 884.3817 | -0.2 | 443.1981 | 2 | 20.92 | 13 |  |
| NFs | P85832.1 | LTNYLATTGHGTNTGGPVLT | 85.24 | 1987.001 | -0.6 | 994.5072 | 2 | 47.09 | 8 |  |
| NFs | P85832.1 | NLDEIDRVGWSGFV | 56.68 | 1605.779 | -0.9 | 803.8959 | 2 | 86.63 | 4 |  |
| NFs | P85832.1 | NIDEIDRTAFDNFF | 54.74 | 1715.779 | -0.7 | 858.8962 | 2 | 94.63 | 7 |  |
| NFs | P85832.1 | LTNYLATTGHGTNTGGPVLTRRFa | 52.84 | 2445.288 | 0 | 612.3292 | 4 | 38.23 | 12 | Amidation |
| NFs | P85832.1 | ELVDELSPVSERETLERFa | 29.85 | 2146.091 | -1.2 | 716.3699 | 3 | 72.71 | 4 | Amidation |
| NFs | P85832.1 | LVDELSPVSERETLERFa | 26.21 | 2017.048 | -1.1 | 673.3558 | 3 | 61.74 | 3 | Amidation |
| NFs | P85832.1 | LTNYLATTGHGTNTGGPVL | 83.65 | 1885.953 | 3.4 | 943.9872 | 2 | 37.57 | 3 |  |
| NFs | Q06601.1 | PNDMLSQRYHFGLa | 69.72 | 1575.762 | -0.4 | 526.2609 | 3 | 47.76 | 6 | Amidation |
| NFs | Q06601.1 | AVHYSGGQPLGSKRPNDMLSQRYHFGLa | 66.38 | 3013.509 | 0.1 | 754.3846 | 3 | 41.05 | 10 | Amidation |
| NFs | Q06601.1 | AYTYVSEYKRLPVYNFGIa | 69.78 | 2181.126 | -1.2 | 1091.569 | 2 | 73.7 | 8 | Amidation |
| NFs | Q06601.1 | ADYPLRLNLD | 50.74 | 1188.614 | 0.6 | 595.3146 | 2 | 57.13 | 15 |  |
| NFs | Q06601.1 | YPLRLNLD | 44.94 | 1002.55 | 0.3 | 502.2823 | 2 | 49.7 | 21 |  |
| NFs | Q06601.1 | LDYLPVDNPAFH | 54.33 | 1399.677 | 1.6 | 700.8469 | 2 | 61.58 | 5 |  |
| NFs | Q06601.1 | RQYSFGLa | 34.76 | 868.4555 | 0.1 | 435.2351 | 2 | 23.68 | 7 | Amidation |
| NFs | Q06601.1 | GRQPYSFGLa | 38.89 | 1022.53 | 0.2 | 512.2722 | 2 | 29.28 | 7 | Amidation |
| NFs | Q06601.1 | GRDYSFGLa | 30.15 | 912.4453 | 0 | 457.2299 | 2 | 29.2 | 10 | Amidation |
| NFs | Q06601.1 | WIDTNDNKRGRDYSFGLa | 36.47 | 2054.992 | 1 | 686.0054 | 3 | 34.51 | 7 | Amidation |
| NFs | Q06601.1 | AVHYSGGQPLGS | 39.03 | 1171.562 | 0.3 | 586.7885 | 2 | 13.57 | 11 |  |
| NFs | Q06602.1 | EAEPEAEPGNNRPVYIPQPRPPHPRL | 66.9 | 2959.505 | 3.2 | 592.9102 | 5 | 21.55 | 23 |  |
| NFs | Q5DW47.1 | STSLEELANR | 36.25 | 1118.557 | 0.9 | 560.2861 | 2 | 24.18 | 7 |  |
| NFs | Q5DW47.1 | STSLEELANRN | 39.72 | 1232.6 | 0.7 | 617.3075 | 2 | 23.07 | 9 |  |
| NFs | Q5DW47.1 | pQTFTYSHGWTNa | 51.7 | 1322.568 | -0.6 | 662.2909 | 2 | 50.66 | 19 | Pyro-glu from Q; Amidation |
| NFs | Q868G6.1 | ASFDDEYYKRAPMGFQGMRa | 58.13 | 2267.025 | -0.1 | 567.7635 | 4 | 43.53 | 9 | Amidation |
| NFs | Q868G6.1 | ARMGFHGMRa | 54.74 | 1060.517 | -0.5 | 531.2656 | 2 | 8.87 | 7 | Amidation |
| NFs | Q868G6.1 | APMGFQGMRa | 49.97 | 992.4684 | 0 | 497.2415 | 2 | 19.16 | 6 | Amidation |
| NFs | Q868G6.1 | SLEEILDEIK | 47.34 | 1187.629 | 0.3 | 594.8217 | 2 | 84.58 | 9 |  |
| NFs | Q868G6.1 | NPRWEFRGKFVGV | 45.98 | 1590.842 | 0.3 | 531.2881 | 3 | 40.1 | 9 |  |
| NFs | Q868G6.1 | SPFRYLGARa | 42.47 | 1064.588 | 0.1 | 533.3013 | 2 | 15.98 | 4 | Amidation |
| NFs | Q868G6.1 | ALMGFQGVRa | 42.22 | 976.5276 | -0.3 | 489.2709 | 2 | 29.18 | 6 | Amidation |
| NFs | Q868G6.1 | NPRWEFRGKFVGVRa | 40.99 | 1745.959 | -0.2 | 437.4969 | 4 | 21.41 | 7 | Amidation |
| NFs | Q868G6.1 | SPFRYLGA | 40.83 | 909.4708 | 0 | 455.7427 | 2 | 35.07 | 7 |  |
| NFs | Q868G6.1 | GVMDFQIGLQRKKD | 40.37 | 1633.861 | 0.1 | 545.6276 | 3 | 34.17 | 7 |  |
| NFs | Q868G6.1 | SPFRYLGARG | 39.19 | 1122.593 | -0.4 | 375.2049 | 3 | 19.38 | 4 |  |
| NFs | Q868G6.1 | ALMGFQGVRG | 38.17 | 1034.533 | -0.1 | 518.2737 | 2 | 34.43 | 13 |  |
| NFs | Q868G6.1 | GVMDFQIGLQ | 37.68 | 1106.543 | 0.3 | 554.2789 | 2 | 84.25 | 6 |  |
| NFs | Q868G6.1 | VLSMDGYQNILD | 36.56 | 1366.644 | 0.1 | 684.3292 | 2 | 79.28 | 3 |  |
| NFs | Q868G6.1 | APMGFYGTRa | 36.12 | 997.4803 | 1.1 | 499.748 | 2 | 16.97 | 3 | Amidation |
| NFs | Q868G6.1 | ASFDDEYY | 30.67 | 1008.371 | 0.1 | 505.1929 | 2 | 40.98 | 4 |  |
| NFs | Q868G6.1 | SLEEILDEI | 23.62 | 1059.534 | -1.5 | 530.7733 | 2 | 104.74 | 3 |  |
| NFs | Q868G6.1 | IILDALEELD | 28 | 1142.607 | 0.6 | 572.3112 | 2 | 102.74 | 5 |  |
| NFs | Q868G6.1 | APMGFQGMRG | 50.06 | 1050.474 | -0.3 | 526.2441 | 2 | 23.14 | 5 |  |
| NFs | Q868G6.1 | APMGFYGTRG | 43.78 | 1055.486 | -0.3 | 528.7501 | 2 | 20.72 | 3 |  |
| NFs | XP_006557714.1 | pQQFDDYGHLRFa | 59.46 | 1406.637 | 0.4 | 704.3259 | 2 | 41.18 | 4 | Pyro-glu from Q; Amidation |
| NFs | XP_006559359.1 | SVSSLAKNSAWPVSL | 70.04 | 1544.82 | 0 | 773.4172 | 2 | 66.98 | 6 |  |
| NFs | XP_006559359.1 | NVASLARTYTLPQNAa | 66.96 | 1616.863 | -0.3 | 809.4387 | 2 | 42.45 | 6 | Amidation |
| NFs | XP_006559359.1 | NVGSVAREHGLPYa | 62.66 | 1396.721 | -0.5 | 699.3675 | 2 | 20.47 | 11 | Amidation |
| NFs | XP_006559359.1 | YVASLARTGDLPIRGQ | 57.46 | 1715.932 | 0.2 | 572.9846 | 3 | 35.02 | 9 |  |
| NFs | XP_006559359.1 | FLLLPATDNNYFHQKLPSSLRSKSL | 51.46 | 2888.555 | 1.1 | 578.7189 | 5 | 69.14 | 13 |  |
| NFs | XP_006559359.1 | SVSSLARTGDLPVREQ | 47.87 | 1713.901 | 0 | 572.3076 | 3 | 24.99 | 6 |  |
| NFs | XP_006559359.1 | NIASLMRDYDQSRENRVPFPa | 45.43 | 2406.186 | -0.5 | 803.069 | 3 | 60.51 | 5 | Amidation |
| NFs | XP_006559359.1 | YVASLARTGDLPIRa | 35.3 | 1529.868 | 0.8 | 510.9636 | 3 | 31.89 | 4 | Amidation |
| NFs | XP_006559359.1 | LPGSVILRALS | 34.9 | 1124.692 | 0.5 | 563.3534 | 2 | 70.13 | 8 |  |
| NFs | XP_006559359.1 | NVGTLARDFALPPa | 33.49 | 1368.751 | 0.1 | 685.383 | 2 | 59.01 | 11 | Amidation |
| NFs | XP_006559359.1 | GIFLPGSVILR | 32.7 | 1170.712 | 0.2 | 586.3636 | 2 | 76.91 | 5 |  |
| NFs | XP_006559359.1 | GIFLPGSVILRALSRQa | 31.65 | 1725.041 | -1 | 576.0204 | 3 | 94.65 | 8 | Amidation |
| NFs | XP_006559359.1 | HIGALARLGWLPSLRTARFS | 29.69 | 2221.26 | -0.6 | 556.3218 | 4 | 68.04 | 3 |  |
| NFs | XP_006559359.1 | HIGALARLGWLPSLRTA | 24.36 | 1831.058 | 0.4 | 611.3602 | 3 | 67.47 | 5 |  |
| NFs | XP_006559359.1 | NVGTLARDFALPPGRRNIASLMRDYDQSRENRVPFPa | 19.42 | 4127.135 | 0.3 | 688.8633 | 6 | 72.55 | 6 | Amidation |
| NFs | XP_006559865.1 | AFGLLTYPRIa | 34 | 1148.671 | -0.2 | 575.3424 | 2 | 68.55 | 5 | Amidation |
| NFs | XP_006559865.1 | EKLKPNMRRAFGLLTYPRIa | 21.02 | 2301.325 | 0.5 | 576.3389 | 4 | 45.4 | 4 | Amidation |
| NFs | XP_006560385.1 | AYRKPPFNGSIFa | 41.64 | 1394.746 | -0.1 | 698.3801 | 2 | 35.3 | 6 | Amidation |
| NFs | XP_006560385.1 | KPPFNGSIFa | 36.34 | 1004.544 | 0 | 503.2794 | 2 | 42.18 | 7 | Amidation |
| NFs | XP_006560385.1 | YRKPPFNGSIFa | 47.47 | 1323.709 | 0.3 | 662.8619 | 2 | 32.59 | 7 | Amidation |
| NFs | XP_006560385.1 | RKPPFNGSIFa | 40.45 | 1160.645 | -0.2 | 581.3298 | 2 | 24.42 | 9 | Amidation |
| NFs | XP_006562922.1 | GFKPEYISTAYGFa | 52.25 | 1477.724 | 0.6 | 739.8698 | 2 | 70.58 | 29 | Amidation |
| NFs | XP_006565207.1 | SDPHLSILSKPMSAIPSYKFDD | 72.01 | 2447.204 | -0.3 | 816.7418 | 3 | 70.27 | 6 |  |
| NFs | XP_006565207.1 | SQRSPSLRLRFa | 32.44 | 1344.774 | -0.2 | 449.2651 | 3 | 15.82 | 6 | Amidation |
| NFs | XP_006565207.1 | SPSLRLRFa | 28.98 | 973.5821 | 0.5 | 487.7986 | 2 | 22.89 | 5 | Amidation |
| NFs | XP_006565207.1 | SDPHLSILS | 38.23 | 967.4974 | 0 | 484.756 | 2 | 33.67 | 7 |  |
| NFs | XP_006570344.1 | NSELINSLLGLPKNMNNAa | 64.9 | 1940.015 | 3.8 | 971.0184 | 2 | 85.93 | 7 | Amidation |
| NFs | XP_006570344.1 | LINSLLGLPKNMNNAa | 54.36 | 1609.897 | -0.3 | 805.9557 | 2 | 61.69 | 6 | Amidation |
| NFs | XP_016769998.1 | LVDHRIPDLENEMFDSGNDPGSTVVRT | 65.72 | 3012.425 | -0.4 | 1005.148 | 3 | 63.14 | 13 |  |
| NFs | XP_016769998.1 | IGSLSIVNSMDVLRQRVLLELARRKALQDQAQIDANRRLLETIa | 34.17 | 4913.782 | -0.5 | 819.9706 | 6 | 96.85 | 12 | Amidation |
| NFs | XP_016769998.1 | HPISYNTYDERELSRDHPPLLL | 33.16 | 2664.33 | 0.3 | 667.0898 | 4 | 52.44 | 6 |  |
