# Supplementary material for: Tachykinin signaling inhibits task-specific behavioral responsiveness in honeybee workers
Source: eLife. 2021 Mar 24;10:e64830. doi: 10.7554/eLife.64830 (PMC8016481; doi:10.7554/eLife.64830)
Supplement: Supplementary file 3. [file elife-64830-supp3.docx]

Neuropeptides identified in the brain of *Apis cerana cerana* workers. (manuscript section 2.2)

"**NBs**": nurse bees. "**PFs**": pollen foragers. "**NFs**": nectar foragers. "**Protein Accession**": the unique number given to mark the entry of a protein in the database NCBInr. "**Peptide**": the amino acid sequence of the peptide as determined in PEAKS Search. "**-10lgP**": the score indicates the scoring significance of a peptide-spectrum match. "**Mass**": the monoisotopic mass of the peptide. "**ppm**": the precursor mass error, calculated as 10^6^ × (precursor mass - peptide mass) / peptide mass. "**m/z**": the precursor mass-to-charge ratio. "**z**": the peptide charge. "**RT**": the retention time (elution time) of the spectrum as recorded in the data. "**#Spec**": the number of scanned spectrums of the peptide. "**PTM**": the post translational modification types present in the peptide.

| **Sample** | **Protein Accession** | **Peptide** | **-10lgP** | **Mass** | **ppm** | **m/z** | **z** | **RT** | **#Spec** | **PTM** |
| --- | --- | --- | --- | --- | --- | --- | --- | --- | --- | --- |
| NBs | PBC25365.1 | pQQFDDYGHLRFa | 26.97 | 1406.637 | -2 | 704.3242 | 2 | 56.29 | 6 | Pyro-glu from Q; Amidation |
| NBs | PBC27532.1 | LVDHRIPDLENEMF | 48.92 | 1726.835 | 1.8 | 864.4263 | 2 | 49.73 | 8 |  |
| NBs | PBC27532.1 | ISYDTYDERELSRDHPPLLL | 47.44 | 2431.202 | 2 | 811.4095 | 3 | 51.01 | 9 |  |
| NBs | PBC27532.1 | HPISYDTYDERELSRDHPPLLL | 45.5 | 2665.314 | 0.7 | 889.4457 | 3 | 41.65 | 14 |  |
| NBs | PBC27532.1 | SLPLYGGNMSKTGDSRLKSE | 45.37 | 2139.063 | 1 | 535.7736 | 4 | 19.63 | 8 |  |
| NBs | PBC27532.1 | SLPLYGGNMSKTGDSRLKSEFE | 43.99 | 2415.174 | 1.1 | 806.0662 | 3 | 30.88 | 7 |  |
| NBs | PBC27532.1 | IGSLSIVNSMDVLRQRVLLELARRKALQDQAQIDANRRLLETIa | 41.71 | 4913.782 | 0.6 | 983.7643 | 5 | 87.07 | 14 | Amidation |
| NBs | PBC27532.1 | ARRKALQDQAQIDANRRLLETIa | 37.21 | 2577.458 | 0.4 | 516.499 | 5 | 22.74 | 4 | Amidation |
| NBs | PBC27532.1 | LVDHRIPDLENEMFDSGNDPGSTVVRT | 58.45 | 3012.425 | 0.6 | 1005.149 | 3 | 50.38 | 18 |  |
| NBs | PBC27982.1 | ITGQGNRIF | 39.25 | 1004.54 | 0.5 | 503.2777 | 2 | 18.96 | 8 |  |
| NBs | PBC27982.1 | SLKAPFA | 34.7 | 732.417 | -0.5 | 367.2156 | 2 | 20.05 | 5 |  |
| NBs | PBC27985.1 | YLLSGKARYa | 31.25 | 1068.608 | 0.7 | 535.3116 | 2 | 11.7 | 5 | Amidation |
| NBs | PBC28057.1 | GNNRPVYIPQPRPPHP | 45.28 | 1837.97 | 0.8 | 613.6644 | 3 | 17.05 | 10 |  |
| NBs | PBC28057.1 | GNNRPVYIPQPRPPHPRL | 38.71 | 2107.155 | 1.9 | 703.3936 | 3 | 19.33 | 10 |  |
| NBs | PBC28057.1 | PVYIPQPRPPHP | 36.58 | 1396.762 | 0 | 466.5944 | 3 | 22.15 | 3 |  |
| NBs | PBC28214.1 | GLDLGLSRGFSGSQAAKHLMGLAAANYAGGPa | 55.91 | 2985.524 | 2.3 | 996.1843 | 3 | 69.28 | 15 | Amidation |
| NBs | PBC28214.1 | GLDLGLSRGFSGSQAAKH | 43.4 | 1799.928 | 0.6 | 900.9717 | 2 | 23.14 | 9 |  |
| NBs | PBC28214.1 | GLDLGLSRGFSGSQAA | 51.79 | 1534.774 | 1.7 | 768.3955 | 2 | 42.16 | 6 |  |
| NBs | PBC28214.1 | GLDLGLSRGFSGSQAAKHLMa | 37.61 | 2043.068 | 0.8 | 682.0306 | 3 | 41.21 | 3 | Amidation |
| NBs | PBC30406.1 | SDPHLSIGILSKPISAIPSSKFDD | 54.85 | 2523.322 | 1.1 | 842.1155 | 3 | 58.84 | 15 |  |
| NBs | PBC30406.1 | SPSLRLRFa | 33.83 | 973.5821 | 0.1 | 487.7984 | 2 | 18.81 | 4 | Amidation |
| NBs | PBC30406.1 | SDPHLSIGILSKPISAIP | 32.67 | 1844.041 | 1.8 | 615.6886 | 3 | 64.64 | 5 |  |
| NBs | PBC30406.1 | SQRSPSLRLRFa | 30.49 | 1344.774 | -0.2 | 449.2651 | 3 | 14.25 | 3 | Amidation |
| NBs | PBC30406.1 | SDPHLSIGILSKP | 47.29 | 1362.751 | 1.1 | 682.3834 | 2 | 31.95 | 8 |  |
| NBs | PBC31004.1 | pQMFTYSHGWTNa | 36.09 | 1352.561 | 1.4 | 677.2886 | 2 | 54.66 | 3 | Pyro-glu from Q; Amidation |
| NBs | PBC31004.1 | STSLEELVNR | 32.15 | 1146.588 | 0.5 | 574.3016 | 2 | 27.94 | 3 |  |
| NBs | PBC31251.1 | YRKPPFNGSIFa | 37.89 | 1323.709 | 0.8 | 662.8622 | 2 | 24.67 | 4 | Amidation |
| NBs | PBC31251.1 | AYRKPPFNGSIFa | 36.33 | 1394.746 | 1.3 | 698.3811 | 2 | 25.09 | 13 | Amidation |
| NBs | PBC31251.1 | KPPFNGSIFa | 27.98 | 1004.544 | 0.7 | 503.2798 | 2 | 30.98 | 5 | Amidation |
| NBs | PBC31251.1 | RKPPFNGSIFa | 20.29 | 1160.645 | 1 | 581.3306 | 2 | 21.64 | 12 | Amidation |
| NBs | PBC31431.1 | APVGYQEMQGKKNSASLNSENFGIF | 54.25 | 2715.296 | 2.7 | 906.1084 | 3 | 48.51 | 8 |  |
| NBs | PBC31431.1 | NSIINDVKNELFPEDIN | 51.1 | 1972.974 | 0.9 | 987.4952 | 2 | 83.93 | 25 |  |
| NBs | PBC31431.1 | STDFQDVESGSESFKRARMGFHGMRa | 45.17 | 2860.313 | 0.4 | 573.0701 | 5 | 25.77 | 7 | Amidation |
| NBs | PBC31431.1 | ARMGFHGMRa | 43.34 | 1060.517 | 1 | 531.2664 | 2 | 7.55 | 19 | Amidation |
| NBs | PBC31431.1 | APMGFQGMRG | 41.6 | 1050.474 | 1 | 526.2448 | 2 | 19.99 | 4 |  |
| NBs | PBC31431.1 | SPFRYLGV | 41.02 | 937.5021 | 0.2 | 469.7584 | 2 | 36.47 | 9 |  |
| NBs | PBC31431.1 | APMGFYGTRG | 40.33 | 1055.486 | 0.8 | 528.7506 | 2 | 18.81 | 3 |  |
| NBs | PBC31431.1 | APMGFQGMRa | 40.14 | 992.4684 | 0.5 | 497.2418 | 2 | 17.14 | 9 | Amidation |
| NBs | PBC31431.1 | ALMGFQGVRG | 38.56 | 1034.533 | 1.2 | 518.2744 | 2 | 26.54 | 4 |  |
| NBs | PBC31431.1 | ALMGFQGVRa | 38.13 | 976.5276 | 0.8 | 489.2715 | 2 | 23.4 | 5 | Amidation |
| NBs | PBC31431.1 | APMGFYGTRa | 37.78 | 997.4803 | 0.3 | 499.7476 | 2 | 15.26 | 6 | Amidation |
| NBs | PBC31431.1 | ARMGFHGMRG | 36.45 | 1118.523 | -0.5 | 373.848 | 3 | 9.58 | 3 |  |
| NBs | PBC31431.1 | SPFRYLGVRa | 35.49 | 1092.619 | 0.3 | 547.3171 | 2 | 17.93 | 10 | Amidation |
| NBs | PBC31431.1 | ASFDDEYY | 22.86 | 1008.371 | 0.6 | 505.1932 | 2 | 33.37 | 7 |  |
| NBs | PBC31431.1 | ASFDDEYYKRAPMGFQGMRa | 50.81 | 2267.025 | 1.1 | 567.7642 | 4 | 31.82 | 6 | Amidation |
| NBs | PBC31431.1 | STDFQDVESGSESF | 45.52 | 1533.611 | 0.9 | 767.8133 | 2 | 45.31 | 8 |  |
| NBs | PBC32274.1 | pQLHNIIDKPRQN | 42.12 | 1457.774 | 1.2 | 729.8951 | 2 | 16.05 | 6 | Pyro-glu from Q |
| NBs | PBC32274.1 | RVPWTPSPRLa | 36.39 | 1206.699 | 1.2 | 604.3572 | 2 | 19.93 | 5 | Amidation |
| NBs | PBC32274.1 | pQLHNIIDKPRQNFNDPRF | 36.19 | 2234.135 | 0.3 | 559.5411 | 4 | 32.1 | 7 | Pyro-glu from Q |
| NBs | PBC32274.1 | pQITQFTPRLa | 33.03 | 1084.603 | 0.4 | 543.309 | 2 | 53.9 | 4 | Pyro-glu from Q; Amidation |
| NBs | PBC32274.1 | VPWTPSPRLa | 32.1 | 1050.597 | 0.2 | 526.3061 | 2 | 25.14 | 3 | Amidation |
| NBs | PBC32274.1 | pQLHNIIDKPRQNFNDP | 28.81 | 1930.965 | 1.6 | 966.4913 | 2 | 26.9 | 4 | Pyro-glu from Q |
| NBs | PBC32274.1 | SGMWFGPRLa | 27.1 | 1048.528 | 1.5 | 525.2719 | 2 | 49.17 | 3 | Amidation |
| NBs | PBC32274.1 | TSQDITSGMWFGPRLa | 42.69 | 1693.825 | 1.1 | 847.9205 | 2 | 63.45 | 10 | Amidation |
| NBs | PBC32274.1 | DITSGMWFGPRLa | 33.24 | 1377.686 | 1.6 | 689.8515 | 2 | 77.83 | 3 | Amidation |
| NBs | PBC32274.1 | pQLHNIIDKP | 32.19 | 1059.571 | -0.1 | 530.7928 | 2 | 24.84 | 5 | Pyro-glu from Q |
| NBs | PBC32274.1 | SQDITSGMWFGPRLa | 31.19 | 1592.777 | 2.4 | 797.3976 | 2 | 67.49 | 3 | Amidation |
| NBs | PBC32274.1 | GMWFGPRLa | 31.02 | 961.4956 | 0.7 | 481.7554 | 2 | 50.43 | 5 | Amidation |
| NBs | PBC32496.1 | IPAADKERLLN | 41.66 | 1238.698 | 0.7 | 620.3568 | 2 | 14.79 | 5 |  |
| NBs | PBC32496.1 | LRNQLDIGDLQ | 40.78 | 1283.683 | 2.1 | 642.8503 | 2 | 31.12 | 6 |  |
| NBs | PBC32496.1 | SYWKQCAFNAVSCFa | 39.16 | 1651.728 | 1.1 | 826.8719 | 2 | 69.48 | 5 | Amidation |
| NBs | PBC32545.1 | NSELINSLLGLPKNMNNAa | 46.62 | 1940.015 | 1.7 | 971.0164 | 2 | 72.76 | 8 | Amidation |
| NBs | PBC32608.1 | IDLSRFYGHFNTKR | 47.18 | 1752.906 | 1.2 | 585.3099 | 3 | 28.6 | 11 |  |
| NBs | PBC32608.1 | IDLSRFYGHFNT | 45.98 | 1468.71 | 1.8 | 735.3635 | 2 | 49.16 | 9 |  |
| NBs | PBC32608.1 | IDLSRFYGHF | 43.61 | 1253.619 | 0.9 | 627.8174 | 2 | 53.8 | 6 |  |
| NBs | PBC32608.1 | DLSRFYGHF | 26.84 | 1140.535 | 0.4 | 571.2751 | 2 | 36.46 | 7 |  |
| NBs | PBC32608.1 | IDLSRFYGHFNTK | 29.22 | 1596.805 | -0.1 | 533.2755 | 3 | 35.94 | 3 |  |
| NBs | PBC32678.1 | pQDVDHVFLRFa | 40.98 | 1256.63 | 0.8 | 629.3228 | 2 | 61.33 | 6 | Pyro-glu from Q; Amidation |
| NBs | PBC32678.1 | QDVDHVFLRFa | 40.61 | 1273.657 | 0.9 | 637.8362 | 2 | 36.74 | 8 | Amidation |
| NBs | PBC32678.1 | pQDVDHVFLR | 39.04 | 1110.546 | 1.2 | 556.2808 | 2 | 33.73 | 4 | Pyro-glu from Q |
| NBs | PBC32727.1 | LPTNLGEDTKKTEQTMRPKS | 49.08 | 2273.169 | 0.9 | 569.2999 | 4 | 15.3 | 14 |  |
| NBs | PBC32727.1 | SQAYDPYSNAAQFQLSSQSRGYPYQHRL | 48.8 | 3261.523 | 0.5 | 816.3884 | 4 | 39.12 | 7 |  |
| NBs | PBC32727.1 | NVPIYQEPRF | 35.7 | 1261.646 | 0.1 | 631.8301 | 2 | 32.71 | 5 |  |
| NBs | PBC32727.1 | YPYQHRLIY | 34.97 | 1251.64 | 1.2 | 626.8281 | 2 | 21.18 | 4 |  |
| NBs | PBC32727.1 | SQAYDPYSNAAQFQLSSQSRGYPYQHRLIY | 55.22 | 3537.67 | 1.8 | 885.4265 | 4 | 51.39 | 26 |  |
| NBs | PBC32727.1 | SQAYDPYSNAAQFQLSSQSRGYPYQHRLI | 51.38 | 3374.607 | 1.7 | 844.6604 | 4 | 46.46 | 5 |  |
| NBs | PBC32727.1 | VPIYQEPRF | 36.9 | 1147.603 | 0.8 | 574.809 | 2 | 30.77 | 3 |  |
| NBs | PBC32727.1 | GYPYQHRLIY | 27.89 | 1308.662 | 1.5 | 655.339 | 2 | 22.03 | 5 |  |
| NBs | PBC32914.1 | SIATLAKNDDLPISLHDRMAENEDDEE | 54.94 | 3040.393 | 0.8 | 1014.472 | 3 | 42.04 | 10 |  |
| NBs | PBC32914.1 | FLLLPATDNNYFHQKLPSSLRSKSL | 47.88 | 2888.555 | 1.7 | 578.7192 | 5 | 54.83 | 13 |  |
| NBs | PBC32914.1 | YVASLARTGDLPIRGQ | 44.56 | 1715.932 | 1 | 858.974 | 2 | 24.87 | 10 |  |
| NBs | PBC32914.1 | NVGSVAREHGLPYa | 43.9 | 1396.721 | 1 | 699.3685 | 2 | 17.05 | 11 | Amidation |
| NBs | PBC32914.1 | NIASLIRDYDQSRENRVSFPa | 40.86 | 2378.209 | 0.9 | 793.7443 | 3 | 48.95 | 11 | Amidation |
| NBs | PBC32914.1 | NVGTLARDFALPPa | 39.83 | 1368.751 | 1.4 | 685.3839 | 2 | 44 | 19 | Amidation |
| NBs | PBC32914.1 | SISSLARTGDLPVREQ | 39.66 | 1727.917 | 1.5 | 576.9803 | 3 | 23.16 | 8 |  |
| NBs | PBC32914.1 | YVASLARTGDLPIRa | 36.12 | 1529.868 | 0.5 | 510.9635 | 3 | 22.56 | 6 | Amidation |
| NBs | PBC32914.1 | NVASLARTYTLPQNAa | 34.95 | 1616.863 | 1.2 | 809.4399 | 2 | 30.41 | 7 | Amidation |
| NBs | PBC32914.1 | GIFVPGSVILRALSRQa | 42.55 | 1711.026 | 1.8 | 856.5216 | 2 | 70.58 | 13 | Amidation |
| NBs | PBC32914.1 | SVSSLAKNSAWPVSL | 38.37 | 1544.82 | 1.6 | 773.4184 | 2 | 53.19 | 5 |  |
| NBs | PBC34787.1 | AYTYVSEYKRLPVYNFGIa | 51.99 | 2181.126 | 0.6 | 1091.571 | 2 | 58.6 | 7 | Amidation |
| NBs | PBC34787.1 | PNDMLSQRYHFGLa | 48.95 | 1575.762 | 0.4 | 788.8884 | 2 | 32.82 | 5 | Amidation |
| NBs | PBC34787.1 | AVHYSGGQPLGSKRPNDMLSQRYHFGLa | 47.29 | 3013.509 | 1.4 | 754.3856 | 4 | 26.02 | 14 | Amidation |
| NBs | PBC34787.1 | AVHYSGGQPLGS | 37.66 | 1171.562 | 0.7 | 586.7888 | 2 | 13.39 | 6 |  |
| NBs | PBC34787.1 | RQYSFGLa | 31.08 | 868.4555 | 0.6 | 435.2353 | 2 | 21.52 | 3 | Amidation |
| NBs | PBC34787.1 | WIDTNDNKRGRDYSFGLa | 28.42 | 2054.992 | 1.5 | 686.0057 | 3 | 26.77 | 3 | Amidation |
| NBs | PBC34787.1 | LDYLPVDNPAFH | 42.16 | 1399.677 | 1.7 | 700.847 | 2 | 52.91 | 3 |  |
| NBs | PBC34787.1 | YPLRLNLD | 32.63 | 1002.55 | 0.2 | 502.2823 | 2 | 35.29 | 3 |  |
| NBs | PBC34787.1 | GRDYSFGLa | 30.69 | 912.4453 | 0.4 | 457.2301 | 2 | 24.05 | 3 | Amidation |
| NBs | PBC34787.1 | GRQPYSFGLa | 30.68 | 1022.53 | 0.4 | 512.2723 | 2 | 23.69 | 5 | Amidation |
| NBs | XP_016905690.1 | LNSDSRNSQVNGYTPRLa | 44.7 | 1918.961 | 1.8 | 640.662 | 3 | 15.42 | 3 | Amidation |
| NBs | XP_016905690.1 | SNAPVSNLNFN | 42.02 | 1175.557 | 1.4 | 588.7867 | 2 | 30.68 | 3 |  |
| NBs | XP_016905690.1 | NSDSRNSQVNGYTPRLa | 40.74 | 1805.877 | 1.5 | 602.9671 | 3 | 14.5 | 3 | Amidation |
| NBs | XP_016905690.1 | RASGLLSYPRIa | 25.05 | 1230.72 | 0.3 | 411.2473 | 3 | 22.05 | 3 | Amidation |
| NBs | XP_016908608.1 | LTNYLATGHRTNGGPVI | 51.9 | 1782.938 | 1 | 892.477 | 2 | 24.82 | 11 |  |
| NBs | XP_016908608.1 | NLDEIDRVGWSGFV | 49.71 | 1605.779 | 2.2 | 803.8984 | 2 | 74.07 | 6 |  |
| NBs | XP_016908608.1 | LTNYLATGHRTNGGPVIRRFa | 35.11 | 2241.224 | 0.8 | 748.0826 | 3 | 18.66 | 14 | Amidation |
| NBs | XP_016908608.1 | NIDEIDRTAFDNFF | 48.19 | 1715.779 | 1 | 858.8976 | 2 | 83.39 | 6 |  |
| NBs | XP_016908970.1 | MVPVPVHHMADELLRSGPDTVI | 54.41 | 2412.229 | 0.5 | 1207.123 | 2 | 60.48 | 21 |  |
| NBs | XP_016908970.1 | VHHMADELLRSGPDTVI | 51.6 | 1888.947 | 0.8 | 945.4813 | 2 | 32.22 | 9 |  |
| NBs | XP_016908970.1 | MVPVPVHHMADEL | 33.03 | 1473.711 | 1.3 | 737.8636 | 2 | 27.55 | 4 |  |
| NBs | XP_016908970.1 | LRSGPDTVI | 25.3 | 956.5291 | 0.3 | 479.2719 | 2 | 16.69 | 3 |  |
| NBs | XP_016908970.1 | VPVPVHHMADELL | 47.4 | 1455.754 | 0.2 | 728.8846 | 2 | 32.18 | 6 |  |
| NBs | XP_016920932.1 | TWKSPDIVIRFa | 44.03 | 1359.766 | 0.2 | 454.2628 | 3 | 42.36 | 11 | Amidation |
| NBs | XP_016920932.1 | GRNDLNFIRYa | 42.19 | 1265.663 | 1.3 | 633.8395 | 2 | 23.39 | 5 | Amidation |
| PFs | PBC25365.1 | pQQFDDYGHLRFa | 36.55 | 1406.637 | -1.5 | 704.3246 | 2 | 56.2 | 15 | Pyro-glu from Q; Amidation |
| PFs | PBC27532.1 | LVDHRIPDLENEMF | 57.77 | 1726.835 | 2 | 864.4264 | 2 | 49.65 | 10 |  |
| PFs | PBC27532.1 | ISYDTYDERELSRDHPPLLL | 53.81 | 2431.202 | 1.3 | 811.4089 | 3 | 50.84 | 16 |  |
| PFs | PBC27532.1 | SLPLYGGNMSKTGDSRLKSEFE | 52.9 | 2415.174 | 1.1 | 806.0662 | 3 | 30.78 | 16 |  |
| PFs | PBC27532.1 | SLPLYGGNMSKTGDSRLKSE | 52.19 | 2139.063 | 1.2 | 1070.54 | 2 | 15.87 | 8 |  |
| PFs | PBC27532.1 | HPISYDTYDERELSRDHPPLLL | 51.83 | 2665.314 | 1.1 | 889.4461 | 3 | 40.68 | 31 |  |
| PFs | PBC27532.1 | IGSLSIVNSMDVLRQRVLLELARRKALQDQAQIDANRRLLETIa | 40.04 | 4913.782 | 2.3 | 983.766 | 5 | 87.46 | 19 | Amidation |
| PFs | PBC27532.1 | ARRKALQDQAQIDANRRLLETIa | 26.54 | 2577.458 | 0.2 | 516.4989 | 5 | 21.29 | 6 | Amidation |
| PFs | PBC27532.1 | LVDHRIPDLENEMFDSGNDPGSTVVRT | 72.85 | 3012.425 | 0.5 | 1005.149 | 3 | 50.35 | 25 |  |
| PFs | PBC27982.1 | ITGQGNRIF | 44.58 | 1004.54 | 0.3 | 503.2776 | 2 | 15.7 | 12 |  |
| PFs | PBC27982.1 | SLKAPFA | 33.45 | 732.417 | -0.4 | 367.2156 | 2 | 18.98 | 3 |  |
| PFs | PBC27985.1 | YLLSGKARYa | 30.64 | 1068.608 | 0.9 | 535.3118 | 2 | 10.92 | 4 | Amidation |
| PFs | PBC28057.1 | GNNRPVYIPQPRPPHP | 52.22 | 1837.97 | 0.5 | 919.9927 | 2 | 17.43 | 18 |  |
| PFs | PBC28057.1 | GNNRPVYIPQPRPPHPRL | 49.35 | 2107.155 | 0.4 | 703.3926 | 3 | 17.9 | 21 |  |
| PFs | PBC28057.1 | PVYIPQPRPPHP | 42.58 | 1396.762 | 0.4 | 466.5946 | 3 | 21.67 | 7 |  |
| PFs | PBC28214.1 | GLDLGLSRGFSGSQAAKHLMGLAAANYAGGPa | 63.47 | 2985.524 | 2.3 | 747.39 | 4 | 69.13 | 14 | Amidation |
| PFs | PBC28214.1 | GLDLGLSRGFSGSQAAKH | 45.58 | 1799.928 | 1.1 | 900.9721 | 2 | 22.16 | 8 |  |
| PFs | PBC28214.1 | GLDLGLSRGFSGSQAA | 58.47 | 1534.774 | 2.1 | 768.3959 | 2 | 42.52 | 5 |  |
| PFs | PBC28214.1 | GLDLGLSRGFSGSQAAKHLMa | 25.64 | 2043.068 | 0.4 | 682.0303 | 3 | 41.15 | 4 | Amidation |
| PFs | PBC28214.1 | HLMGLAAANYAGGPa | 41.31 | 1340.666 | 1.6 | 671.3413 | 2 | 26.72 | 8 | Amidation |
| PFs | PBC30406.1 | SDPHLSIGILSKPISAIPSSKFDD | 66.06 | 2523.322 | 1.2 | 842.1157 | 3 | 58.92 | 17 |  |
| PFs | PBC30406.1 | SQRSPSLRLRFa | 38.3 | 1344.774 | -0.2 | 449.2651 | 3 | 14.04 | 4 | Amidation |
| PFs | PBC30406.1 | SPSLRLRFa | 37.71 | 973.5821 | 0.2 | 487.7984 | 2 | 16.5 | 4 | Amidation |
| PFs | PBC30406.1 | SDPHLSIGILSKPISAIP | 27.97 | 1844.041 | 1.2 | 923.0287 | 2 | 63.92 | 8 |  |
| PFs | PBC30406.1 | SDPHLSIGILSKP | 37.51 | 1362.751 | -0.6 | 455.2573 | 3 | 31.93 | 9 |  |
| PFs | PBC31004.1 | pQMFTYSHGWTNa | 42.17 | 1352.561 | 1.5 | 677.2887 | 2 | 53.87 | 6 | Pyro-glu from Q; Amidation |
| PFs | PBC31004.1 | SFSENMINDHRQPASTNNNY | 56.41 | 2338.003 | -0.2 | 1170.009 | 2 | 20.18 | 7 |  |
| PFs | PBC31251.1 | AYRKPPFNGSIFa | 39.22 | 1394.746 | 1.1 | 698.381 | 2 | 23.96 | 9 | Amidation |
| PFs | PBC31251.1 | RKPPFNGSIFa | 27.47 | 1160.645 | 1.8 | 581.331 | 2 | 20.02 | 3 | Amidation |
| PFs | PBC31251.1 | KPPFNGSIFa | 25.66 | 1004.544 | 0.6 | 503.2798 | 2 | 29.89 | 3 | Amidation |
| PFs | PBC31431.1 | APVGYQEMQGKKNSASLNSENFGIF | 77.23 | 2715.296 | 0.2 | 1358.656 | 2 | 48.2 | 11 |  |
| PFs | PBC31431.1 | NSIINDVKNELFPEDIN | 57.78 | 1972.974 | 1.6 | 987.4959 | 2 | 84.36 | 25 |  |
| PFs | PBC31431.1 | ARMGFHGMRa | 51.41 | 1060.517 | 0.9 | 531.2663 | 2 | 6.72 | 21 | Amidation |
| PFs | PBC31431.1 | APMGFQGMRG | 46.15 | 1050.474 | 0.4 | 526.2444 | 2 | 19.43 | 3 |  |
| PFs | PBC31431.1 | STDFQDVESGSESFKRARMGFHGMRa | 45.36 | 2860.313 | 1.5 | 716.0867 | 4 | 24.71 | 5 | Amidation |
| PFs | PBC31431.1 | SPFRYLGV | 45.28 | 937.5021 | 0.6 | 469.7586 | 2 | 35.51 | 7 |  |
| PFs | PBC31431.1 | APMGFQGMRa | 44.07 | 992.4684 | -0.1 | 497.2415 | 2 | 15.49 | 6 | Amidation |
| PFs | PBC31431.1 | APMGFYGTRG | 41.45 | 1055.486 | 0.8 | 528.7506 | 2 | 18.36 | 3 |  |
| PFs | PBC31431.1 | ARMGFHGMRG | 40.87 | 1118.523 | -0.6 | 373.8479 | 3 | 9.39 | 3 |  |
| PFs | PBC31431.1 | ALMGFQGVRG | 39.1 | 1034.533 | 0.9 | 518.2743 | 2 | 25.78 | 3 |  |
| PFs | PBC31431.1 | APMGFYGTRa | 39.01 | 997.4803 | 0.6 | 499.7478 | 2 | 15.72 | 4 | Amidation |
| PFs | PBC31431.1 | ALMGFQGVRa | 37.61 | 976.5276 | 0.6 | 489.2714 | 2 | 22.47 | 4 | Amidation |
| PFs | PBC31431.1 | SPFRYLGVRa | 34.87 | 1092.619 | 0.9 | 547.3174 | 2 | 16.65 | 10 | Amidation |
| PFs | PBC31431.1 | ASFDDEYY | 26.92 | 1008.371 | 0.4 | 505.1931 | 2 | 32.7 | 4 |  |
| PFs | PBC31431.1 | ASFDDEYYKRAPMGFQGMRa | 54.61 | 2267.025 | 0.7 | 567.764 | 4 | 30.79 | 8 | Amidation |
| PFs | PBC31431.1 | STDFQDVESGSESF | 46.63 | 1533.611 | 1.5 | 767.8138 | 2 | 44.9 | 12 |  |
| PFs | PBC32274.1 | pQLHNIIDKPRQN | 47.32 | 1457.774 | 1.2 | 729.8951 | 2 | 16.62 | 6 | Pyro-glu from Q |
| PFs | PBC32274.1 | pQLHNIIDKPRQNFNDP | 40.55 | 1930.965 | 1.2 | 966.4909 | 2 | 26.43 | 6 | Pyro-glu from Q |
| PFs | PBC32274.1 | pQITQFTPRLa | 39.64 | 1084.603 | 1 | 543.3093 | 2 | 54 | 4 | Pyro-glu from Q; Amidation |
| PFs | PBC32274.1 | SGMWFGPRLa | 39.39 | 1048.528 | 1.1 | 525.2717 | 2 | 47.79 | 4 | Amidation |
| PFs | PBC32274.1 | RVPWTPSPRLa | 39.12 | 1206.699 | 0.6 | 604.3569 | 2 | 19.13 | 7 | Amidation |
| PFs | PBC32274.1 | VPWTPSPRLa | 37.19 | 1050.597 | 0.4 | 526.3062 | 2 | 24.2 | 4 | Amidation |
| PFs | PBC32274.1 | pQLHNIIDKPRQNFNDPRF | 30.58 | 2234.135 | 1.6 | 559.5418 | 4 | 32.43 | 4 | Pyro-glu from Q |
| PFs | PBC32274.1 | TSQDITSGMWFGPRLa | 46.98 | 1693.825 | 1.5 | 847.9208 | 2 | 62.81 | 10 | Amidation |
| PFs | PBC32274.1 | SQDITSGMWFGPRLa | 39.1 | 1592.777 | 1.5 | 797.397 | 2 | 66.63 | 4 | Amidation |
| PFs | PBC32274.1 | pQLHNIIDKP | 37.74 | 1059.571 | 0.2 | 530.793 | 2 | 24.59 | 3 | Pyro-glu from Q |
| PFs | PBC32274.1 | DITSGMWFGPRLa | 36.73 | 1377.686 | 1.7 | 689.8516 | 2 | 76.96 | 4 | Amidation |
| PFs | PBC32274.1 | GMWFGPRLa | 25.78 | 961.4956 | 0.9 | 481.7555 | 2 | 49.4 | 6 | Amidation |
| PFs | PBC32496.1 | IPAADKERLLN | 44.95 | 1238.698 | -0.1 | 620.3563 | 2 | 15.01 | 4 |  |
| PFs | PBC32496.1 | LRNQLDIGDLQ | 43.76 | 1283.683 | 2.6 | 642.8506 | 2 | 30.89 | 5 |  |
| PFs | PBC32496.1 | SYWKQCAFNAVSCFa | 41.11 | 1651.728 | 1.9 | 826.8726 | 2 | 69.18 | 7 | Amidation |
| PFs | PBC32545.1 | NSELINSLLGLPKNMNNAa | 50.59 | 1940.015 | 1.2 | 971.0159 | 2 | 72.01 | 11 | Amidation |
| PFs | PBC32608.1 | IDLSRFYGHFNT | 52.52 | 1468.71 | 1.3 | 735.3632 | 2 | 47.67 | 11 |  |
| PFs | PBC32608.1 | IDLSRFYGHF | 45.01 | 1253.619 | 0.3 | 627.8171 | 2 | 52.78 | 9 |  |
| PFs | PBC32608.1 | IDLSRFYGHFNTKR | 38.28 | 1752.906 | 0.4 | 439.2339 | 4 | 27.86 | 5 |  |
| PFs | PBC32608.1 | DLSRFYGHF | 26.3 | 1140.535 | 0.7 | 571.2753 | 2 | 35.46 | 8 |  |
| PFs | PBC32608.1 | IDLSRFYGHFNTK | 34.1 | 1596.805 | -0.9 | 533.2751 | 3 | 35.46 | 10 |  |
| PFs | PBC32678.1 | pQDVDHVFLRFa | 43.61 | 1256.63 | 1.4 | 629.3232 | 2 | 60.92 | 9 | Pyro-glu from Q; Amidation |
| PFs | PBC32678.1 | pQDVDHVFLR | 36.49 | 1110.546 | -0.8 | 556.2797 | 2 | 32.82 | 3 | Pyro-glu from Q |
| PFs | PBC32678.1 | QDVDHVFLRFa | 30.34 | 1273.657 | 2.2 | 637.837 | 2 | 35.62 | 10 | Amidation |
| PFs | PBC32727.1 | SQAYDPYSNAAQFQLSSQSRGYPYQHRL | 62.05 | 3261.523 | 1.3 | 816.389 | 4 | 38.35 | 9 |  |
| PFs | PBC32727.1 | LPTNLGEDTKKTEQTMRPKS | 55.51 | 2273.169 | 1.6 | 569.3003 | 4 | 15.34 | 14 |  |
| PFs | PBC32727.1 | YPYQHRLIY | 44.17 | 1251.64 | 0.7 | 626.8277 | 2 | 19.76 | 5 |  |
| PFs | PBC32727.1 | NVPIYQEPRF | 40.73 | 1261.646 | 0.6 | 631.8304 | 2 | 32.28 | 3 |  |
| PFs | PBC32727.1 | SQAYDPYSNAAQFQLSSQSRGYPYQHRLIY | 67.17 | 3537.67 | 1.4 | 885.4261 | 4 | 48.14 | 22 |  |
| PFs | PBC32727.1 | SQAYDPYSNAAQFQLSSQSRGYPYQHRLI | 42.19 | 3374.607 | 1.3 | 844.66 | 4 | 46.04 | 3 |  |
| PFs | PBC32727.1 | VPIYQEPRF | 39.88 | 1147.603 | 0.8 | 574.809 | 2 | 30.27 | 4 |  |
| PFs | PBC32727.1 | GYPYQHRLIY | 35.91 | 1308.662 | 1.2 | 655.3388 | 2 | 20.49 | 7 |  |
| PFs | PBC32914.1 | SIATLAKNDDLPISLHDRMAENEDDEE | 66.67 | 3040.393 | 0.9 | 1014.473 | 3 | 41.42 | 13 |  |
| PFs | PBC32914.1 | FLLLPATDNNYFHQKLPSSLRSKSL | 51.8 | 2888.555 | 0.9 | 963.8597 | 3 | 53.93 | 7 |  |
| PFs | PBC32914.1 | YVASLARTGDLPIRGQ | 48.79 | 1715.932 | 0.7 | 858.9738 | 2 | 24.81 | 12 |  |
| PFs | PBC32914.1 | NVGSVAREHGLPYa | 47.94 | 1396.721 | 1 | 699.3685 | 2 | 15.44 | 7 | Amidation |
| PFs | PBC32914.1 | NVASLARTYTLPQNAa | 47.62 | 1616.863 | 1.3 | 809.44 | 2 | 30.12 | 4 | Amidation |
| PFs | PBC32914.1 | NIASLIRDYDQSRENRVSFPa | 46.82 | 2378.209 | 0.5 | 793.744 | 3 | 48.39 | 10 | Amidation |
| PFs | PBC32914.1 | SISSLARTGDLPVREQ | 45.9 | 1727.917 | 1.2 | 576.9802 | 3 | 23.96 | 9 |  |
| PFs | PBC32914.1 | NVGTLARDFALPPa | 45.12 | 1368.751 | 1.3 | 685.3839 | 2 | 44.83 | 20 | Amidation |
| PFs | PBC32914.1 | YVASLARTGDLPIRa | 37.25 | 1529.868 | 0.5 | 510.9634 | 3 | 22.77 | 6 | Amidation |
| PFs | PBC32914.1 | SVSSLAKNSAWPVSL | 47.07 | 1544.82 | 2.4 | 773.419 | 2 | 52.64 | 6 |  |
| PFs | PBC32914.1 | GIFVPGSVILRALSRQa | 38.99 | 1711.026 | 0.9 | 571.3497 | 3 | 69.51 | 19 | Amidation |
| PFs | PBC34787.1 | AYTYVSEYKRLPVYNFGIa | 59.77 | 2181.126 | 0.7 | 1091.571 | 2 | 58.48 | 9 | Amidation |
| PFs | PBC34787.1 | PNDMLSQRYHFGLa | 59.21 | 1575.762 | 1 | 788.8889 | 2 | 32.45 | 8 | Amidation |
| PFs | PBC34787.1 | AVHYSGGQPLGSKRPNDMLSQRYHFGLa | 50.41 | 3013.509 | 0.8 | 1005.511 | 3 | 26.04 | 12 | Amidation |
| PFs | PBC34787.1 | AVHYSGGQPLGS | 38.4 | 1171.562 | 1 | 586.7889 | 2 | 13.02 | 5 |  |
| PFs | PBC34787.1 | RQYSFGLa | 33.4 | 868.4555 | -0.2 | 435.235 | 2 | 19.76 | 3 | Amidation |
| PFs | PBC34787.1 | WIDTNDNKRGRDYSFGLa | 30.34 | 2054.992 | 1.4 | 686.0056 | 3 | 26.1 | 4 | Amidation |
| PFs | PBC34787.1 | LDYLPVDNPAFH | 48.74 | 1399.677 | 1 | 700.8466 | 2 | 53.02 | 6 |  |
| PFs | PBC34787.1 | GRQPYSFGLa | 33.85 | 1022.53 | 1.3 | 512.2728 | 2 | 23.75 | 5 | Amidation |
| PFs | PBC34787.1 | GRDYSFGLa | 29.14 | 912.4453 | 0 | 457.2299 | 2 | 23.11 | 7 | Amidation |
| PFs | PBC34787.1 | YPLRLNLD | 28.83 | 1002.55 | 0.5 | 502.2824 | 2 | 34.88 | 3 |  |
| PFs | XP_016905690.1 | LNSDSRNSQVNGYTPRLa | 51.57 | 1918.961 | 1.2 | 640.6617 | 3 | 15.11 | 5 | Amidation |
| PFs | XP_016905690.1 | NSDSRNSQVNGYTPRLa | 49.71 | 1805.877 | 1.4 | 602.967 | 3 | 14.65 | 4 | Amidation |
| PFs | XP_016905690.1 | RASGLLSYPRIa | 37.06 | 1230.72 | 1.3 | 616.3679 | 2 | 20.79 | 4 | Amidation |
| PFs | XP_016908608.1 | LTNYLATGHRTNGGPVI | 61.58 | 1782.938 | -0.6 | 892.4755 | 2 | 24.48 | 7 |  |
| PFs | XP_016908608.1 | NLDEIDRVGWSGFV | 55.78 | 1605.779 | 1.8 | 803.8981 | 2 | 73.08 | 9 |  |
| PFs | XP_016908608.1 | LTNYLATGHRTNGGPVIRRFa | 38.36 | 2241.224 | 0.2 | 449.2522 | 5 | 15.5 | 13 | Amidation |
| PFs | XP_016908608.1 | NIDEIDRTAFDNFF | 54.7 | 1715.779 | 0.8 | 858.8975 | 2 | 83.7 | 6 |  |
| PFs | XP_016908970.1 | MVPVPVHHMADELLRSGPDTVI | 63.82 | 2412.229 | 1 | 1207.123 | 2 | 59.89 | 23 |  |
| PFs | XP_016908970.1 | VHHMADELLRSGPDTVI | 59.42 | 1888.947 | 1 | 945.4814 | 2 | 31.3 | 12 |  |
| PFs | XP_016908970.1 | MVPVPVHHMADEL | 38.66 | 1473.711 | 0.6 | 737.8632 | 2 | 26.32 | 4 |  |
| PFs | XP_016908970.1 | LRSGPDTVI | 33.3 | 956.5291 | 0.5 | 479.2721 | 2 | 16.34 | 3 |  |
| PFs | XP_016908970.1 | VPVPVHHMADELL | 33.21 | 1455.754 | 1.1 | 728.8853 | 2 | 31.73 | 13 |  |
| PFs | XP_016920932.1 | TWKSPDIVIRFa | 42.29 | 1359.766 | 0.3 | 454.2628 | 3 | 41.77 | 11 | Amidation |
| PFs | XP_016920932.1 | GRNDLNFIRYa | 46.06 | 1265.663 | 0.5 | 633.839 | 2 | 23.5 | 8 | Amidation |
| NFs | PBC25365.1 | pQQFDDYGHLRFa | 57.86 | 1406.637 | 1.1 | 704.3264 | 2 | 57.22 | 3 | Pyro-glu from Q; Amidation |
| NFs | PBC27532.1 | SLPLYGGNMSKTGDSRLKSEFE | 72.83 | 2415.174 | -1.7 | 1208.592 | 2 | 29.14 | 9 |  |
| NFs | PBC27532.1 | SLPLYGGNMSKTGDSRLKSE | 70.09 | 2139.063 | -1.1 | 1070.538 | 2 | 19.53 | 9 |  |
| NFs | PBC27532.1 | HPISYDTYDERELSRDHPPLLL | 51.4 | 2665.314 | 1.7 | 889.4467 | 3 | 40.88 | 12 |  |
| NFs | PBC27532.1 | ARRKALQDQAQIDANRRLLETIa | 50.38 | 2577.458 | 0.9 | 645.3723 | 4 | 21.77 | 9 | Amidation |
| NFs | PBC27532.1 | LVDHRIPDLENEMF | 49.87 | 1726.835 | 1.2 | 576.6196 | 3 | 48.67 | 5 |  |
| NFs | PBC27532.1 | ISYDTYDERELSRDHPPLLL | 47.14 | 2431.202 | -1.4 | 811.4067 | 3 | 49.77 | 5 |  |
| NFs | PBC27532.1 | IGSLSIVNSMDVLRQRVLLELARRKALQDQAQIDANRRLLETIa | 32.59 | 4913.782 | 2.9 | 819.9734 | 6 | 88.02 | 6 | Amidation |
| NFs | PBC27532.1 | LVDHRIPDLENEMFDSGNDPGSTVVRT | 69.38 | 3012.425 | 2.7 | 1005.152 | 3 | 56.97 | 3 |  |
| NFs | PBC27982.1 | ITGQGNRIF | 47.84 | 1004.54 | 1 | 503.2779 | 2 | 17.63 | 4 |  |
| NFs | PBC27982.1 | SLKAPFA | 35.82 | 732.417 | -1.3 | 367.2153 | 2 | 19.3 | 3 |  |
| NFs | PBC27985.1 | YLLSGKARYa | 28.55 | 1068.608 | 0.4 | 535.3115 | 2 | 11.92 | 6 | Amidation |
| NFs | PBC28057.1 | GNNRPVYIPQPRPPHP | 61.04 | 1837.97 | 0.9 | 613.6645 | 3 | 13.46 | 17 |  |
| NFs | PBC28057.1 | GNNRPVYIPQPRPPHPRL | 57.25 | 2107.155 | 1.7 | 703.3935 | 3 | 15.04 | 61 |  |
| NFs | PBC28057.1 | PVYIPQPRPPHP | 42.53 | 1396.762 | 0 | 466.5945 | 3 | 18.93 | 7 |  |
| NFs | PBC28214.1 | GLDLGLSRGFSGSQAAKHLMGLAAANYAGGPa | 48.64 | 2985.524 | 2.3 | 996.1843 | 3 | 69.28 | 8 | Amidation |
| NFs | PBC28214.1 | GLDLGLSRGFSGSQAA | 38.12 | 1534.774 | 1.7 | 768.3955 | 2 | 42.16 | 5 |  |
| NFs | PBC28214.1 | GLDLGLSRGFSGSQAAKH | 35.47 | 1799.928 | 0.4 | 450.9894 | 4 | 18.94 | 4 |  |
| NFs | PBC28214.1 | HLMGLAAANYAGGPa | 52.17 | 1340.666 | 0.2 | 671.3403 | 2 | 24.83 | 3 | Amidation |
| NFs | PBC28214.1 | GLDLGLSRGFSGSQAAKHLMa | 53.99 | 2985.524 | -1.5 | 1493.767 | 2 | 69.37 | 19 | Amidation |
| NFs | PBC30406.1 | SDPHLSIGILSKPISAIPSSKFDD | 66.11 | 2523.322 | -2.7 | 1262.665 | 2 | 57.71 | 9 |  |
| NFs | PBC30406.1 | SQRSPSLRLRFa | 41.59 | 1344.774 | 0.2 | 449.2653 | 3 | 13.35 | 8 | Amidation |
| NFs | PBC30406.1 | SPSLRLRFa | 33.76 | 973.5821 | 0.3 | 487.7985 | 2 | 17.83 | 3 | Amidation |
| NFs | PBC30406.1 | SDPHLSIGILSKPISAIP | 33.69 | 1844.041 | 3.3 | 923.0306 | 2 | 63.22 | 6 |  |
| NFs | PBC30406.1 | SDPHLSIGILSKP | 56.41 | 1362.751 | 0.6 | 682.3831 | 2 | 30.59 | 3 |  |
| NFs | PBC31004.1 | pQMFTYSHGWTNa | 35.37 | 1352.561 | 2.1 | 677.2891 | 2 | 54.56 | 16 | Pyro-glu from Q; Amidation |
| NFs | PBC31251.1 | AYRKPPFNGSIFa | 56.07 | 1394.746 | 1.2 | 698.381 | 2 | 20.1 | 23 | Amidation |
| NFs | PBC31251.1 | KPPFNGSIFa | 39.77 | 1004.544 | 0.3 | 503.2796 | 2 | 26.3 | 8 | Amidation |
| NFs | PBC31251.1 | RKPPFNGSIFa | 28.99 | 1160.645 | 0.6 | 581.3303 | 2 | 20.94 | 6 | Amidation |
| NFs | PBC31431.1 | APVGYQEMQGKKNSASLNSENFGIF | 76.23 | 2715.296 | 0.9 | 1358.657 | 2 | 47.19 | 9 |  |
| NFs | PBC31431.1 | NSIINDVKNELFPEDIN | 61.58 | 1972.974 | 1.8 | 987.4961 | 2 | 85.69 | 17 |  |
| NFs | PBC31431.1 | ARMGFHGMRa | 56.1 | 1060.517 | 0.4 | 531.2661 | 2 | 8.49 | 4 | Amidation |
| NFs | PBC31431.1 | STDFQDVESGSESFKRARMGFHGMRa | 52.07 | 2860.313 | -0.9 | 477.7257 | 6 | 24.04 | 8 | Amidation |
| NFs | PBC31431.1 | SPFRYLGV | 49.43 | 937.5021 | 0.4 | 469.7585 | 2 | 34.68 | 6 |  |
| NFs | PBC31431.1 | APMGFQGMRG | 46.35 | 1050.474 | 1 | 526.2448 | 2 | 19.72 | 4 |  |
| NFs | PBC31431.1 | ARMGFHGMRG | 45.29 | 1118.523 | -0.7 | 373.8479 | 3 | 9.3 | 3 |  |
| NFs | PBC31431.1 | APMGFYGTRa | 45.15 | 997.4803 | 0.5 | 499.7477 | 2 | 15.19 | 4 | Amidation |
| NFs | PBC31431.1 | APMGFYGTRG | 44.23 | 1055.486 | 0.1 | 528.7502 | 2 | 17.78 | 3 |  |
| NFs | PBC31431.1 | APMGFQGMRa | 43.83 | 992.4684 | -0.1 | 497.2414 | 2 | 16.14 | 3 | Amidation |
| NFs | PBC31431.1 | ALMGFQGVRG | 42.24 | 1034.533 | 0 | 518.2738 | 2 | 25.21 | 3 |  |
| NFs | PBC31431.1 | ALMGFQGVRa | 39.26 | 976.5276 | 1.2 | 489.2717 | 2 | 22.01 | 3 | Amidation |
| NFs | PBC31431.1 | SPFRYLGVRa | 36.21 | 1092.619 | -0.1 | 365.2137 | 3 | 17.16 | 3 | Amidation |
| NFs | PBC31431.1 | ASFDDEYY | 24.23 | 1008.371 | 0.2 | 505.193 | 2 | 32.63 | 3 |  |
| NFs | PBC31431.1 | ASFDDEYYKRAPMGFQGMRa | 64.73 | 2267.025 | 1.2 | 567.7642 | 4 | 26.98 | 10 | Amidation |
| NFs | PBC31431.1 | STDFQDVESGSESF | 43.46 | 1533.611 | 1.2 | 767.8135 | 2 | 45.7 | 8 |  |
| NFs | PBC32274.1 | RVPWTPSPRLa | 42.45 | 1206.699 | 1.2 | 604.3572 | 2 | 18.98 | 4 | Amidation |
| NFs | PBC32274.1 | pQLHNIIDKPRQN | 41.7 | 1457.774 | 3.2 | 729.8966 | 2 | 15.91 | 4 | Pyro-glu from Q |
| NFs | PBC32274.1 | pQLHNIIDKPRQNFNDPRF | 33.79 | 2234.135 | 1.6 | 745.72 | 3 | 31.38 | 4 | Pyro-glu from Q |
| NFs | PBC32274.1 | pQITQFTPRLa | 30.83 | 1084.603 | 1 | 543.3093 | 2 | 53.25 | 9 | Pyro-glu from Q; Amidation |
| NFs | PBC32274.1 | pQLHNIIDKPRQNFNDP | 28.77 | 1930.965 | -2.1 | 966.4877 | 2 | 25.76 | 5 | Pyro-glu from Q |
| NFs | PBC32274.1 | SGMWFGPRLa | 28 | 1048.528 | -0.7 | 525.2707 | 2 | 47.79 | 12 | Amidation |
| NFs | PBC32274.1 | VPWTPSPRLa | 27.27 | 1050.597 | 1.5 | 526.3068 | 2 | 23.67 | 3 | Amidation |
| NFs | PBC32274.1 | DITSGMWFGPRLa | 43.5 | 1377.686 | 1.1 | 689.8512 | 2 | 91.87 | 4 | Amidation |
| NFs | PBC32274.1 | GMWFGPRLa | 26.94 | 961.4956 | 0.8 | 481.7555 | 2 | 47.5 | 7 | Amidation |
| NFs | PBC32274.1 | pQLHNIIDKP | 33.03 | 1059.571 | 0.7 | 530.7933 | 2 | 20.67 | 12 | Pyro-glu from Q |
| NFs | PBC32274.1 | SQDITSGMWFGPRLa | 48.18 | 1592.777 | 1 | 797.3965 | 2 | 75.27 | 4 | Amidation |
| NFs | PBC32274.1 | TSQDITSGMWFGPRLa | 54.49 | 1693.825 | 1.1 | 847.9205 | 2 | 72.22 | 5 | Amidation |
| NFs | PBC32496.1 | LRNQLDIGDLQ | 48.94 | 1283.683 | 1.5 | 642.8499 | 2 | 30.84 | 5 |  |
| NFs | PBC32496.1 | IPAADKERLLN | 46.28 | 1238.698 | 0.4 | 620.3566 | 2 | 13.93 | 5 |  |
| NFs | PBC32496.1 | SYWKQCAFNAVSCFa | 38.98 | 1651.728 | 1.1 | 826.8719 | 2 | 70.16 | 9 | Amidation |
| NFs | PBC32545.1 | NSELINSLLGLPKNMNNAa | 37.17 | 1940.015 | 2.1 | 971.0167 | 2 | 71.55 | 3 | Amidation |
| NFs | PBC32608.1 | IDLSRFYGHF | 50.48 | 1253.619 | 1.4 | 627.8178 | 2 | 51.77 | 4 |  |
| NFs | PBC32608.1 | IDLSRFYGHFNT | 44.72 | 1468.71 | 1 | 735.3629 | 2 | 47.98 | 3 |  |
| NFs | PBC32608.1 | IDLSRFYGHFNTKR | 37.96 | 1752.906 | -0.2 | 585.3091 | 3 | 26.76 | 4 |  |
| NFs | PBC32608.1 | DLSRFYGHF | 24.76 | 1140.535 | 1.2 | 571.2755 | 2 | 34.83 | 3 |  |
| NFs | PBC32608.1 | IDLSRFYGHFNTK | 28.71 | 1596.805 | 1.3 | 799.4107 | 2 | 36.19 | 9 |  |
| NFs | PBC32678.1 | pQDVDHVFLR | 49.86 | 1110.546 | 0.4 | 556.2804 | 2 | 30.38 | 5 | Pyro-glu from Q |
| NFs | PBC32678.1 | pQDVDHVFLRFa | 54.14 | 1256.63 | 1 | 629.3229 | 2 | 67.87 | 16 | Pyro-glu from Q; Amidation |
| NFs | PBC32678.1 | QDVDHVFLRFa | 54.26 | 1273.657 | 0.8 | 637.8362 | 2 | 32.44 | 3 | Amidation |
| NFs | PBC32727.1 | LPTNLGEDTKKTEQTMRPKS | 61.45 | 2273.169 | -0.7 | 1137.591 | 2 | 14.32 | 14 |  |
| NFs | PBC32727.1 | SQAYDPYSNAAQFQLSSQSRGYPYQHRL | 55.41 | 3261.523 | 0.5 | 1088.182 | 3 | 37.25 | 3 |  |
| NFs | PBC32727.1 | NVPIYQEPRF | 46.81 | 1261.646 | 0.4 | 631.8303 | 2 | 31.89 | 4 |  |
| NFs | PBC32727.1 | YPYQHRLIY | 20.47 | 1251.64 | 1.1 | 418.2211 | 3 | 20.5 | 3 |  |
| NFs | PBC32727.1 | GYPYQHRLIY | 20.84 | 1308.662 | 0.3 | 437.2279 | 3 | 15.38 | 11 |  |
| NFs | PBC32727.1 | SQAYDPYSNAAQFQLSSQSRGYPYQHRLI | 35.98 | 3374.607 | -0.2 | 1125.876 | 3 | 48.52 | 20 |  |
| NFs | PBC32727.1 | SQAYDPYSNAAQFQLSSQSRGYPYQHRLIY | 74.39 | 3537.67 | 1 | 885.4257 | 4 | 55.74 | 10 |  |
| NFs | PBC32727.1 | VPIYQEPRF | 46.09 | 1147.603 | 1.1 | 574.8092 | 2 | 27.03 | 10 |  |
| NFs | PBC32914.1 | SIATLAKNDDLPISLHDRMAENEDDEE | 79.73 | 3040.393 | -0.6 | 1014.471 | 3 | 41.3 | 7 |  |
| NFs | PBC32914.1 | NVASLARTYTLPQNAa | 58.15 | 1616.863 | 1.6 | 809.4402 | 2 | 28.93 | 3 | Amidation |
| NFs | PBC32914.1 | NVGSVAREHGLPYa | 56.28 | 1396.721 | 0.6 | 699.3682 | 2 | 16.87 | 6 | Amidation |
| NFs | PBC32914.1 | YVASLARTGDLPIRGQ | 53.24 | 1715.932 | 1.1 | 572.9852 | 3 | 25.03 | 9 |  |
| NFs | PBC32914.1 | FLLLPATDNNYFHQKLPSSLRSKSL | 51.67 | 2888.555 | 2.3 | 723.1476 | 4 | 52.88 | 4 |  |
| NFs | PBC32914.1 | SISSLARTGDLPVREQ | 49.75 | 1727.917 | 2.1 | 576.9807 | 3 | 22.94 | 6 |  |
| NFs | PBC32914.1 | NIASLIRDYDQSRENRVSFPa | 47.5 | 2378.209 | 0.8 | 793.7443 | 3 | 47.24 | 6 | Amidation |
| NFs | PBC32914.1 | YVASLARTGDLPIRa | 26.48 | 1529.868 | 1.1 | 765.942 | 2 | 22.24 | 3 | Amidation |
| NFs | PBC32914.1 | NVGTLARDFALPPa | 19.92 | 1368.751 | 0.2 | 685.3831 | 2 | 43.82 | 4 | Amidation |
| NFs | PBC32914.1 | GIFVPGSVILRALSRQa | 48.99 | 1711.026 | 0.9 | 571.3497 | 3 | 83.9 | 14 | Amidation |
| NFs | PBC32914.1 | SVSSLAKNSAWPVSL | 48.47 | 1544.82 | 1.4 | 773.4183 | 2 | 57.72 | 4 |  |
| NFs | PBC34787.1 | PNDMLSQRYHFGLa | 66.58 | 1575.762 | 0.1 | 788.8882 | 2 | 31.07 | 4 | Amidation |
| NFs | PBC34787.1 | AVHYSGGQPLGSKRPNDMLSQRYHFGLa | 53.14 | 3013.509 | 1.1 | 754.3854 | 4 | 24.91 | 11 | Amidation |
| NFs | PBC34787.1 | AYTYVSEYKRLPVYNFGIa | 49.81 | 2181.126 | -0.5 | 1091.57 | 2 | 57.15 | 4 | Amidation |
| NFs | PBC34787.1 | AVHYSGGQPLGS | 38.77 | 1171.562 | 0.6 | 586.7887 | 2 | 13.17 | 3 |  |
| NFs | PBC34787.1 | WIDTNDNKRGRDYSFGLa | 37 | 2054.992 | 0.6 | 686.0051 | 3 | 24.87 | 6 | Amidation |
| NFs | PBC34787.1 | RQYSFGLa | 32.4 | 868.4555 | -0.3 | 435.2349 | 2 | 19.73 | 3 | Amidation |
| NFs | PBC34787.1 | GRQPYSFGLa | 32.58 | 1022.53 | 0.5 | 512.2724 | 2 | 18.09 | 3 | Amidation |
| NFs | PBC34787.1 | YPLRLNLD | 34.51 | 1002.55 | 0.3 | 502.2823 | 2 | 32.93 | 8 |  |
| NFs | PBC34787.1 | LDYLPVDNPAFH | 40.13 | 1399.677 | 2.2 | 700.8474 | 2 | 53.16 | 14 |  |
| NFs | PBC34787.1 | GRDYSFGLa | 20.29 | 912.4453 | 0 | 457.2299 | 2 | 22.83 | 13 | Amidation |
| NFs | XP_016905690.1 | RASGLLSYPRIa | 36.91 | 1230.72 | 1.6 | 616.368 | 2 | 17.49 | 5 | Amidation |
| NFs | XP_016905690.1 | LNSDSRNSQVNGYTPRLa | 43.12 | 1918.961 | 0.8 | 640.6614 | 3 | 15.95 | 22 | Amidation |
| NFs | XP_016905690.1 | NSDSRNSQVNGYTPRLa | 30.46 | 1805.877 | 2.7 | 602.9678 | 3 | 14.09 | 18 | Amidation |
| NFs | XP_016908608.1 | LTNYLATGHRTNGGPVI | 68.44 | 1782.938 | 1.1 | 892.4771 | 2 | 23.49 | 5 |  |
| NFs | XP_016908608.1 | NLDEIDRVGWSGFV | 65.24 | 1605.779 | 1.8 | 803.8981 | 2 | 73.95 | 5 |  |
| NFs | XP_016908608.1 | LTNYLATGHRTNGGPVIRRFa | 38.87 | 2241.224 | 0.5 | 449.2524 | 5 | 17.43 | 9 | Amidation |
| NFs | XP_016908608.1 | NIDEIDRTAFDNFF | 41.27 | 1715.779 | 2.6 | 858.899 | 2 | 96.53 | 8 |  |
| NFs | XP_016908970.1 | MVPVPVHHMADELLRSGPDTVI | 64.98 | 2412.229 | -0.6 | 1207.121 | 2 | 60.24 | 11 |  |
| NFs | XP_016908970.1 | VHHMADELLRSGPDTVI | 57.42 | 1888.947 | -0.9 | 945.4797 | 2 | 30.16 | 4 |  |
| NFs | XP_016908970.1 | MVPVPVHHMADEL | 38.96 | 1473.711 | 1.1 | 737.8635 | 2 | 26.34 | 6 |  |
| NFs | XP_016908970.1 | LRSGPDTVI | 33.48 | 956.5291 | 0.2 | 479.2719 | 2 | 15.76 | 3 |  |
| NFs | XP_016908970.1 | VPVPVHHMADELL | 31.46 | 1455.754 | 0.5 | 486.259 | 3 | 33.15 | 4 |  |
| NFs | XP_016920932.1 | TWKSPDIVIRFa | 49.3 | 1359.766 | 0.3 | 454.2628 | 3 | 40.95 | 9 | Amidation |
| NFs | XP_016920932.1 | GRNDLNFIRYa | 47.92 | 1265.663 | -0.1 | 422.8949 | 3 | 17.89 | 3 | Amidation |
