# Supplementary material for: Tachykinin signaling inhibits task-specific behavioral responsiveness in honeybee workers
Source: eLife. 2021 Mar 24;10:e64830. doi: 10.7554/eLife.64830 (PMC8016481; doi:10.7554/eLife.64830)
Supplement: Supplementary file 4. [file elife-64830-supp4.docx]

Efficiencies of dsRNA-mediated knockdown of *TRP* and *TRPR*. (manuscript section 2.3.2)


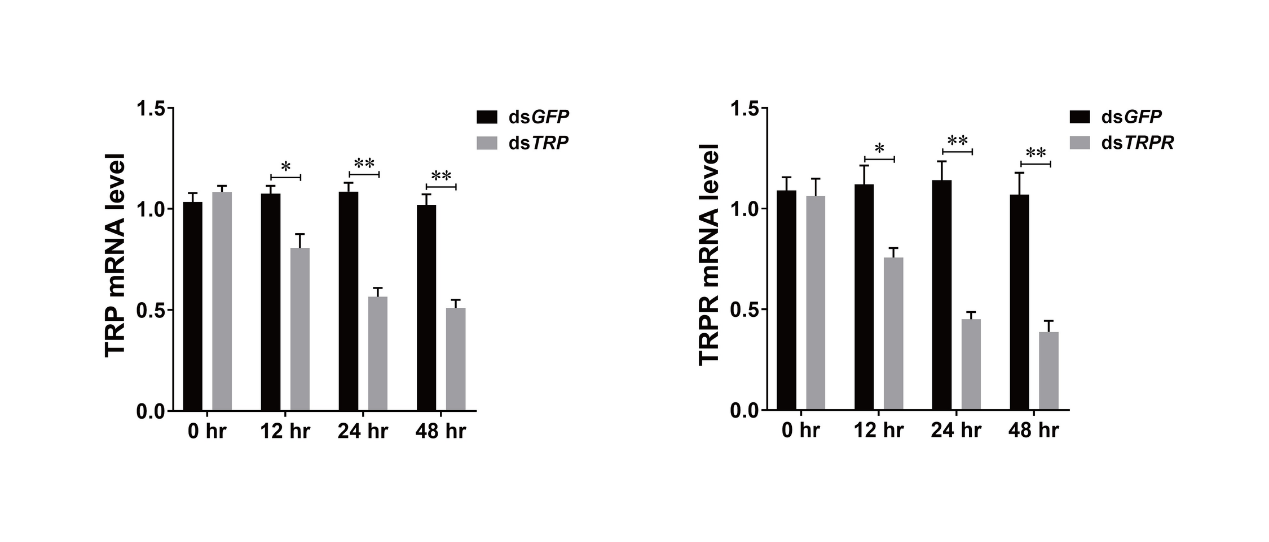


**Efficiencies of dsRNA-mediated knockdown of *TRP* and *TRPR*.** dsRNA (200 ng/bee for *TRP*, 2 μg/bee for *TRPR*) was microinjected into the head of honeybees via the central ocellus using a microinjector. dsRNA of green fluorescent protein gene (ds*GFP*, 2 μg/bee) was used as control. At 0, 12, 24, and 48 hours after injection, a group of 6 individual bees were collected from each injection group. Three independent replicate groups per condition were collected and qRT-PCR was performed to calculate the RNAi efficiency. Student’s t-tests were used for pairwise comparisons (*: *p* < 0.05, **: *p* < 0.01, ***: *p* < 0.001).
