# Supplementary material for: Tachykinin signaling inhibits task-specific behavioral responsiveness in honeybee workers
Source: eLife. 2021 Mar 24;10:e64830. doi: 10.7554/eLife.64830 (PMC8016481; doi:10.7554/eLife.64830)
Supplement: Supplementary file 5. [file elife-64830-supp5.docx]

Statistical differences in sucrose responsiveness after injection of ds*GFP*, ds*TRP*, and ds*TRPR*. (manuscript section 2.3.2)

| **Concentration** | **0.10%** | **0.30%** | **1.00%** | **3.00%** | **10.00%** | **30.00%** |
| --- | --- | --- | --- | --- | --- | --- |
| **Pollen foragers** |  |  |  |  |  |  |
| ds*TRP* vs ds*GFP* | * | * | * | ns | ns | ns |
| ds*TRPR* vs ds*GFP* | * | * | * | ns | ns | ns |
| ds*TRP* vs ds*TRPR* | ns | ns | ns | ns | ns | ns |
| **Nectar foragers** |  |  |  |  |  |  |
| ds*TRP* vs ds*GFP* | * | * | ** | * | ns | ns |
| ds*TRPR* vs ds*GFP* | * | * | * | ** | ns | ns |
| ds*TRP* vs ds*TRPR* | ns | ns | ns | ns | ns | ns |
| **Nurse bees** |  |  |  |  |  |  |
| ds*TRP* vs ds*GFP* | ns | ns | ns | ns | ns | ns |
| ds*TRPR* vs ds*GFP* | ns | ns | ns | ns | ns | ns |
| ds*TRP* vs ds*TRPR* | ns | ns | ns | ns | ns | ns |
| ns = *p* > 0.05, *: *p* < 0.05, **: *p* < 0.01 | | | | | | |
