# Supplementary material for: Tachykinin signaling inhibits task-specific behavioral responsiveness in honeybee workers
Source: eLife. 2021 Mar 24;10:e64830. doi: 10.7554/eLife.64830 (PMC8016481; doi:10.7554/eLife.64830)
Supplement: Supplementary file 6. [file elife-64830-supp6.docx]

Sequence information of primers used in this study. (manuscript section 2.4.1)

| **Gene name** | **Forward primer (5’-3’)** | **Reverse primer (5’-3’)** |
| --- | --- | --- |
| ***TRPR* clone (for FLAG-tag expression vectors)** | AAGCTTAAGCTTATGCAGACCGTAGAAGTTTTTCTAAAC | GGATCCTCAAGACACGTGACCCGTAGTTTGCGA |
| ***TRPR* clone (for EGFP-tag expression vectors)** | AAGCTTGCCACCATGCAGACCGTAGAAGTTTTTCTA | GGATCCAGACACGTGACCCGTAGTTTGC |
| ***TRPR* RNAi** | TAATACGACTCACTATAGGGGAGCAAACGAAGGGTGGTAA | TAATACGACTCACTATAGGGCGCGTCGAAATCTGGAGT |
| ***TRPR* qPCR** | GAGCAAACGAAGGGTGGTAA | ACTCCAGATTTCGACGCG |
| ***TRP* RNAi** | TAATACGACTCACTATAGGGGGTGTGCGTGGAAAGAAAAA | TAATACGACTCACTATAGGGTTTGATATCCATCCATCGACAA |
| ***TRP* qPCR** | GTTATCAAGATATGAGGAAT | ATGGATTAGAAGACAGTT |
| ***GFP* RNAi** | TAATACGACTCACTATAGGGAGTGGAGAGGGTGAAGGTGA | TAATACGACTCACTATAGGGGGTAAAAGGACAGGGCCATC |

Red font indicates T7 promoter sequence.
